# Supplementary material for: Old and newly synthesized histones are asymmetrically distributed in Drosophila intestinal stem cell divisions
Source: EMBO Rep. 2023 May 31;24(7):e56404. doi: 10.15252/embr.202256404 (PMC10328082; doi:10.15252/embr.202256404)
Supplement: Supplementary file 1 — Expanded View Figures PDF [file EMBR-24-e56404-s014.pdf]

Expanded View Figures

Figure EV1. Related to Figs 1 and 2: Histone transgene expression with the heat-shock-induced genetic switch over time with different histones and the development of a new histone co-expression construct, as well as the comparison of Pearson versus Spearman correlation coefficients in determining the colocalization of GFP-tagged and mCherry- or mKO-tagged histones in prophase and prometaphase ISCs.

A Time course of expression of H3-eGFP and H3-mCherry related to the heat shock. With no heat shock, only H3-eGFP is expressed. 12 h after the heat shock, some H3-mCherry is being expressed. By 24 h, robust expression of both H3-eGFP and H3-mCherry can be observed. After 36 h, H3-mCherry is predominantly observed, with minimal H3-eGFP remaining.

B Expression of H3-eGFP and H3-mCherry compared with H3.3-eGFP and H3.3-mCherry 12 h after heat shock. After 12 h, minimal H3-mCherry has been synthesized and incorporated into chromatin; however, H3.3 shows robust synthesis and incorporation of H3.3-mCherry. This is likely due to mode of incorporation, as H3.3 is incorporated into chromatin in a replication-independent manner, while H3 requires DNA replication for incorporation.

C Quantification of the number of ISCs expressing new H3-mCherry with no heat shock and 18 h after heat shock. Consistent square regions of 150 μm by 150 μm were analyzed in midguts with no heat shock and 18-h post-heat shock. With no heat shock, minimal ISCs expressing H3-mCherry were observed, and at 18 h after heat shock, most ISCs were expressing H3-mCherry (no heat shock, % of ISCs = 2.42 ± 1.39%, n = 25 regions, N = 5 midguts; 18-h post-heat shock, % of ISCs = 95.06 ± 1.52%, n = 25 regions, N = 5 midguts).

D The T2A histone transgene design: *esg-Gal4* > *UASp-H3-eGFP-T2A-H3-mCherry-PolyA*. This design allows for both H3-eGFP and H3-mCherry to be expressed at the same time and in equal molar ratios, utilizing the self-cleaving T2A peptide in between *H3-eGFP* and *H3-mCherry* sequences (see Materials and Methods).

E A western blot targeting eGFP in protein extracted from intestines expressing the *UASp-H3-eGFP-T2A-H3-mCherry-PolyA* transgene. One band can be observed at ~40 kDa, corresponding to H3-eGFP. The uncleaved protein of H3-eGFP-H3-mCherry would be observed at ~80 kDa, indicating the self-cleaving T2A peptide is efficiently cleaving.

F Comparison of Pearson and Spearman correlation coefficients to determine the colocalization of eGFP- and mCherry- or mKO-tagged histones in prophase and prometaphase ISCs in different transgene designs. Spearman correlation coefficients are slightly different when compared to Pearson correlation coefficients. There are no significant differences between Pearson and Spearman correlation coefficients for H4 [avg. Pearson correlation coefficient for H4 = 0.51 ± 0.02 (Fig 2C), avg. Spearman correlation coefficient for H4 = 0.55 ± 0.02, n = 31 ISCs] H2A [avg. Pearson correlation coefficient for H2A = 0.71 ± 0.02 (Fig 2C), avg. Spearman correlation coefficient for H2A = 0.74 ± 0.01, n = 50 ISCs] and H3T3A [avg. Pearson correlation coefficient for H3T3A = 0.49 ± 0.02 (Fig 4B), avg. Spearman correlation coefficient for H3T3A = 0.53 ± 0.02, n = 55 ISCs] analyses. There is a significant difference between the Pearson and Spearman correlation coefficients for H3 [avg. Pearson correlation coefficient for H3 = 0.44 ± 0.02 (Fig 1F), avg. Spearman correlation coefficient for H3 = 0.51 ± 0.02, n = 50 ISCs, **$P < 0.01$] and T2A-H3 co-expression [avg. Pearson correlation coefficient for T2A-H3 = 0.86 ± 0.01 (Fig 1F), avg. Spearman correlation coefficient for T2A-H3 = 0.79 ± 0.02, n = 19 ISCs, **$P < 0.01$]; however, the trend of correlation coefficients among different histones and labeling methods is the same when comparing results using these two analytic methods. When comparing the Spearman correlation coefficients of the H3 dataset to T2A-H3, H4, H2A, and H3T3A, the results remain consistent with that of the Pearson's analysis (shown in Figs 1F, 2C, and 4B), where H3 is significantly different from T2A-H3 and H2A (****$P < 0.0001$), but not significantly different from H4 or H3T3A (n.s.). Additionally, H2A is significantly different from H3T3A (****$P < 0.0001$), consistent with the Pearson's data shown in Fig 4B. Individual data points and mean values are shown. Error bars represent SEM. ****$P < 0.0001$, **$P < 0.01$, NS, not significant; unpaired t-test to compare two individual datasets to each other. Individual data values are shown in Dataset EV1.

Data information: n for individual ISCs. Scale bar in (A) and (B): 50 μm.

© 2023 The Authors EMBO reports 24: e56404 | 2023 25 of 24

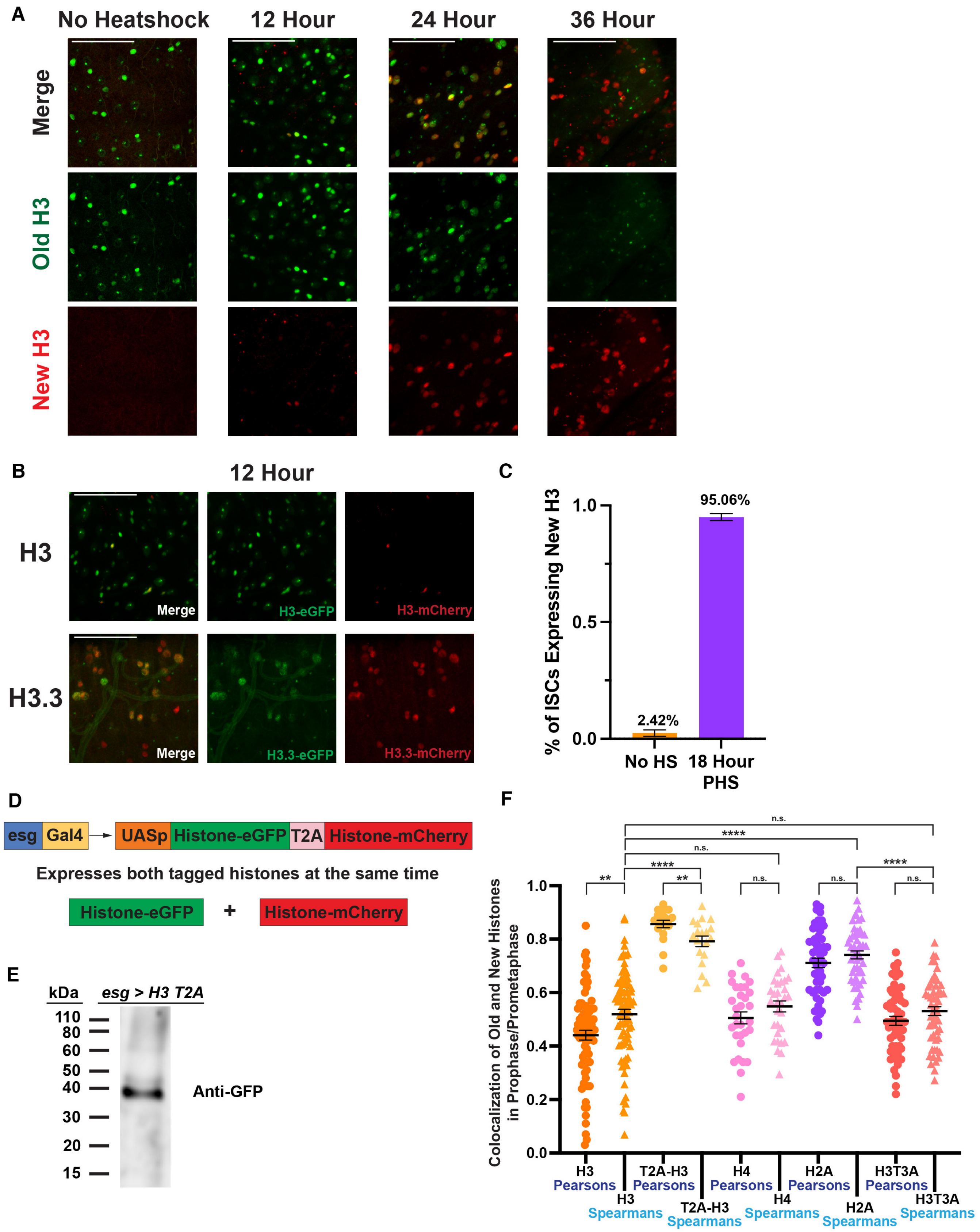

Figure EV1.

© 2023 The Authors

Figure EV2. Related to Fig 3: Old and new H3 expressed using the ISC-specific combination of drivers esg-Gal4, Su(H)-Gal80 shows similar expression patterns when compared to using the esg-Gal4 driver. Co-expressed eGFP- and mCherry-tagged H3 show symmetric inheritance in both Delta-asymmetric and Delta-symmetric pairs.

A A representative image showing expression of the dual-color histone H3 transgene using the *esg-Gal4* driver with heat-shock treatment and recovery, using *esg-Gal4 > UASp-FRT-H3-eGFP-FRT-H3-mCherry*. Both eGFP (Green)- and mCherry (Red)-tagged histones can be visualized. *Dl-nLacZ* expression (Gray) is used for ISC marker. Cells with positive EdU signal introduced by a pulse labeling (Magenta) can be detected and are specified by the magenta circle.

B A representative image showing expression of the dual-color histone H3 transgene using the combination of *esg-Gal4*, *Su(H)-Gal80 > UASp-FRT-H3-eGFP-FRT-H3-mCherry* with heat-shock treatment and recovery, which shows ISC-specific pattern. Both eGFP (Green)- and mCherry (Red)-tagged histones can be visualized. *Dl-nLacZ* expression (Gray) is used for ISC marker. Cells with positive EdU signal (Magenta) introduced by a pulse labeling can be detected and are specified by the magenta circle.

C Co-expression of eGFP- and mCherry-tagged H3 using the transgene shown in Fig EV1 (*esg-Gal4 > UASp-H3-eGFP-T2A-H3-mCherry-PolyA*) in a Delta-asymmetric pair of cells: eGFP- and mCherry-tagged H3 distribution in a postmitotic pair of cells with asymmetric Dl-nLacZ labeling, showing that H3-eGFP and H3-mCherry are equally inherited in the Delta-high cell and the Delta-low cell.

D Co-expression of eGFP- and mCherry-tagged H3 in a Delta-symmetric pair of cells: eGFP- and mCherry-tagged H3 distribution in a postmitotic pair of cells with symmetric Dl-nLacZ labeling, showing that H3-eGFP and H3-mCherry are equally inherited in the two cells.

E Quantification of H3-eGFP and H3-mCherry distribution in Delta-asymmetric pair of cells in the co-expressed H3 line (avg. $\log_2$ ratio for eGFP-H3 = 0.03 ± 0.08, avg. $\log_2$ ratio for mCherry-H3 = 0.01 ± 0.06, n = 15 pairs, N = 2 intestines), compared with old H3-eGFP and new H3-mCherry quantification from the *esg-Gal4* driven experiment in Fig 3C. Individual data points and mean values are shown. Error bars represent the standard error of the mean (SEM). ****$P < 0.0001$. An unpaired two-sample t-test to compare two individual datasets to each other. Individual data values are shown in Dataset EV2 and Table EV5.

F Quantification of H3-eGFP and H3-mCherry distribution in Delta-symmetric pair of cells in the co-expressed H3 line (avg. $\log_2$ ratio for GFP-H3 = 0.07 ± 0.08, avg. $\log_2$ ratio for mCherry-H3 = 0.00 ± 0.07, n = 11 pairs, N = 2 intestines), compared with old H3-eGFP and new H3-mCherry quantification using the *esg-Gal4* driver shown in Fig 3C. Individual data points and mean values are shown. Error bars represent the standard error of the mean (SEM). N.S.: not significant. Unpaired t-test to compare two individual datasets to each other. Individual data values are shown in Dataset EV2 and Table EV8. Between the two cells, cell 1 has the higher Dl-nLacZ level compared with cell 2 (Materials and Methods).

Data information: Scale bar in (A) and (B): 50 μm. Scale bar in (C) and (D): 5 μm; asterisk, ISC side.

© 2023 The Authors

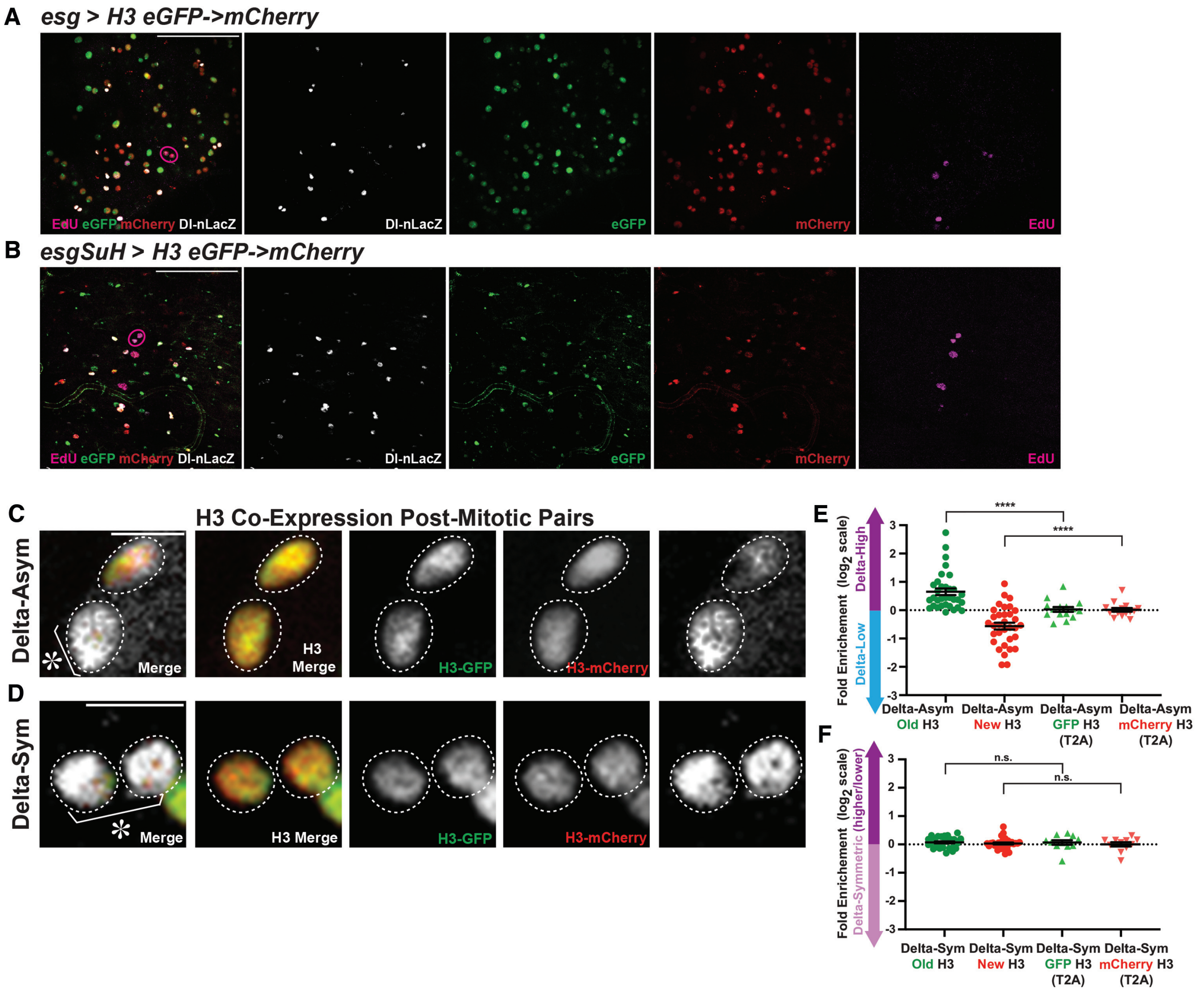

Figure EV2.

© 2023 The Authors

Figure EV3. Related to Figs 3, 5, and 6: Old and new histone H3 displays asymmetric inheritance pattern in pairs of cells when asymmetric versus symmetric Delta expression is identified using immunostaining with Delta antibody. Additionally, the internal driver marker for ISCs, YFP, shows distinct overlap with Dl-nLacZ, marking ISCs, and separable signals with Su(H)-LacZ, marking EBs.

A Old and new H3 distribution in a postmitotic pair of cells with punctate Delta antibody staining, showing that H3-eGFP (old) is asymmetrically inherited by the Delta-high cell, while H3-mCherry (new) is enriched in the Delta-low cell, using *esg-Gal4* > *UASp-FRT-H3-eGFP-FRT-H3-mCherry*.

B Old and new H3 distribution in a postmitotic pair of cells with punctate Delta antibody staining in both cells, showing that both H3-eGFP (old) and H3-mCherry (new) are symmetrically distributed between the two cells with symmetric Delta expression pattern.

C Comparisons of the quantification of old H3 in postmitotic pairs of cells using the Delta-nLacZ reporter line (data from Fig 3C), and the Delta antibody to identify pairs of cells with Delta-asymmetric or Delta-symmetric expression patterns. Quantification of H3-eGFP (old) in Delta-asymmetric pairs identified by the Delta antibody (avg. $\log_2$ ratio for old H3 = 0.71 ± 0.10, n = 41 pairs) is similar to that of Delta-asymmetric pairs identified by the Delta-nLacZ reporter (avg. $\log_2$ ratio for old H3 = 0.65 ± 0.12, n = 33 pairs, Fig 3C). Similarly, quantification of H3-eGFP (old) in Delta-symmetric pairs was similar between pairs identified by the Delta antibody (avg. $\log_2$ ratio for old H3 = −0.003 ± 0.09, n = 10 pairs) and pairs identified by the Delta-nLacZ reporter (avg. $\log_2$ ratio for old H3 = 0.07 ± 0.03, n = 30 pairs, Fig 3C).

D Old and new H2A distribution in a postmitotic pair of cells with asymmetric punctate Delta antibody staining, showing that H2A-eGFP (old) and H2A-mCherry (new) are symmetrically inherited by the two cells, using *esg-Gal4* > *UASp-FRT-H2A-eGFP-FRT-H2A-mCherry*.

E Old and new H2A distribution in a postmitotic pair of cells with symmetric punctate Delta antibody staining, showing that both H2A-eGFP (old) and H2A-mCherry (new) are symmetrically distributed between the two cells.

F Comparisons of the quantification of old H2A in postmitotic pairs of cells using the Delta-nLacZ reporter line (data from Fig 3K), or the Delta antibody. Quantification of H2A-eGFP (old) in Delta-asymmetric pairs identified by the Delta antibody (avg. $\log_2$ ratio for old H2A = 0.002 ± 0.08, n = 32 pairs) is similar to that of Delta-asymmetric pairs identified by the Delta-nLacZ reporter (avg. $\log_2$ ratio for old H2A = −0.03 ± 0.07, n = 30 pairs, Fig 3K). Similarly, quantification of H2A-eGFP (old) in Delta-symmetric pairs was similar between pairs identified by the Delta antibody (avg. $\log_2$ ratio for old H2A = 0.03 ± 0.06, n = 6 pairs) and pairs identified by the Delta-nLacZ reporter (avg. $\log_2$ ratio for old H2A = 0.08 ± 0.03, n = 30 pairs, Fig 3K).

G Comparison of YFP and Dl-nLacZ expression. YFP was driven by the ISC-specific driver, *esg-Gal4*, *Su(H)-Gal80*, *tub-Gal80^{ts}* at the Gal80^{ts} restrictive temperature (29°C). Significant overlap of YFP and Dl-nLacZ is observed, with both signals marking ISCs.

H Comparison of YFP and Su(H)-LacZ expression. YFP was driven by the ISC-specific driver, *esg-Gal4*, *Su(H)-Gal80*, *tub-Gal80^{ts}* at the Gal80^{ts} restrictive temperature (29°C). Separable signals of YFP, marking ISCs, and Su(H)-LacZ, marking EBs, are observed.

Data information: n for the number of postmitotic pairs. For (C) and (F), individual data points and mean values are shown. Error bars represent SEM. ****P < 0.0001, *P < 0.05; single-sample t-test (for normally distributed data) for comparing one dataset to a hypothesized mean of 0 ($\log_2$ value = 0 representing a 1:1 ratio), or Wilcoxon signed-rank test (for skewed data) for comparing one dataset to a hypothesized median of 0. Unpaired t-test to compare two individual datasets to each other. NS, not significant. Individual data values are shown in Dataset EV4. Scale bar in (A), (B), (D) and (E): 5 μm; Scale bar in (G) and (H): 50 μm; asterisk, ISC side.

© 2023 The Authors

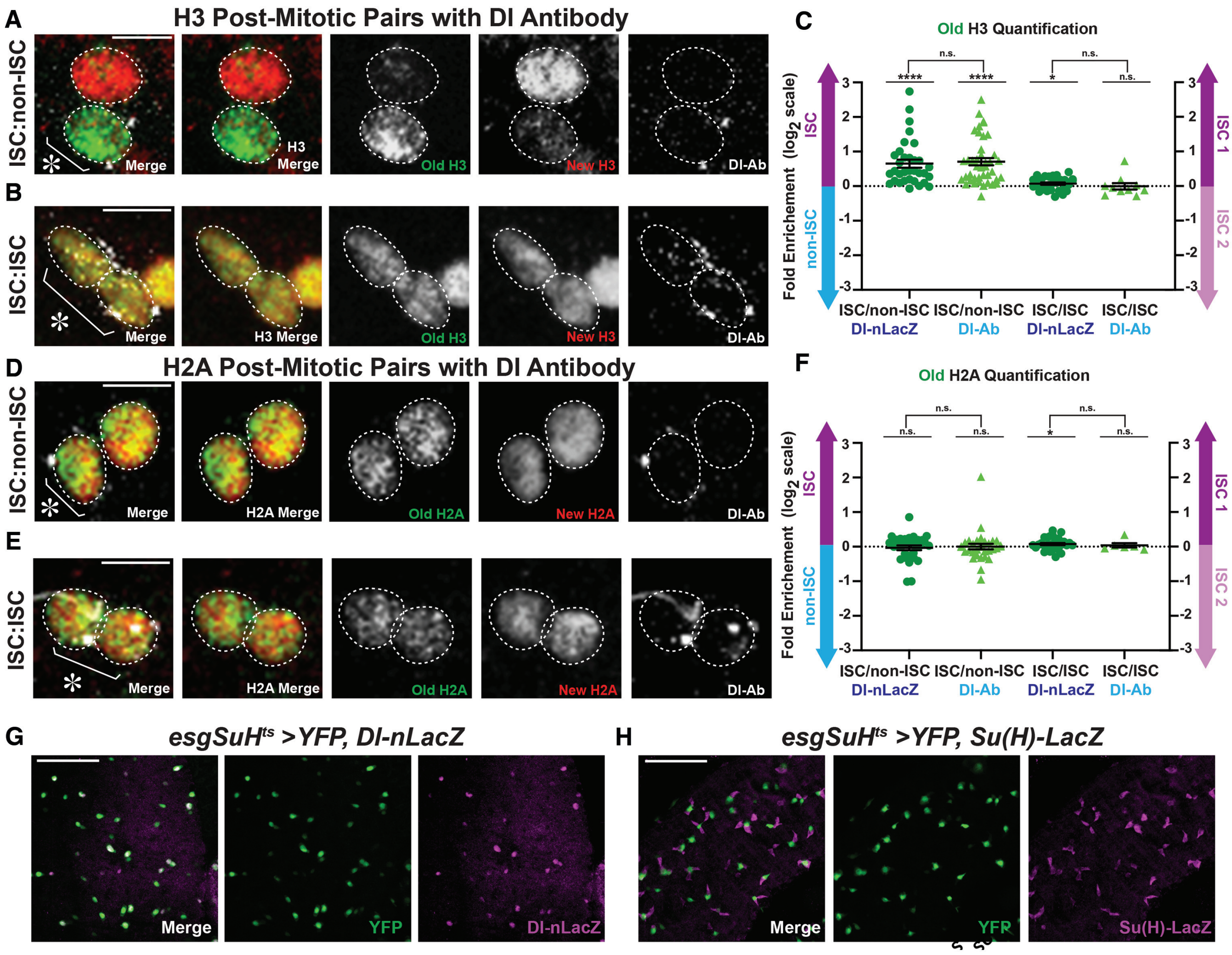

Figure EV3.

Figure EV4. Related to Fig 6. Single-cell RNA-seq using H3-expressing and H3T3A-expressing intestinal samples show compromised ISC differentiation in the H3T3A-expressing samples.

A, B Clusters were identified from a list of marker genes (Hung *et al*, 2020). The H3 and H3T3A samples are very similar in the average expression levels of these different clusters. Only H3T3A (after quality control) contained a cluster with no expression of any marker which uniquely identified the other clusters (unknown).

C We fitted a regression model to predict Delta levels in pseudotime (*x*-axis). Our outlier removal (outliers removed in Fig 6I) did not affect the significance level, but did increase the slope of the wild-type regression line by roughly twofold, which is a desirable quality.

D The regression model for klu levels is also similar to its counterpart after outlier removal (Fig 6J). Outliers did not change the direction of the slope or the significance level, but had a negative effect on the wild-type line, which has a small slope here.

E–H The exclusion of outliers [the Unknown class of the scType classifier (Ianevski *et al*, 2022)] is detailed. scType may be affected by differences in separability between Dl^+ and klu^+ populations in H3T3A. The outliers do not follow the same pattern in the H3 and H3T3A samples, but we chose an off-the-shelf solution for removal of outliers based on marker genes, to avoid biasing the result. We removed outliers shown in (E and F); compare (C) with Fig 6I. We removed outliers shown in (G and H); compare (D) with Fig 6J.

© 2023 The Authors

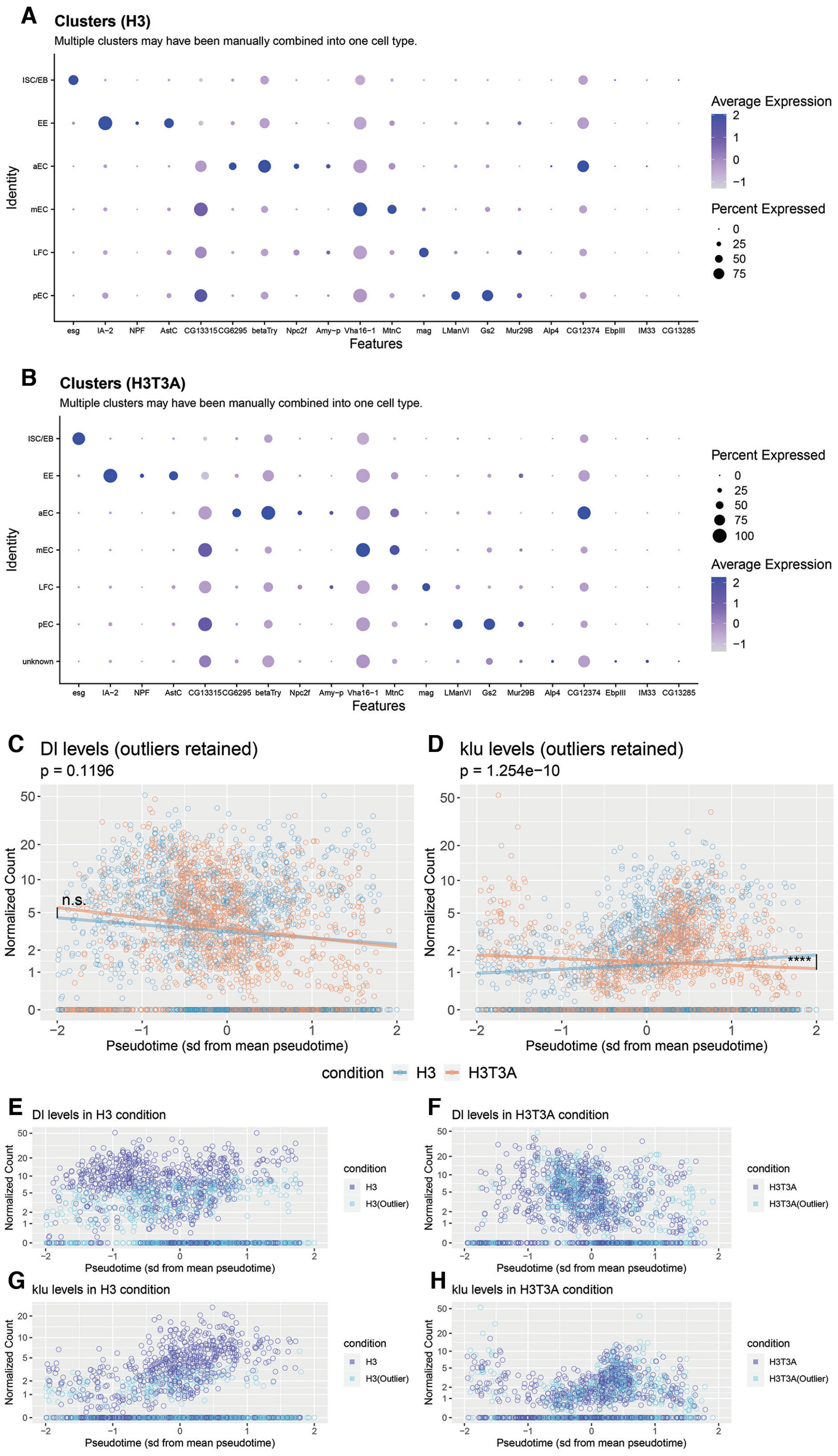
A
Clusters (H3)
Multiple clusters may have been manually combined into one cell type.
Identity
ISC/EB
EE
aEC
mEC
LFC
pEC
esg IA−2 NPF AstC CG13315 CG6295 betaTry Npc2f Amy−p Vha16−1 MtnC mag LManVI Gs2 Mur29B Alp4 CG12374 EbpIII IM33 CG13285
Features
Average Expression
Percent Expressed
B
Clusters (H3T3A)
Multiple clusters may have been manually combined into one cell type.
unknown
C
Dl levels (outliers retained)
p = 0.1196
n.s.
Normalized Count
Pseudotime (sd from mean pseudotime)
D
klu levels (outliers retained)
p = 1.254e−10
****
condition H3 H3T3A
E
Dl levels in H3 condition
H3(Outlier)
F
Dl levels in H3T3A condition
H3T3A(Outlier)
G
klu levels in H3 condition
H
klu levels in H3T3A condition

Figure EV4.

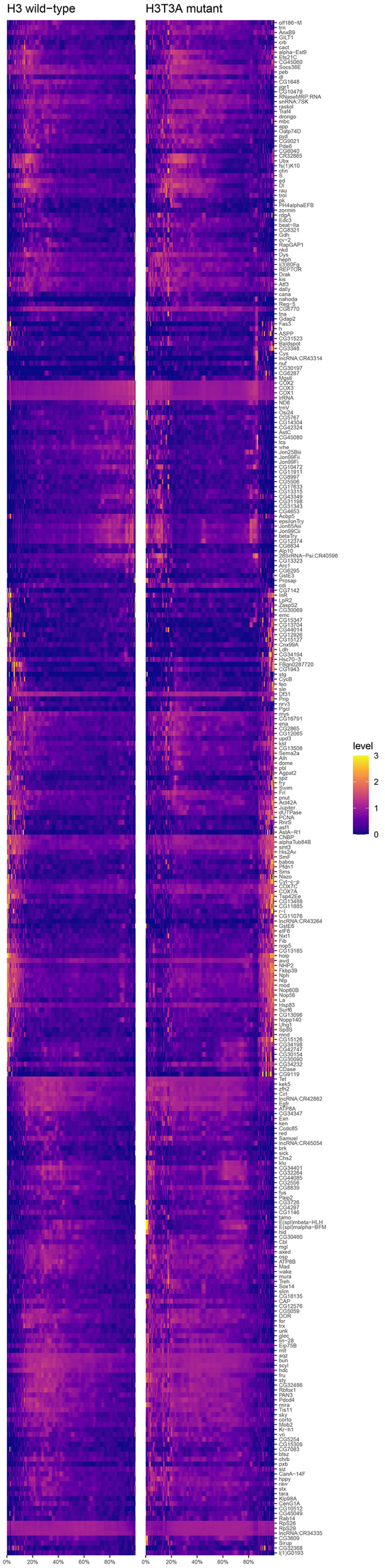

Figure EV5. Related to Fig 6. Single-cell RNA-seq data showing representative individual gene expression profiles in H3-expressing and H3T3A-expressing intestinal samples.

H3 and H3T3A pseudotime is shown as gene levels varying from left to right. Genes are selected for significance according to the tradeSeq nonlinear differential expression library (Van den Berge *et al*, 2020). Note that klu levels are low where Dl levels are high, and vice versa.

 © 2023 The Authors
