# Supplementary material for: Old and newly synthesized histones are asymmetrically distributed in Drosophila intestinal stem cell divisions
Source: EMBO Rep. 2023 May 31;24(7):e56404. doi: 10.15252/embr.202256404 (PMC10328082; doi:10.15252/embr.202256404)
Supplement: Supplementary file 14 — PDF+ [file EMBR-24-e56404-s007.pdf]

Report

TRANSPARENT PROCESS OPEN ACCESS

EMBO reports

Old and newly synthesized histones are asymmetrically distributed in *Drosophila* intestinal stem cell divisions

Emily H Zion[1], Daniel Ringwalt[1,†], Kristina Rinaldi[1,†], Elizabeth W Kahney[1], Yingying Li[1] & Xin Chen[1,2,*]

Abstract

We report that preexisting (old) and newly synthesized (new) histones H3 and H4 are asymmetrically partitioned during the division of *Drosophila* intestinal stem cells (ISCs). Furthermore, the inheritance patterns of old and new H3 and H4 in postmitotic cell pairs correlate with distinct expression patterns of Delta, an important cell fate gene. To understand the biological significance of this phenomenon, we expressed a mutant H3T3A to compromise asymmetric histone inheritance. Under this condition, we observe an increase in Delta-symmetric cell pairs and overpopulated ISC-like, Delta-positive cells. Single-cell RNA-seq assays further indicate that H3T3A expression compromises ISC differentiation. Together, our results indicate that asymmetric histone inheritance potentially contributes to establishing distinct cell identities in a somatic stem cell lineage, consistent with previous findings in *Drosophila* male germline stem cells.

Keywords asymmetric cell division; differentiation; epigenetic inheritance; histone; stem cells

Subject Categories Chromatin, Transcription, & Genomics; Stem Cells & Regenerative Medicine

DOI 10.15252/embr.202256404 | Received 31 October 2022 | Revised 30 April 2023 | Accepted 16 May 2023 | Published online 31 May 2023

EMBO Reports (2023) 24: e56404

Introduction

In multicellular organisms, asymmetric cell division (ACD) of adult stem cells serves as an important mechanism for tissue homeostasis and regeneration (Knoblich, 2008; Morrison & Spradling, 2008; Kahney *et al*, 2017; Venkei & Yamashita, 2018). Disruption of this precisely regulated cell division mode can result in the dysregulation of stem cells, leading to cancer or tissue degeneration (Clevers, 2005; Morrison & Kimble, 2006; Knoblich, 2010; Zion *et al*, 2020).

Epigenetic mechanisms enable different cell types within a multicellular organism to establish distinct cellular identities while carrying the identical genetic information (Allis & Jenuwein, 2016). Canonical histone proteins H3, H4, H2A, and H2B are synthesized and incorporated into DNA during replication as an octamer structure, forming the fundamental unit of chromatin, the nucleosome (Kornberg, 1974; Luger *et al*, 1997; Richmond & Davey, 2003). It is well known that nucleosomes and chromatin structure can affect cell fate decisions (Bannister & Kouzarides, 2011; Stillman, 2018; Kornberg & Lorch, 2020; Zhang *et al*, 2021); however, it remains largely unclear how epigenetic information is retained or altered during cell divisions to produce cells with different identities in multicellular organisms (Kouzarides, 2007; Young *et al*, 2010; Badeaux & Shi, 2013; Allis & Jenuwein, 2016; Ahmad & Henikoff, 2018; Yadav *et al*, 2018; Escobar *et al*, 2021).

Previous studies have shown that H3 and H4 histones are asymmetrically inherited during ACD of the *Drosophila* male germline stem cells (GSCs), where preexisting (old) histones are retained in the self-renewed stem cell, while newly synthesized (new) histones are enriched in the differentiating daughter cell (Tran *et al*, 2012; Wooten *et al*, 2019). In contrast, old and new H2A and H2B are inherited more symmetrically during ACD of male GSCs (Wooten *et al*, 2019). It is hypothesized that the old H3 and H4 histones retain an epigenetic memory that is inherited by the self-renewed stem cell, while the newly synthesized histones lacking this information can be used to establish a new gene expression program in the differentiating cell. Complementary to this hypothesis, previous studies have reported differences in post-translational modifications between preexisting and newly synthesized histones (Alabert *et al*, 2015; Lin *et al*, 2016). Additionally, it has been shown that nucleosomal density displays differences between old and new histone-enriched sister chromatids, with the old histone side having higher overall nucleosomes than the new histone side (Ranjan *et al*, 2022). Nucleosome density and position have profound impacts on many cellular processes by modulating DNA accessibility to different regulators, including pioneer factors, transcription factors, and cell cycle regulators (Jiang & Pugh, 2009; Bai &

1 Department of Biology, The Johns Hopkins University, Baltimore, MD, USA
2 Howard Hughes Medical Institute, Baltimore, MD, USA
*Corresponding author. Tel: +1 410 516 4576; Fax: +1 410 516 5213; E-mail: xchen32@jhu.edu
†These authors contributed equally to this work

© 2023 The Authors. Published under the terms of the CC BY 4.0 license.

Morozov, 2010; Zaret & Carroll, 2011; Small *et al*, 2014; Chereji *et al*, 2018; Lai *et al*, 2018). One functional readout of the inheritance of asymmetric chromatin statuses in GSC divisions is the asymmetric recruitment of the DNA replication component Cdc6, which allows asynchronous cell cycle progression in the resulting daughter cells (Ranjan *et al*, 2022). Furthermore, when asymmetric H3 segregation is disrupted, progenitor germline tumors and germ cell loss phenotypes are both detected, suggesting that this process is required for both stem cell maintenance and proper germline differentiation (Xie *et al*, 2015). Recently, a new study revealed that proper interactions between homologous chromosomes at a critical "stemness" *stat92E* gene locus depend on asymmetric H3 inheritance, without which *stat92E* gene expression becomes misregulated (Antel *et al*, 2022). The findings of asymmetric histone inheritance in *Drosophila* male GSCs set a precedent in studying epigenetic inheritance modes in multicellular organisms. The question remains, however, of whether this phenomenon is germ cell-specific or if it serves as a more general mechanism. It also remains unclear whether asymmetrically inherited histones aid in defining distinct cell fates at a single-cell level. Addressing these questions will not only greatly enhance our current understanding of how epigenetic inheritance modes dictate cell fates, but it will also help establish new methods to identify *bona fide* stem cells and asymmetrically dividing cells *in vivo*.

To investigate the generality of asymmetric histone inheritance, we use the *Drosophila* intestinal stem cells (ISCs) in the midgut as a model system (Micchelli & Perrimon, 2006; Ohlstein & Spradling, 2006). One feature of ISCs is that they can alternate between ACD, which produces a self-renewed ISC and a differentiating daughter of either an enteroblast (EB) or a pre-enteroendocrine (pre-ee) cell, and symmetric cell division (SCD), which results in two self-renewed ISCs (Fig 1A; O'Brien *et al*, 2011; de Navascues *et al*, 2012; Zeng & Hou, 2015; Martin *et al*, 2018). As the Notch (N) signaling pathway is critical for cellular differentiation in the ISC lineage, the expression of Delta can be used as an ISC-enriched cellular marker. We propose that the ISC lineage is a great system to study histone inheritance due to its well-characterized lineage, clearly distinguishable ISC-specific mitosis, and abundant ISCs *in vivo*. Indeed, previous studies have shown asymmetric inheritance of old versus new centromere-specific histone H3 variant CENP-A (i.e., CID in *Drosophila*) in the ISCs (Garcia Del Arco *et al*, 2018). Here, using this system we report that asymmetric canonical histone inheritance applies to this somatic stem cell lineage during ACD of ISCs and that misregulation of this process leads to midgut hyperplasia with ISC-like cells. Collectively, these results demonstrate that asymmetric histone inheritance could be a more general feature for asymmetric stem cell divisions. This study also offers insight into how histone inheritance could influence the establishment or maintenance of cell identities and how misinheritance could lead to diseases such as cancer.

Results

Differential distribution of old versus new H3 in prophase-to-prometaphase ISCs

To study histone distribution and inheritance patterns during ISC divisions, we optimized a dual-color histone labeling and tracking system in the ISCs (Fig 1B), similar to what has been previously used (Tran *et al*, 2012; Xie *et al*, 2015; Wooten *et al*, 2019). Here, the expression of labeled histones was driven by a cell type-specific *escargot-Gal4* (*esg-Gal4*) to turn on the *UAS-histone* transgene in the ISC lineage (Micchelli & Perrimon, 2006). After a heat-shock-induced switch from eGFP to mCherry-labeled histone expression, ISCs were allowed to undergo at least a complete round of DNA replication after an approximately 18-h recovery time (Fig 1C), shown by robust incorporation of new replication-dependent canonical histones genome-wide. This time frame was determined through a time course experiment, where the expression and incorporation of new H3-mCherry began around 12 h after heat shock. By 24 h, robust signals of both H3-eGFP and H3-mCherry could be detected.

Figure 1. Old and new histone H3 displays a differential distribution pattern in mitotic ISCs.

A A diagram of the ISC lineage. ISCs can asymmetrically divide into a self-renewed ISC and one of two differentiating cell types, an enteroblast (EB) or a pre-enteroendocrine cell (preEE). EBs can mature into enterocytes (ECs), and preEEs can mature into enteroendocrine cells (EEs). ISCs can also symmetrically divide, producing two ISCs. The precocious ISC division that leads to two non-ISCs, such as two EB cells, are not included in this diagram as neither daughter cell would be labeled with the ISC markers used.

B The dual-color labeling system with the *UASp-FRT-histone-eGFP-PolyA-FRT-histone-mCherry-PolyA* transgene is driven in the progenitor cells of the ISC lineage using a cell type- and stage-specific *escargot (esg)-Gal4* driver. Before heat shock, the transgene expresses eGFP-labeled histones. After heat-shock-induced DNA recombination, the transgene turns off expression of eGFP-labeled histones and turns on expression of mCherry-labeled histones.

C Experimental design: ISCs initially express eGFP-tagged histones. After heat shock, mCherry-tagged new histones are incorporated genome-wide during S phase. The distribution of old (eGFP-tagged) and new (mCherry-tagged) histones in early mitosis (prophase and prometaphase) and the segregation in late mitosis (anaphase and telophase) are investigated.

D Using the dual-color labeling system (*esg-Gal4* > *UASp-FRT-H3-eGFP-FRT-H3-mCherry*), old and new H3 distribution in prophase and prometaphase ISCs shows large, separable domains of H3-eGFP (old) and H3-mCherry (new). Note: all figure panels in this work are maximum intensity projection images to show signals from all Z-stacks.

E Using the *UASp-FRT-histone-eGFP-T2A-histone-mCherry-PolyA* transgene that co-expresses H3-eGFP and H3-mCherry, significant overlap between the two signals was detected in ISCs during prophase and prometaphase.

F Quantification of the colocalization between old and new H3, as well as between the co-expressed eGFP- and mCherry-tagged H3 in prophase and prometaphase ISCs. Pearson's correlation coefficients were measured, where 1 represents complete colocalization and 0 stands for no colocalization. Old and new H3 showed significantly less colocalization (Pearson's correlation coefficient for H3 = 0.44 ± 0.02, n = 81 ISCs) when compared to the co-expressed eGFP- and mCherry-tagged H3 (Pearson's correlation coefficient for H3 co-expressed = 0.86 ± 0.01, n = 19 ISCs). Individual data points and mean values are shown. Error bars represent SEM. ****P < 0.0001; unpaired t-test to compare two individual datasets to each other. Individual data values are shown in Dataset EV1.

Data information: n for individual ISCs. Scale bar in (D), (E): 5 μm.

© 2023 The Authors

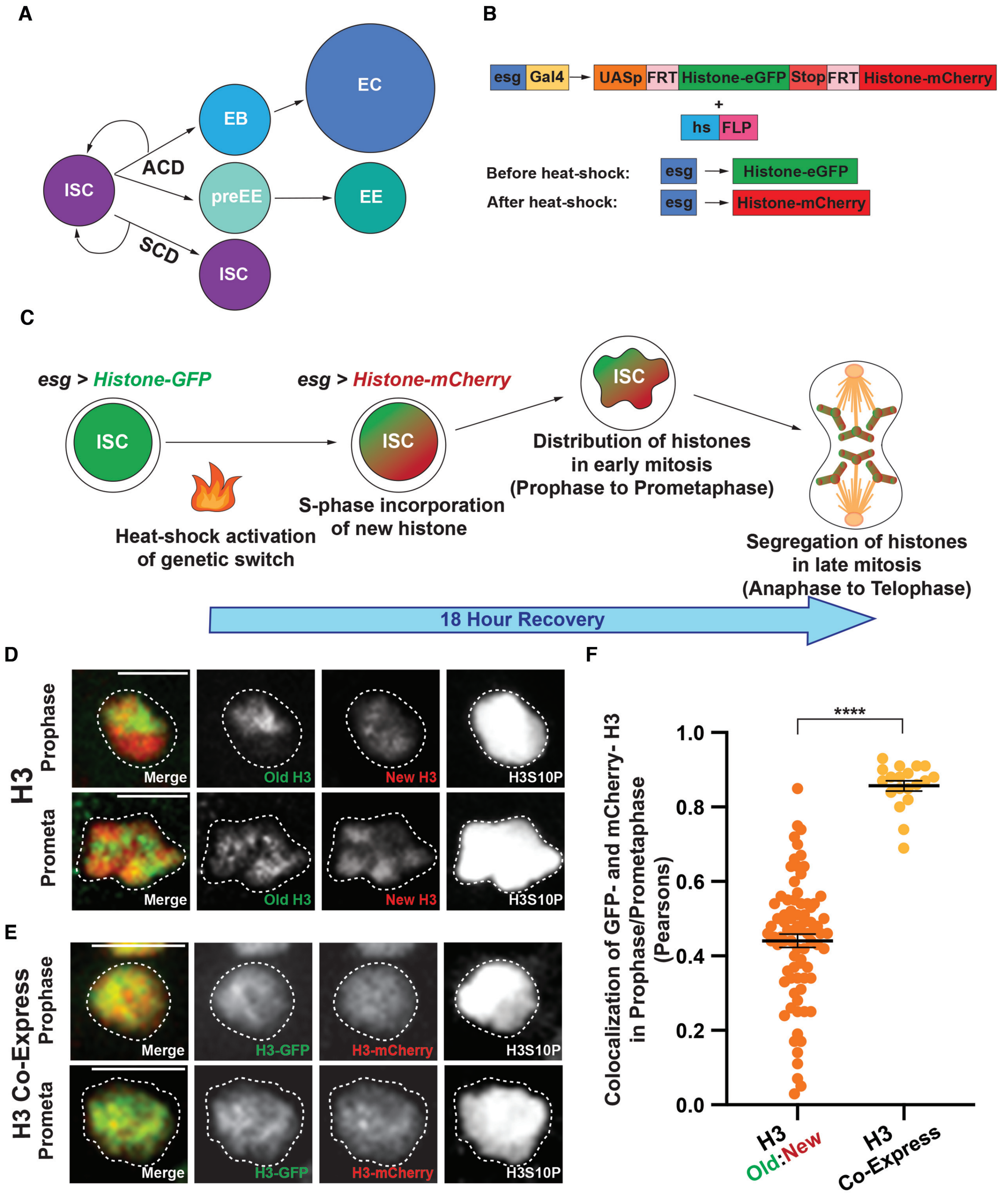
A
ISC
ACD
SCD
EB
EC
preEE
EE
ISC
B
esg
Gal4
UASp
FRT
Histone-eGFP
Stop
FRT
Histone-mCherry
+
hs
FLP
Before heat-shock:
esg
Histone-eGFP
After heat-shock:
esg
Histone-mCherry
C
esg > Histone-GFP
ISC
Heat-shock activation of genetic switch
esg > Histone-mCherry
ISC
S-phase incorporation of new histone
ISC
Distribution of histones in early mitosis (Prophase to Prometaphase)
Segregation of histones in late mitosis (Anaphase to Telophase)
18 Hour Recovery
D
H3
Prophase
Prometa
Merge
Old H3
New H3
H3S10P
E
H3 Co-Express
Prophase
Prometa
Merge
H3-GFP
H3-mCherry
H3S10P
F
Colocalization of GFP- and mCherry- H3 in Prophase/Prometaphase (Pearsons)
1.0
0.8
0.6
0.4
0.2
0.0
****
H3 Old:New
H3 Co-Express

Figure 1.

However, by 36 h old H3-eGFP signals became almost undetectable (Fig EV1A). This time lapse required for new H3 incorporation is consistent with the replication-dependent incorporation mode for canonical histones. Contrastingly, the incorporation of new histone variant H3.3-mCherry which is independent of DNA replication showed robust signals 12 h after heat shock (Fig EV1B). Quantification showed minimal flipped-out without heat shock but efficient flipped-out at 18 h after heat shock (Fig EV1C), which is the time point we performed most imaging-based analysis. These results are consistent with previous data using GFP-tagged H3 versus GFP-tagged H3.3 that show distinct incorporation modes between DNA replication-dependent incorporation of the canonical H3 and DNA replication-independent incorporation of the H3.3 histone variant in *Drosophila* (Ahmad & Henikoff, 2002b). The ISCs co-labeled with both eGFP (old histones) and mCherry (new histones) signals entered the subsequent mitosis, when sister chromatids are condensed and segregated equally into the daughter cells. The mitotic ISCs (Micchelli & Perrimon, 2006; Ohlstein & Spradling, 2006; Jiang *et al*, 2009; Chen *et al*, 2018) can be labeled using a mitotically enriched H3S10ph mark (phosphorylation at Serine 10 of H3; Hendz*el et al*, 1997). Using this mitotic mark, *esg* driver-labeled ISCs can be distinguished in this histone tracer assay. Here, we studied old versus new histone distribution patterns at different mitotic stages of ISCs.

First, we examined old versus new H3 distribution patterns in the mitotic ISCs at prophase and prometaphase. Interestingly, separable domains enriched with either old or new H3 could be readily visualized when chromosomes undergo condensation at prophase and prometaphase (Fig 1D). Notably, all images shown here and after are maximum intensity projections to display signals coming from all Z-stacks but not a particular slice to avoid any potential bias. Next, as a control for these results of old H3 versus new H3, we generated a fly line carrying a *UAS-H3-eGFP-T2A-H3-mCherry* transgene, in which *H3-eGFP* and *H3-mCherry* sequences have a *T2A* self-cleaving peptide sequence in between (Fig EV1D and E), so that they are expressed in an equal molar ratio simultaneously, when driven by the same *esg-Gal4* driver. We find that this control showed a high degree of overlap between H3-eGFP and H3-mCherry signals in prophase and prometaphase ISCs (Fig 1E). To quantify these imaging results, we measured Pearson's correlation coefficient between these two signals, as used previously (Kahney *et al*, 2021). Here, a zero 0.0 correlation coefficient indicates no colocalization, while the 1.0 correlation coefficient represents complete overlapping between the two signals. As shown in Fig 1F, old H3-eGFP and new H3-mCherry displayed the average correlation coefficient of 0.44, while co-expressed H3-eGFP and H3-mCherry had the average correlation coefficient of 0.86. Together, these results demonstrate that old and new H3 differentially distributes in the mitotic ISCs and that this pattern is due to their protein age difference rather than the distinct fluorescence tags they carry.

Different distribution and segregation patterns of H3, H4, and H2A in the mitotic ISCs

During DNA replication, old histone octamers must be disassembled and reincorporated into the duplicated sister chromatids (Xu & Zhu, 2010; Snedeker *et al*, 2017; Serra-Cardona & Zhang, 2018; Stewart-Morgan *et al*, 2020). It has been demonstrated that old H3 and H4 are reincorporated as a tetramer, while old H2A and H2B split into two dimers following their dissociation from DNA strands during replication (Jackson & Chalkley, 1981; Russev & Hancock, 1981; Jackson, 1988; Xu *et al*, 2010; Katan-Khaykovich & Struhl, 2011). To investigate the molecular specificity of asymmetric histone inheritance, we went ahead and examined the old versus new histone distribution patterns of H4 and H2A. Interestingly, separable domains of old and new H4 could also be readily visualized in ISCs at prophase and prometaphase (Fig 2A), similar to the old and new H3 patterns at the comparable stages (Fig 1D). Contrastingly, this separation was not as evident for old and new H2A, which displayed a more overlapping pattern in prophase and prometaphase ISCs (Fig 2B). We then used Pearson's correlation analysis to quantify the colocalization between old and new histones. The average correlation coefficient for old and new H4 was 0.51, while the average coefficient for old and new H2A was 0.71, which is significantly different from that of both H3 and H4 (Fig 2C). Notably, there is a detectable difference between H3 and H4, shown as significantly different distribution patterns between old and new H3 and H4 in prophase and prometaphase ISCs ($P < 0.05$; Fig 2C). Similar results were obtained using the Spearman's correlation coefficient to determine the colocalization of old versus new histones (Fig EV1F). On the contrary, the H3-eGFP and H3-mCherry expressed from the *UAS-H3-eGFP-T2A-H3-mCherry* transgene control still showed the highest degree of overlap in prophase and prometaphase ISCs, which is more than any of the canonical histones (Fig EV1F), confirming that the separation patterns observed between old and new histones are due to their molecular identity and age differences.

Next, we examined histone segregation patterns by quantifying the entire protein amount associated with each set of the sister chromatids followed by computing the ratios between them, using the sum of slices method in anaphase and telophase ISCs, similar to the analyses reported previously [see Materials and Methods and (Ranjan *et al*, 2019, 2022)]. We could detect asymmetric segregation patterns for H3 (Fig 2D) and, to a lesser extent, for H4 (Fig 2E). In contrast, H2A displayed a more symmetric segregation pattern in anaphase and telophase ISCs (Fig 2F). Based on the quantification of old histones similar to what was performed previously (Xie *et al*, 2015), old H3, and to a lesser degree old H4, tended to be enriched toward one set of sister chromatids, whereas old H2A distributed more equally between the two sets of sister chromatids (Fig 2G). Using thresholds to classify the degree of asymmetry, approximately 59.4% of the mitotic ISCs showed a high degree of H3 asymmetry (> 1.22-fold difference), 15.6% showed a medium level of H3 asymmetry (1.16- 1.22-fold difference), and 25.0% demonstrated a symmetric H3 pattern [≤ 1.15-fold difference, Fig 2H, see Materials and Methods and (Wooten *et al*, 2019)]. Notably, an ISC division could be either ACD or SCD, using different cellular markers or criteria (O'Brien *et al*, 2011; de Navascues *et al*, 2012; Goulas *et al*, 2012; Tian & Jiang, 2014; Jin *et al*, 2017). Interestingly, these ratios of asymmetric versus symmetric H3 segregation patterns are largely consistent with other published results measuring the percentages of ACD versus SCD of ISCs. Additionally, in anaphase or telophase ISCs these ratios were 20% for a high degree of asymmetry, 35% for a medium level of asymmetry, and 45% for a symmetric pattern for old H4 (Fig 2H). Contrastingly, for old H2A 90% of all quantified mitotic ISCs showed a symmetric segregation

 © 2023 The Authors

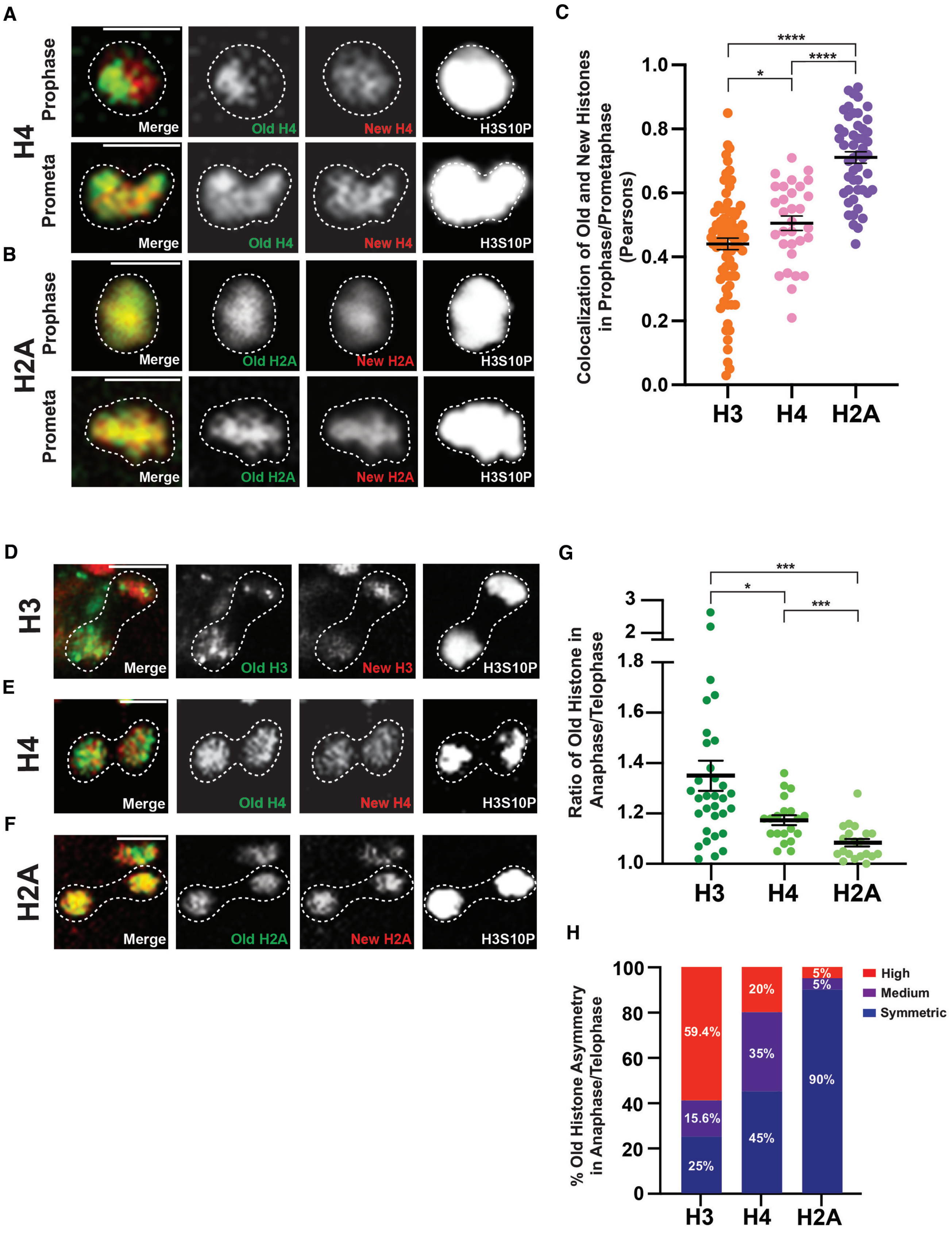

A
H4
Prophase
Prometa
Merge
Old H4
New H4
H3S10P
B
H2A
Old H2A
New H2A
C
Colocalization of Old and New Histones in Prophase/Prometaphase (Pearsons)
1.0
0.8
0.6
0.4
0.2
0.0
****
*
H3
H4
H2A
D
H3
Old H3
New H3
E
F
G
Ratio of Old Histone in Anaphase/Telophase
3
2
1.8
1.6
1.4
1.2
***
H
% Old Histone Asymmetry in Anaphase/Telophase
100
80
60
40
20
0
High
Medium
Symmetric
59.4%
15.6%
25%
20%
35%
45%
5%
90%

Figure 2.

Figure 2. Different distribution and segregation patterns of H3, H4, and H2A during ISC division.

A Old and new H4 distribution in prophase and prometaphase ISCs (*esg-Gal4 > UASp-FRT-H4-eGFP-FRT-H4-mCherry*). Separable domains of H4-eGFP (old) and H4-mCherry (new) were observed, similar to the H3 patterns shown in Fig 1D.

B Old and new H2A distribution in prophase and prometaphase ISCs (*esg-Gal4 > UASp-FRT-H2A-eGFP-FRT-H2A-mCherry*), showing largely overlapping signals between H2A-eGFP (old) and H2A-mCherry (new).

C Quantification of colocalization between old (eGFP) and new (mCherry) H3, H4, and H2A in prophase and prometaphase ISCs. Pearson's correlation coefficients are measured, where 1 represents complete colocalization and 0 stands for no colocalization. Old and new H4 showed a similar, but slightly more colocalized result when compared to H3 (Pearson's correlation coefficient for H4 = 0.51 ± 0.02, n = 31 ISCs). Old and new H2A showed a significantly higher colocalization when compared to H3 and H4 (Pearson's correlation coefficient for H2A = 0.71 ± 0.02, n = 50 ISCs). Individual data points and mean values are shown. Error bars represent SEM. ****$P < 0.0001$, *$P < 0.05$; unpaired t-test to compare two individual datasets to each other. Individual data values are shown in Dataset EV1.

D Late-stage mitotic ISC showing asymmetric segregation of H3 between the two sets of sister chromatids at the opposite poles of the ISC.

E Late-stage mitotic ISC showing less asymmetric segregation of H4, compared with H3, between the two sets of sister chromatids at the opposite poles of the ISC.

F Late-stage mitotic ISC showing symmetric segregation of H2A between the two sets of sister chromatids at the opposite poles of the ISC.

G Quantification of old H3, H4, and H2A distribution between the two sets of sister chromatids in anaphase and telophase ISCs. Old H3 is asymmetrically segregated between the two sets of sister chromatids (ratio = 1.35 ± 0.06, n = 32 ISCs). Old H4 is asymmetrically segregated, to a less extent compared with H3, between the two sets of sister chromatids (ratio = 1.17 ± 0.02, n = 20 ISCs) whereas H2A is more symmetrically segregated (ratio = 1.08 ± 0.02, n = 20 ISCs). Old histone signals were primarily used here, due to potential complications using new histone signals that have been discussed in this work and previously (Xie *et al*, 2015). Individual data points and mean values are shown. Error bars represent SEM. ***$P < 0.001$, *$P < 0.05$; unpaired t-test to compare two individual datasets to each other. Individual data values are shown in Table EV1.

H Percentage of different categories of old histone asymmetry for H3 (n = 32 ISCs), H4 (n = 20 ISCs), and H2A (n = 20 ISCs), where the three categories are symmetric (≤ 1.15-fold), medium asymmetric (1.16- to 1.22-fold), and high asymmetric (> 1.22-fold). The H2A dataset is used to define the symmetric range, with an average (avg) of 1.08 and standard deviation (std) of 0.07. Therefore, avg + 1*std = 1.15 defines the medium asymmetric range and avg + 2*std = 1.22 defines the high asymmetric range, similar to what was used before to define these ranges (Wooten *et al*, 2019).

Data information: n for individual ISCs. Scale bar in (A), (B), (D), (E), and (F): 5 μm.

pattern, while only 5% displayed a medium level of asymmetry and 5% displayed a high level of asymmetry (Fig 2H). Together, these results further confirm the molecular specificity of asymmetric histone inheritance with the highest degree of asymmetry for H3 and the lowest degree of asymmetry for H2A.

The detectable difference between H3 and H4 could be due to the fact that in higher eukaryotes, including *Drosophila*, H4 partners with H3 and the H3 histone variants, such as H3.3 and the centromere-specific CENP-A. Both H3.3 and CENP-A are not incorporated restrictively in a replication-dependent manner (Szenker *et al*, 2011). For example, the incorporation of H3.3 could be transcription-dependent (Ahmad & Henikoff, 2002a, 2002b; Tagami *et al*, 2004) or telomere region-enriched (Goldberg *et al*, 2010; Lewis *et al*, 2010). The deposition of CENP-A could be cell type-specific but is also independent of DNA replication (Schuh *et al*, 2007; Mellone *et al*, 2011; Dunleavy *et al*, 2012; Garcia Del Arco *et al*, 2018). Even during replication, old H3-H4 remain as tetramers whereas old H3.3-H4 tetramers tend to split into dimers (Xu *et al*, 2010), which would affect their reincorporation and the overall inheritance patterns. For CENP-A, it has been shown that old CENP-A is preferentially retained by ISCs in ACD (Garcia Del Arco *et al*, 2018). The transcription-dependent H3.3-H4 deposition, the possibly more symmetric reincorporation of H3.3-H4 during replication, and the incorporation timing and mode of CENP-A-H4 could all contribute to this detectable difference between H3 and H4. Taking together all mitotic ISC results (Figs 1 and 2), old and new H3 and H4 are differentially incorporated onto sister chromatids prior to mitosis, while old and new H2A are more uniformly incorporated. Additionally, old H3 and some of the old H4 are also asymmetrically inherited during ISC divisions.

Asymmetric old and new H3 and H4 patterns are associated with distinct Delta expression patterns

An ISC division could lead to two daughter cells with either distinct or similar cell fates, depending on its division mode of being either ACD or SCD. To visualize histone patterns after mitosis, we next examined eGFP-labeled old histone versus mCherry-labeled new histone in postmitotic pair of cells. ISCs are normally evenly distributed and spaced out in the fly midgut (Micchelli & Perrimon, 2006; Ohlstein & Spradling, 2006). Using a Delta-nuclear lacZ (Dl-nLacZ) reporter which can suggest ISC fate (Jiang *et al*, 2009; Beebe *et al*, 2010; Zeng *et al*, 2010) in combination with the dual-color histone H3 transgene driven by the *esg-Gal4* driver (Fig EV2A), we found that the total amount of old and new H3, quantified using the sum of slices method as described above, displayed an asymmetric pattern in the postmitotic pair of cells containing different Dl-nLacZ levels, with one Delta-high cell and another Delta-low cell (Fig 3A). Interestingly, more Dl-nLacZ signal was detected in the old H3-eGFP-enriched cell than in the new H3-mCherry-enriched cell. In contrast, old and new H3 displayed a more symmetric pattern in the postmitotic pair of cells containing comparable Dl-nLacZ levels (Fig 3B). Quantification of these results showed significant enrichment of old H3 in the Delta-high cell and new H3 enrichment in the Delta-low cell. Meanwhile, when two cells with comparable levels of Dl-nLacZ were examined, they carried more symmetric old and new H3 (Fig 3C). As a control, the H3-eGFP and H3-mCherry co-expressed from the *UAS-H3-eGFP-T2A-H3-mCherry* transgene did not display any polarized distribution in the two cells with either asymmetric Delta expression (Figs EV2C and 2E) or symmetric Delta expression (Figs EV2D and 2F). To better display the ratios of both old and new histones simultaneously, we used a two-dimensional plot to display both ratios from the same postmitotic pair of cells. As illustrated in Fig 3D, each of the four quadrants indicates a particular trend of old and new histone distribution patterns. Using this display, old and new H3 in Delta-symmetric pairs were largely clustered around the origin ($log_2 = 0$), consistent with their mainly symmetric distribution patterns using one-dimensional plots (Fig 1C). Contrastingly, old and new H3 in the postmitotic pair of cells with asymmetric Delta expression was mainly distributed in quadrant IV, where the ISC is enriched with old but depleted of

© 2023 The Authors

new H3; a small percentage of these pairs were in quadrant I, where the Delta-high cell is enriched with both old and new H3, which could reflect overall higher H3 levels in the Delta-high cell compared with the Delta-low cell (Fig 3D). A similar pattern of the overall H3 asymmetry was recently reported in asymmetrically dividing *Drosophila* male GSCs with a higher level of H3 in the stem cell (Ranjan *et al*, 2022).

Interestingly, old and new H4 also displayed an asymmetric inheritance pattern in the postmitotic pair of cells with asymmetric Delta expression (Fig 3E). Similar to H3, the asymmetric inheritance pattern of old versus new H4 was specific to Delta-asymmetric pairs, as the pairs with comparable Dl-nLacZ levels showed a more symmetric distribution of old versus new H4 (Fig 3F). Consistent with the H3 results, quantification of these results demonstrated that old H4 was preferentially inherited by the Delta-high cell, while new H4 was enriched in the Delta-low cell. In contrast, both old and new H4 was distributed almost equally between the two cells with symmetric Delta expression (Fig 3G). Notably, the ratios reflecting the degree of asymmetry for old and new H4 (~1.37-fold for old H4 and ~1.41-fold for new H4) are less than that of old and new H3 (~1.57-fold for old H3 and ~1.47-fold for new H3) in the postmitotic pair of cells with asymmetric Delta expression (Fig 3G versus C). However, all of these ratios are significantly different from a symmetrical ratio (1:1) and the ratios from Delta-symmetric pairs (see *P*-values in Fig 3G). Consistently, old and new H4 in postmitotic pairs showed similar distribution patterns like H3 using the two-dimensional plots (Fig 3H), with the H4 group mainly distributed in quadrant IV. Notably, the H4/H3 asymmetries in postmitotic pair of cells with asymmetric Delta expression are more pronounced than those detected in anaphase and telophase ISCs. This could be due to biological and/or technical reasons: Biologically, distinct cellular events in the postmitotic daughter cells could affect the histone distribution patterns after mitosis. For example, in the *Drosophila* male GSC system, the differentiating daughter cell enters the subsequent S phase prior to the self-renewed GSC, which could result in accelerated increase of new histones in the differentiating daughter cell (Ranjan *et al*, 2022). Technically, as discussed above, ISCs can alternate between ACD and SCD division modes. The Dl-nLacZ marker can distinguish these two modes in the postmitotic pair of cells so that we could quantify these two patterns separately. However, we

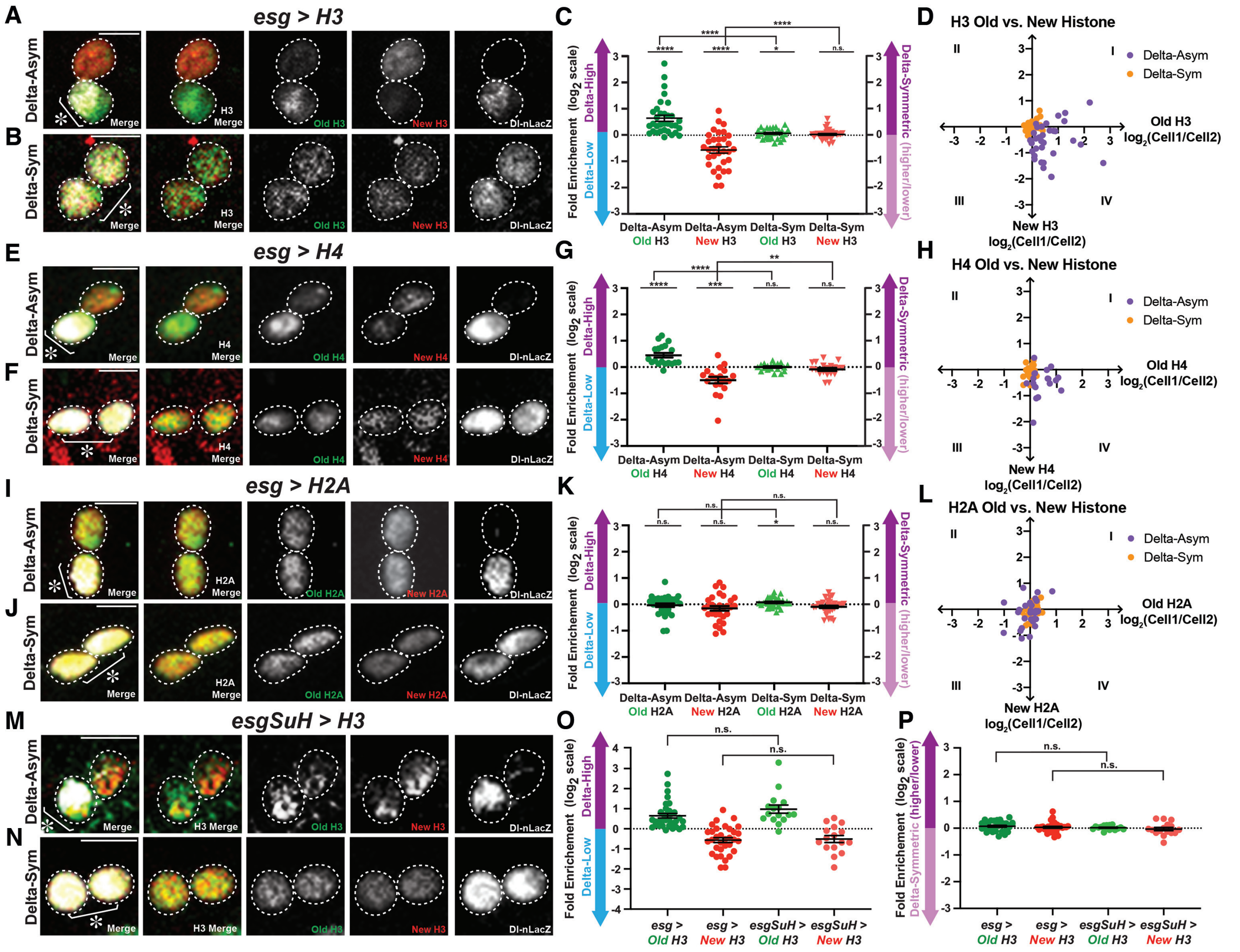

Figure 3.

Figure 3. Old and new H3, H4, and H2A inheritance patterns in Delta-asymmetric and Delta-symmetric postmitotic pairs.

A Old and new H3 distribution in a postmitotic pair of cells with asymmetric Dl-nLacZ labeling, showing that H3-eGFP (old) is asymmetrically inherited by the Delta-high cell, while H3-mCherry (new) is enriched in the Delta-low cell (*esg-Gal4 > UASp-FRT-H3-eGFP-FRT-H3-mCherry*).

B Old and new H3 distribution in a postmitotic pair of cells with symmetric Dl-nLacZ labeling, showing that both H3-eGFP (old) and H3-mCherry (new) are more symmetrically distributed between the two cells.

C Quantification of H3-eGFP (old) and H3-mCherry (new) distribution in Delta-asymmetric pairs (avg. $\log_2$ ratios for old H3 = 0.65 ± 0.12, avg. $\log_2$ ratios for new H3 = −0.56 ± 0.12, n = 33 pairs, N = 4 intestines) and Delta-symmetric pairs (avg. $\log_2$ ratio for old H3 = 0.07 ± 0.03, avg. $\log_2$ ratio for new H3 = 0.03 ± 0.03, n = 30 pairs, N = 4 intestines). Individual data points and mean values are shown. Error bars represent the standard error of the mean (SEM). ****$P < 0.0001$, *$P < 0.05$, n.s.: not significant; single-sample t-test (for normally distributed data) for comparing one dataset to a hypothesized mean of 0 ($\log_2$ value representing a 1:1 ratio), or Wilcoxon signed-rank test (for skewed data) for comparing one dataset to a hypothesized median of 0 ($\log_2$ value representing a 1:1 ratio). Unpaired t-test to compare two individual datasets to each other. Individual data values are shown in Dataset EV2. Between the two cells, cell 1 has a higher Dl-nLacZ level than cell 2 (Materials and Methods).

D 2D plot of corresponding old-H3 and new-H3 ratios ($\log_2$ values from Fig 3C) for each l pair of cells. A majority of Delta-asymmetric pairs fall in quadrant IV, indicating a higher inheritance of old H3 and a lower inheritance of new H3 by the Delta-high cell, while most Delta-symmetric pairs are clustered around the origin, indicating equal inheritance of old and new H3 by the two cells.

E Old and new H4 distribution in a postmitotic pair of cells with asymmetric Dl-nLacZ labeling, showing that H4-eGFP (old) is asymmetrically inherited by the Delta-high cell, while H4-mCherry (new) is enriched toward the Delta-low cell (*esg-Gal4 > UASp-FRT-H4-eGFP-FRT-H4-mCherry*).

F Old and new H4 distribution in a postmitotic pair with symmetric Dl-nLacZ labeling, showing that H4-eGFP (old) and H4-mCherry (new) are symmetrically distributed between the two cells.

G Quantification of H4-eGFP (old) and H4-mCherry (new) distribution in Delta-asymmetric pairs (avg. $\log_2$ ratio for old H4 = 0.45 ± 0.09, avg. $\log_2$ ratio for new H4 = −0.50 ± 0.12, n = 19 pairs, N = 2 intestines) and Delta-symmetric pairs (avg. $\log_2$ ratio for old H4 = 0.00 ± 0.03, avg. $\log_2$ ratio for new H4 = −0.09 ± 0.06, n = 19 pairs, N = 2 intestines). Individual data points and mean values are shown. Error bars represent SEM. ****$P < 0.0001$, ***$P < 0.001$, **$P < 0.01$, n.s., not significant; single-sample t-test (for normally distributed data) for comparing one dataset to a hypothesized mean of 0 ($\log_2$ value representing a 1:1 ratio), or Wilcoxon signed-rank test (for skewed data) for comparing one dataset to a hypothesized median of 0 ($\log_2$ value representing a 1:1 ratio). Unpaired t-test to compare two individual datasets to each other. Individual data values are shown in Dataset EV2.

H 2D plot of corresponding old-H4 and new-H4 ratios ($\log_2$ values from Fig 3G) for each pair of cells. A majority of Delta-asymmetric pairs fall in quadrant IV, indicating a higher inheritance of old H4 and a lower inheritance of new H3 by the Delta-high cell, while most Delta-symmetric pairs are clustered around the origin, indicating equal inheritance of old and new H3 by the two cells.

I Old and new H2A distribution in a postmitotic pair of cells with asymmetric Dl-nLacZ labeling, showing that H2A-eGFP (old) and H2A-mCherry (new) are symmetrically inherited by the two cells (*esg-Gal4 > UASp-FRT-H2A-eGFP-FRT-H2A-mCherry*).

J Old and new H2A distribution in a postmitotic pair of cells with symmetric Dl-nLacZ labeling, showing that H2A-eGFP (old) and H2A-mCherry (new) are symmetrically distributed between the two cells.

K Quantification of H2A-eGFP (old) and H2A-mCherry (new) distribution in Delta-asymmetric pairs (avg. $\log_2$ ratio for old H2A = −0.03 ± 0.07, avg. $\log_2$ ratio for new H2A = −0.15 ± 0.09, n = 30 pairs, N = 2 intestines) and Delta-symmetric pairs (avg. $\log_2$ ratio for old H2A = 0.08 ± 0.03, avg. $\log_2$ ratio for new H3 = −0.09 ± 0.05, n = 30 pairs, N = 2 intestines). Individual data points and mean values are shown. Error bars represent SEM. *$P < 0.05$, n.s.: not significant; single-sample t-test (for normally distributed data) for comparing one dataset to a hypothesized mean of 0 ($\log_2$ value representing a 1:1 ratio), or Wilcoxon signed-rank test (for skewed data) for comparing one dataset to a hypothesized median of 0 ($\log_2$ value representing a 1:1 ratio). Unpaired t-test to compare two individual datasets to each other. Individual data values are shown in Dataset EV2.

L 2D plot of corresponding old-H2A and new-H2A ratios ($\log_2$ values from Fig 3K) for each pair of cells. Both Delta-asymmetric and Delta-symmetric pairs are clustered around the origin without preferred quadrant, indicating equal inheritance of old and new H2A by the two cells.

M The ISC-specific combination of drivers *esg-Gal4*, *Su(H)-Gal80* drive H3 transgene [*esg-Gal4*, *Su(H)-Gal80 > UASp-FRT-H3-eGFP-FRT-H3-mCherry*] with heat-shock treatment and recovery (see Materials and Methods) in a Delta-asymmetric pair of cells. Old and new H3 distribution in a postmitotic pair of cells with asymmetric Dl-nLacZ labeling, showing that H3-eGFP (old) is asymmetrically inherited by the Delta-high cell, while H3-mCherry (new) is enriched in the Delta-low cell.

N ISC-specific drivers express the H3 transgene in a Delta-symmetric pair of cells. Old and new H3 distribution in a postmitotic pair of cells with symmetric Dl-nLacZ labeling, showing that both H3-eGFP (old) and H3-mCherry (new) are more symmetrically distributed between the two cells.

O Quantification of H3-eGFP (old) and H3-mCherry (new) distribution in Delta-asymmetric pair of cells driven by the ISC-specific combination of drivers *esg-Gal4*, *Su(H)-Gal80* (avg. $\log_2$ ratio for old H3 = 0.98 ± 0.20, avg. $\log_2$ ratio for new H3 = −0.51 ± 0.17, n = 16 pairs, N = 3 intestines), compared with H3-eGFP and H3-mCherry ratios using the *esg-Gal4* driver only, shown in Fig 3C. Individual data points and mean values are shown. Error bars represent the standard error of the mean (SEM). N.S.: not significant. Unpaired t-test to compare two individual datasets to each other. Individual data values are shown in Dataset EV2 and Table EV5.

P Quantification of H3-eGFP (old) and H3-mCherry (new) distribution in Delta-symmetric pair of cells (avg. $\log_2$ ratio for old H3 = 0.02 ± 0.02, avg. $\log_2$ ratio for new H3 = −0.03 ± 0.06, n = 16 pairs, N = 3 intestines), compared with H3-eGFP and H3-mCherry ratios using the *esg-Gal4* driver only, shown in Fig 3C. Individual data points and mean values are shown. Error bars represent the standard error of the mean (SEM). N.S.: not significant. Unpaired t-test to compare two individual datasets to each other. Individual data values are shown in Dataset EV2. Between the two cells, cell 1 has the higher Dl-nLacZ level compared with cell 2 (Materials and Methods).

Data information: n for the number of postmitotic pairs; N for the number of intestines. Scale bar in (A), (B), (E), (F), (I), (J), (M), and (N): 5 μm; asterisk, ISC side.

do not have a reliable cellular marker that can distinguish these two modes for the mitotic ISCs. Therefore, all mitotic ISCs are quantified as one group, containing both asymmetrically and symmetrically dividing ISCs.

Next, we examined the inheritance mode of old versus new H2A as a representative for the canonical histone dimer. Different from H3 and H4, old and new H2A showed a symmetric inheritance pattern in all postmitotic daughter cells of ISCs, regardless of whether they were Delta-asymmetric (Fig 3I) or Delta-symmetric pairs (Fig 3J). Quantification for old and new H2A showed almost no significant difference from a symmetrical pattern (a 1:1 ratio) for all postmitotic daughter cells of ISCs, and there was no significant difference between the two Delta expression patterns (see P-values in Fig 3K). Consistently, using the two-dimensional plot presentation, old and new H2A showed very clustered distribution around the origin for Delta-symmetric pairs (Fig 3L), while old and new H2A in the postmitotic pairs of cells expressing Delta asymmetrically were less clustered but showed no preference in any particular quadrant (Fig 3L). Together, these two-dimensional plots showed consistent results with the one-dimensional plot but provided more

© 2023 The Authors

information on the correlation between old and new histones in any given postmitotic pair of cells.

Furthermore, to circumvent any ambiguity calling two adjacent cells "daughters" derived from one ISC division, we combined the *esg-Gal4* activator with the Gal80 suppressor driven by *Suppressor of Hairless* [*Su(H)-Gal80*, (Loza-Coll *et al*, 2014; Wang *et al*, 2015)], in order to achieve a tight ISC-specific expression of the histone transgene (Fig EV2B). With the restrictive histone transgene expression using the *esg-Gal4 Su(H)Gal80* combination, labeled histone signals in two adjacent cells should come from the ISC-restrictive transgene expression, having gone through an S phase to incorporate new H3 followed by partitioning new H3 into the two daughter cells. Using this strategy, similar patterns of old and new H3 inheritance patterns could be detected (Fig 3M and N), compared with the results using the *esg-Gal4* driver alone. Importantly, transgenes driven by either *esg-Gal4* itself or *esg-Gal4 Su(H)-Gal80* combination showed statistically insignificant difference in postmitotic pair cells with both asymmetric and symmetric Delta expression patterns (Fig 3O and P), indicating consistent results independent of the driver choice.

Similar results were obtained using the same histone labeling strategy in a parallel experimental regime, using an antibody that recognizes the Delta protein, for both H3 (Fig EV3A–C) and H2A (Fig EV3D–F) in postmitotic pairs (Ohlstein & Spradling, 2007; Bardin *et al*, 2010; Obniski *et al*, 2018). In summary, the cellular specificity of the postmitotic daughter cells with asymmetric versus symmetric Delta expression patterns demonstrates that asymmetric histone inheritance is correlated with asymmetric Delta expression in the postmitotic pairs of cells. Additionally, the differences between H2A and H3 or H4 are consistent with the previous biochemical results (Jackson, 1988; Xu *et al*, 2010) and recent discoveries in the *Drosophila* male GSCs (Tran *et al*, 2012; Wooten *et al*, 2019). This molecular specificity of asymmetric H3/H4 inheritance pattern is likely because reincorporation of the old $(H3\text{-}H4)_2$ tetramers greatly enhances the asymmetric distribution of old versus new H3/H4 between sister chromatids during DNA replication.

Expression of a mutant histone H3T3A disrupts asymmetric histone inheritance patterns and leads to increased ISC-like cells in the midgut

We have previously identified a mitosis-specific phosphorylation at Threonine 3 of H3 (H3T3ph) that can distinguish sister chromatids enriched with old versus new H3 in male GSCs, consistent with previous biochemistry results (Lin *et al*, 2016). Differential H3T3ph at old H3- versus new H3-enriched sister chromatids coordinate their proper recognition and segregation during ACD of male GSCs. By mutating the Thr3 residue on the H3 tail to an unphosphorylatable Alanine (H3T3A), the asymmetric segregation of old and new histones became randomized during ACD of male GSCs. Expression of this mutant histone in early-stage germ cells resulted in cellular defects, including progenitor germline tumors and significant germ cell loss, indicating that misregulation of asymmetric histone inheritance might affect proper cell identity establishment through ACD of GSCs (Xie *et al*, 2015, 2017). To further study the potential defects caused by H3T3A in the ISC lineage, we first expressed the dual-color version of this mutant histone using the *esg-Gal4* driver. We then studied the distribution of old versus new H3T3A throughout different mitotic stages in ISCs. When examining prophase and prometaphase ISCs, distinct old versus new H3T3A domains could still be detected, similar to the pattern found for old versus new H3 at the comparable stages in mitotic ISCs (Figs 4A versus 1D). When conducting Pearson's correlation analyses, the average correlation coefficient of 0.49 for old versus new H3T3A was close to the average correlation coefficient of 0.44 for old versus new H3, both of which were significantly lower than the average correlation coefficient of 0.71 for old versus new H2A (Fig 4B) and the two signals expressed from the *UAS-H3-eGFP-T2A-H3-mCherry* transgene (correlation coefficient = 0.86, Fig 1F). The Spearman correlation coefficient yielded similar results from assessing the colocalization of old versus new H3T3A (Fig EV1F). These results indicate that old and new H3T3A is still differentially incorporated onto sister chromatids prior to mitosis, similar to the wild-type H3.

However, when compared to wild-type old H3, old H3T3A distributed more equally between the two segregated sets of sister chromatids compared with H3 in anaphase and telophase ISCs (Figs 4C versus 2D). When quantified, the segregation pattern of H3T3A was more similar to that of H2A than to H3 (Fig 4D). Consistently, even though both Delta-asymmetric and Delta-symmetric postmitotic pairs of cells could be detected (Fig 4E and F), a decrease in the Delta-asymmetric pairs with an increase in the Delta-symmetric pairs was detected in the H3T3A-expressing ISC lineage compared with the H3-expressing ISC lineage (Fig 4G). Collectively, these data suggest that old versus new H3T3A are differentially incorporated into sister chromatids prior to mitosis, similar to H3; however, this mutation disrupts the differential recognition of sister chromatids, leading to more symmetric segregation of H3T3A in mitotic ISCs. Notably, these results are in accordance with the previous findings regarding the incorporation and segregation modes of old versus new H3T3A in the male GSCs (Xie *et al*, 2015), suggesting a potentially conserved mechanism in both stem cell systems.

In the H3-expressing midguts, Dl-nLacZ-positive cells are normally detected as single-cell colonies in an evenly dispersed manner. Two Dl-nLacZ-positive cells were occasionally adjacent to each other, potentially as a result of a symmetric ISC division (Fig 4H). In contrast, a disorganized pattern of Dl-nLacZ-positive cells could be detected in the H3T3A-expressing midgut, with an appearance of increased Delta-positive cell clusters with more than one cell (Fig 4I). Using a clustering analysis, we quantified the relative frequencies of cell cluster(s) with 1-, 2-, or ≥ 3 Dl-nLacZ-positive cells (Fig 4J). These analyses revealed a substantial increase of the 2- and ≥ 3-Dl-nLacZ cell clusters in the H3T3A-expressing midguts as compared to the H3-expressing midguts, potentially suggesting an increased ratio of SCD and overpopulated ISC-like cells in the H3T3A-expressing midguts (Fig 4K). This increase is at the expense of single Dl-nLacZ cells, which displayed a significant decrease from 77.9% in the H3-expressing midguts to 50.4% in the H3T3A-expressing midguts (Fig 4L). Additionally, a significantly higher number of mitotic cells were detected as H3S10ph-positive cells in the H3T3A-expressing samples compared with the H3-expressing samples (Fig 4M). These results support the detection of increased Delta-symmetric pairs, likely due to increased symmetric outcome of ISC divisions, in the H3T3A-expressing midguts (Fig 4G).

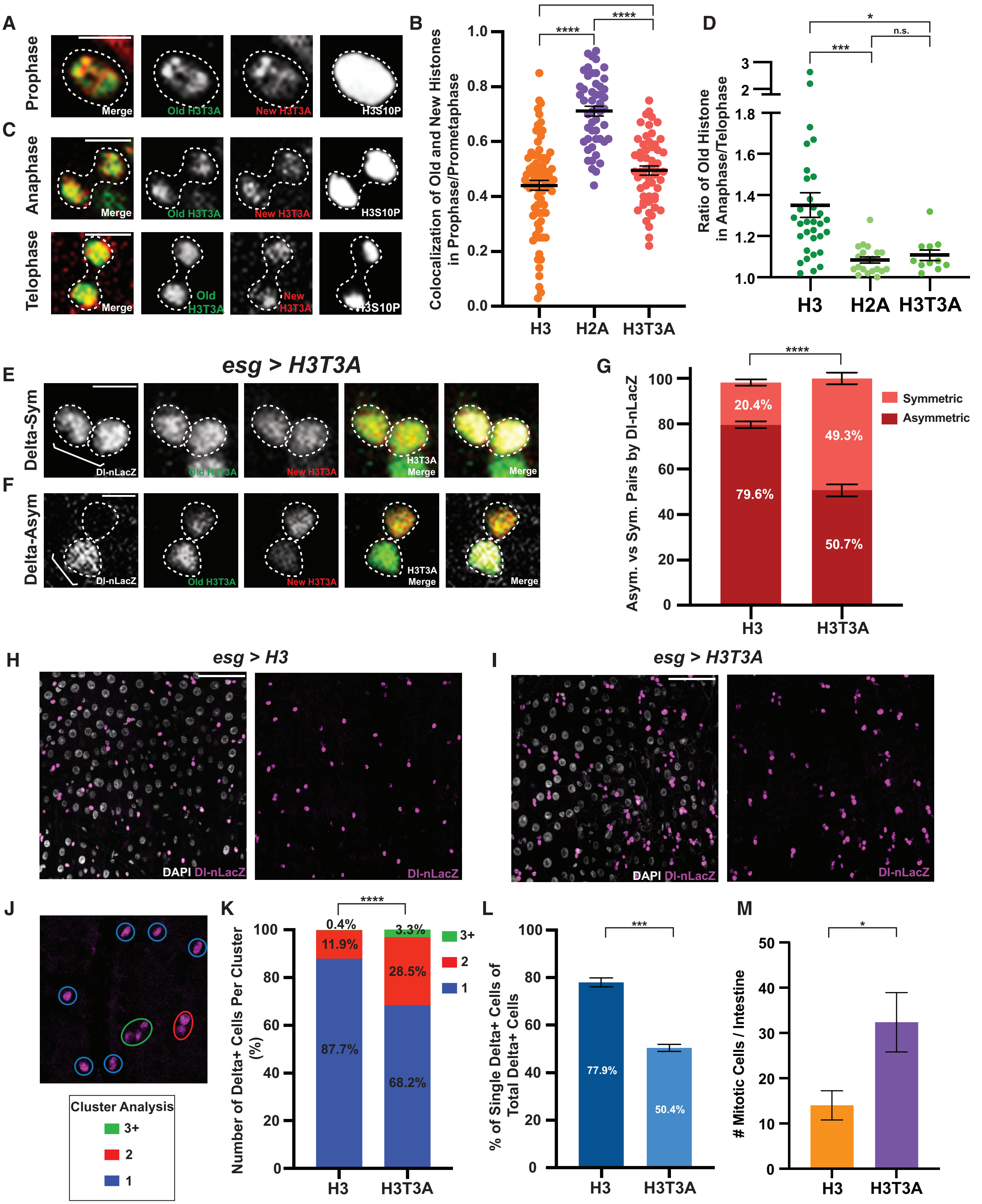

Figure 4.

 © 2023 The Authors

Figure 4. Expression of a mutant histone H3T3A disrupts histone inheritance patterns and increases Delta-symmetric pairs of cells.

A Old and new H3T3A distribution in prophase ISC, showing separable domains between H3T3A-GFP (old) and H3T3A-mKO (new) (*esg-Gal4 > UASp-FRT-H3T3A-eGFP-FRT-H3T3A-mCherry*).

B Quantification of colocalization between H3T3A-GFP (old) and H3T3A-mKO (new) in prophase and prometaphase ISCs using Pearson's correlation coefficients. The H3 and H2A data are from Fig 2C for direct comparison. Old and new H3T3A distribution is more similar to that of H3 (Pearson's correlation coefficient for H3T3A = 0.49 ± 0.02, n = 55 ISCs). Individual data points and mean values are shown. Error bars represent SEM. ****P < 0.0001, *P < 0.05; unpaired t-test to compare two individual datasets to each other. Individual data values are shown in Dataset EV1.

C Anaphase and telophase ISCs showing mostly symmetric segregation of H3T3A-GFP (old) and H3T3A-mKO (new) between the two sets of sister chromatids at the opposite poles of the ISC.

D Quantification of old histone distribution between the two sets of sister chromatids for anaphase and telophase ISCs expressing H3T3A (avg. old H3T3A ratio = 1.11 ± 0.03, n = 11 ISCs). The H3 and H2A data are from Fig 2G for direct comparison. The H3T3A segregation pattern is more similar to H2A than to H3. Individual data points and mean values are shown. Error bars represent SEM. *P < 0.05, n.s.: not significant; unpaired t-test to compare two individual datasets to each other. Individual data values are shown in Table EV1.

E In a postmitotic pair of cells with comparable Dl-nLacZ, both H3T3A-GFP (old) and H3T3A-mKO (new) are symmetrically distributed between the two cells.

F In a postmitotic pair of cells with asymmetric Dl-nLacZ expression, H3T3A-GFP (old) is enriched in the Delta-high cell, while H3T3A-mKO (new) is more toward the Delta-low cell.

G Quantification of Delta-asymmetric and Delta-symmetric pairs in wild-type H3-expressing midguts (avg. asymmetric = 79.6 ± 1.5%, avg. symmetric = 20.4 ± 1.4% N = 6 midguts, n = 2,341 pairs) and H3T3A-expressing midguts (avg. asymmetric = 50.7 ± 2.6%, avg. symmetric = 49.3 ± 2.6%, N = 6 midguts, n = 2,745 pairs). Bars represent mean proportions of asymmetric and symmetric pairs. Error bars represent SEM. Comparing H3T3A-expressing midguts with H3-expressing midguts, there was a statistically significant difference in the distributions of Delta-symmetric pairs versus Delta-asymmetric pairs, determined by a chi-square test (X^2(1, n = 2,745) = 1,309.56, ****P < 0.0001). The H3T3A-expressing midguts show an increase in Delta-symmetric pairs and a decrease in Delta-asymmetric pairs when compared to H3-expressing midguts. Individual data values are shown in Table EV2.

H Distribution of the Dl-nLacZ-positive (magenta) cells in an H3-expressing midgut, which are well spaced and interspersed within the intestinal epithelium.

I Distribution of the Dl-nLacZ-positive (magenta) cells in an H3T3A-expressing midgut, which are unevenly distributed in clusters with two or more Delta-positive cells. DAPI: white in (H, I).

J Cluster analyses show representative 1-, 2-, or ≥ 3-Delta-positive cell clusters.

K Percentages of clusters with 1-, 2-, or ≥ 3-Delta-positive cell clusters for H3-expressing midguts (avg. percentage of 1-Dl^+ cells = 87.7 ± 1.1%, avg. percentage of 2-Dl^+ cells = 11.9 ± 0.9%, avg. percentage of ≥ 3-Dl^+ cells = 0.4 ± 0.2%, N = 4 midguts, n = 1,482 clusters) and H3T3A-expressing midguts (avg. percentage of 1-Dl^+ cells = 68.2 ± 1.2%, avg. percentage of 2-Dl^+ cells = 28.5 ± 0.9%, avg. percentage of ≥ 3-Dl^+ cells = 3.3 ± 0.4%, N = 5 midguts, n = 1,862 clusters). Bars represent mean proportions of cell clusters with 1, 2, or 3^+ Dl^+ cells out of all cell clusters counted. Error bars represent SEM. Comparing H3-expressing midguts with H3T3A-expressing midguts, there was a statistically significant difference in the distributions of Delta-positive cells per cluster, determined by a chi-square test (X^2(2, n = 1,862) = 908.75, ****P < 0.0001). As shown, there are significant increases in 2 and ≥ 3-Delta-positive cells per cluster in the H3T3A-expressing intestines when compared to the H3-expressing intestines. Individual data values are shown in Table EV3.

L Percentages of single Delta-positive cells out of total Delta-positive cells for H3-expressing midguts (avg. single Dl^+ cells = 77.9 + 1.9%, N = 4 midguts, n = 1,674 Dl^+ cells) and H3T3A-expressing midguts (avg. single Dl^+ cells = 50.4 ± 1.5%, N = 5 midguts, n = 2,522 Dl^+ cells). Bars represent mean proportion of single Dl^+ ISCs out of all ISCs counted. Error bars represent SEM. There are fewer single Delta-positive cells in H3T3A-expressing midguts compared with H3-expressing midguts (two-sample t-test, ***P < 0.001).

M Comparison of the mitotic index in H3-expressing (N = 8 midguts) and H3T3A-expressing (N = 8 midguts) midguts. Total number of mitotic ISCs was quantified per midgut by the presence of H3S10 phosphorylation mark. H3T3A-expressing midguts had a significantly higher mitotic index when compared to H3-expressing midguts (two-sample t-test, *P < 0.05). Bars represent mean number of mitotic cells per midgut. Error bars represent SEM. Individual data values are shown in Table EV4.

Data information: individual n and N values are defined per panel. Scale bar in (A), (C), (E), (F): 5 µm, in (H) and (I): 50 µm; asterisk, ISC side.

Because the mutant histone H3T3A expression resulted in noticeable changes in the midgut, we aimed to eliminate any potential secondary defects caused by its expression during midgut development, using the combination of the Gal4 activator driven by the *esg* promoter (*esg-Gal4*) with both the Gal80 suppressor driven by the *Su(H)* promoter [*Su(H)-Gal80*] and the temperature-sensitive Gal80 driven by the *tubulin* promoter (*tub-Gal80^{ts}*). These combined drivers allow both spatial [by *Su(H)-Gal80*] and temporal (by *tub-Gal80^{ts}*) control to turn on an mCherry-tagged mutant H3T3A exclusively in ISCs and only after a temperature shift in adult flies. An mCherry-tagged wild-type H3 was used as the control. In addition, a *UAS-eYFP* was used as the reporter to indicate the activity of the spatiotemporally controlled Gal4 driver and to locate ISCs.

We first made sure that under the permissive condition of *tub-Gal80^{ts}* at 18°C, no Gal4 activity could be detected, shown as no detectable reporter (i.e., eYFP) or histone transgene (i.e., mCherry) signal for both H3 and H3T3A lines (Fig 5A and C). Upon shifting to the restrictive temperature at 29°C to inactivate the ubiquitous *tub-Gal80* suppressor, both eYFP reporter and histone-mCherry expression could be detected in H3 as well as H3T3A lines (Fig 5B and D). When studying the eYFP pattern along with either an ISC marker Dl-nLacZ or an EB marker Su(H)-LacZ, eYFP displayed tight colocalization with Dl-nLacZ but adjacent signals with Su(H)-LacZ (Fig EV3G and H), confirming that it can be used as an ISC reporter. Examining the morphology of the spatiotemporally controlled H3T3A-expressing midguts revealed unevenly distributed ISCs shown by the eYFP reporter (Fig 5F), compared with the H3-expressing midguts with evenly spaced eYFP-labeled ISCs (Fig 5E). The ISC distribution pattern in the control H3-expressing midgut is consistent with previous reports (Ohlstein & Spradling, 2007; Ahmed *et al*, 2020). To further analyze these phenotypes more precisely and quantitatively, we utilized a line measurement that quantified the averaged pixel intensity of the eYFP signal at each pixel length along the profile of the intestine. Plotting these measurements showed distinct profiles of the H3- versus H3T3A-expressing midguts (Fig 5G). To compare multiple H3- versus H3T3A-expressing midgut profiles together, we took the pixel intensity measurements given in the line measurement and averaged every 1,000-pixel length to obtain one unique data point. When plotting these averaged eYFP pixel intensity measurements from H3- versus

© 2023 The Authors

H3T3A-expressing midguts, significantly increased signals in the -H3T3A-expressing midguts were found compared with the H3-expressing ones (Fig 5H). These greatly enhanced signal intensities of the ISC-labeling reporter suggest overpopulated ISC-like cells, consistent with results shown above (Fig 4K). Additionally, measuring the mitotic index in the spatiotemporally controlled H3T3A-expressing midguts demonstrated an almost twofold increase of the H3S10ph-positive cells compared with those H3-expressing midguts (Fig 5I), consistent with the results shown above (Fig 4M).

Next, using a qualitative assay by classifying the midgut morphology phenotypes into strong, mild and no phenotype categories (see legend for Fig 5J), no H3-expressing midguts displayed strong phenotype, 27.3% showed mild and 72.7% showed no phenotype. Contrastingly, 54.5% H3T3A-expressing midguts displayed a strong phenotype, 27.3% showed mild, and only 18.2% showed no phenotype (Fig 5J and K). In summary, these results demonstrate that a controlled expression of the H3T3A mutant histone leads to misregulation of histone inheritance and defects in the ISC lineage. Notably, these results are consistent with the previous findings by expressing the mutant H3T3A in the early-stage male germline, where progenitor germline tumor phenotype could be detected (Xie *et al*, 2015), suggesting potentially conserved requirements of asymmetric histone inheritance in these two adult stem cell systems to guide both the stem daughter cell for self-renewal and the differentiating daughter cell for proper differentiation.

The increased ISC-like cells could arise from misdetermination of cell fates

To further understand the consequences of expressing the H3T3A mutant in the ISC lineage, we utilized the same spatiotemporally controlled experimental design to express either H3-mCherry or H3T3A-mCherry, together with different cell identity markers. For example, a Su(H)-LacZ reporter was used as the EB cell marker (Fig 6A and B). In H3-expressing intestines, the ISC-specific eYFP signals and the EB-specific LacZ signals often display separable signals in the two adjacent cells (Fig 6A). In contrast, in H3T3A-expressing intestines, regions with disorganized and less separable eYFP and LacZ signals were often detected (Fig 6B). Using Pearson's colocalization analysis to compare the colocalization of the eYFP and LacZ signals, the H3T3A-expressing intestinal regions showed a higher degree of colocalization than the H3-expressing

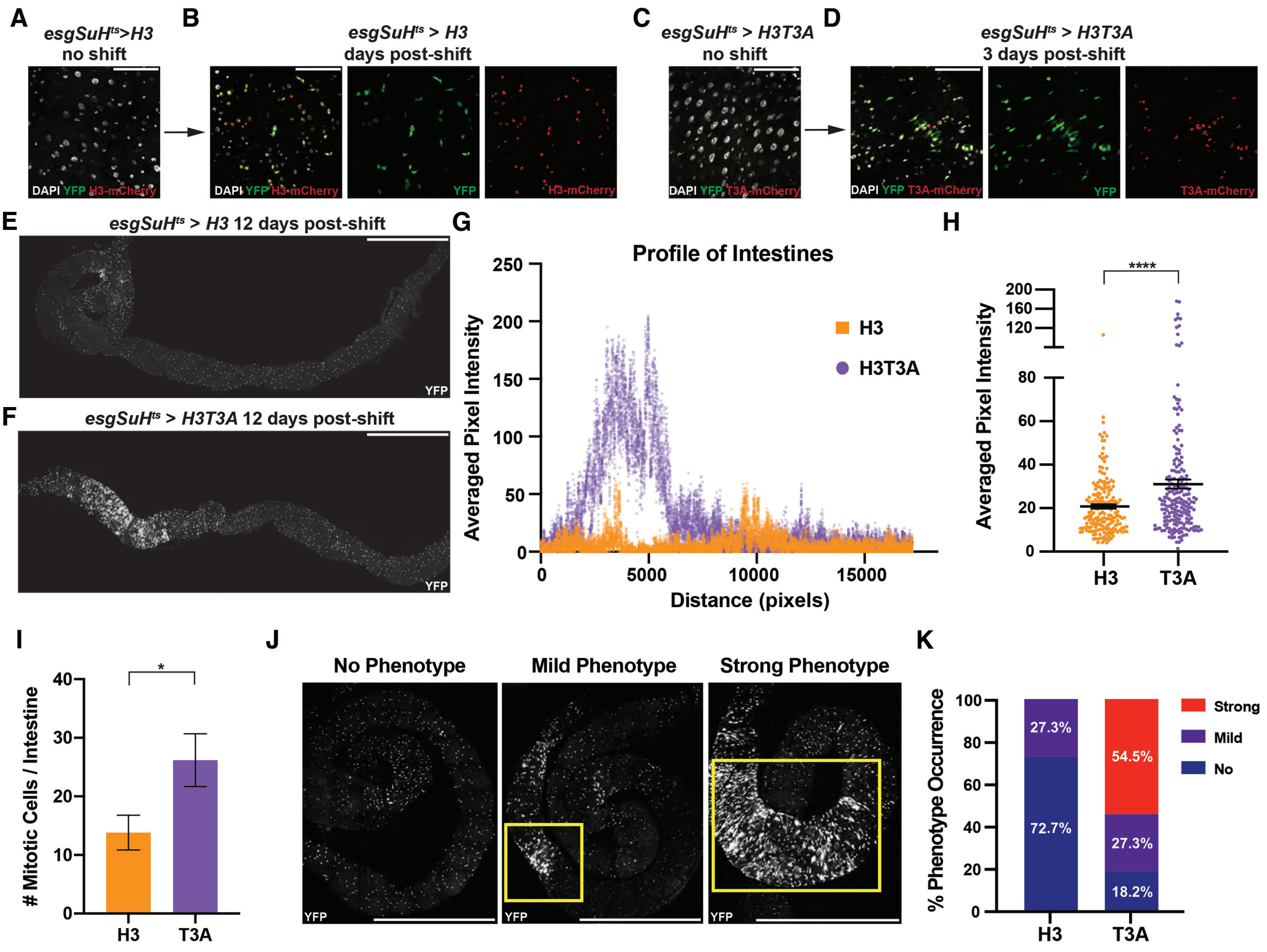

Figure 5.

 © 2023 The Authors

Figure 5. Spatiotemporally controlled expression of the mutant histone H3T3A leads to overpopulated Delta-positive ISC-like cells.

A A view of the midgut of an *UAS-H3-mCherry* transgene driven by *esg-Gal4*, *Su(H)-Gal80*, *tub-Gal80^ts^* at the Gal80^{ts} permissive temperature (18°C), showing no expression of the histone transgene or the UAS-eYFP reporter that marks ISCs.

B A view of the midgut of an *UAS-H3-mCherry* transgene driven by *esg-Gal4*, *SuH-Gal80*, *tub-Gal80*^{ts} at the Gal80^{ts} restrictive temperature (29°C), showing expression of the H3-mCherry transgene (red) and the UAS-eYFP reporter (green).

C A view of the midgut of an *UAS-H3T3A-mCherry* transgene driven by *esg-Gal4*, *SuH-Gal80*, *tub-Gal80*^{ts} at the Gal80^{ts} permissive temperature (18°C), showing no expression of the histone transgene or the UAS-eYFP reporter.

D A view of the midgut of an *UAS-H3T3A-mCherry* transgene driven by *esg-Gal4*, *SuH-Gal80*, *tub*-Gal80^{ts} at the Gal80^{ts} restrictive temperature (29°C), showing expression of the H3T3A-mCherry transgene (red) and the UAS-eYFP reporter (green).

E H3-expressing midgut for 12 days at 29°C. Midgut looks largely normal, with an even distribution of mostly single eYFP-positive ISCs throughout the midgut.

F H3T3A-expressing midgut for 12 days at 29°C. ISCs are unevenly distributed throughout the midgut, and a large region with overpopulated eYFP-positive ISC-like cells can be visualized.

G Quantitative profiles of eYFP reporter signal of intestines in (E) and (F). The average pixel intensity of the eYFP reporter signal throughout the length of the intestine was taken at each pixel length along each intestine. These values are plotted, showing the profile of these samples.

H Quantification of average pixel intensity of the eYFP reporter signal in H3-expressing midguts (N = 11 midguts) and H3T3A-expressing midguts (N = 12 midguts). Each intestine was subjected to profile measurement, giving average pixel intensities of the eYFP signal along the length of the intestine. Every 1,000 measurements were averaged to give one data point and plotted on this graph (For H3, n = 188 averaged measurements; for H3T3A, n = 204 averaged measurements). Individual data points and mean values are shown. Error bars represent SEM. There is a significant increase in the average eYFP signal in regions of H3T3A-expressing midguts when compared to H3-expressing midguts (two-sample t-test, ****P < 0.0001). Individual data values are shown in Dataset EV3.

I Comparison of the mitotic index in H3-expressing (N = 11 midguts) and H3T3A-expressing (N = 11 midguts) midguts. Total number of mitotic ISCs was quantified per midgut by the presence of H3S10 phosphorylation mark. Bars represent mean number of mitotic cells per midgut. Error bars represent SEM. H3T3A-expressing midguts had a significantly higher mitotic index when compared to H3-expressing midguts (two-sample t-test, *P < 0.05). Individual data values are shown in Table EV5.

J Qualitative analysis of midgut phenotypes. No phenotype is called when the ISCs appear normally distributed based on eYFP reporter signal. A mild phenotype is called when there is a small region of eYFP-positive cells clustered within a region, showing mild disorganization. A strong phenotype is called when there is a large region of eYFP-positive cells clustered, showing significant disorganization.

K Percentage of H3-expressing midguts (N = 11 midguts) and H3T3A-expressing midguts (N = 11 midguts) with no phenotype, a mild phenotype, or a strong phenotype.

Data information: Scale bars in (A), (B), (C), and (D): 50 μm, in (E), (F) and (J): 500 μm.

regions, suggesting a possibility of misdetermined cell fate in the H3T3A-expressing intestines (Fig 6C). In addition to ISCs and EBs, cellular markers of other cell types, such as ee cells and ECs, were investigated using the ee-specific Prospero expression and the EC-specific polyploidy feature with DAPI staining. No significant difference of ee or EC cells could be detected in the H3T3A-

Figure 6. Increase of ISC-like cells is likely due to misdetermination of cell fates.

A A view of the midgut of an *UAS-H3-mCherry* transgene driven by *esg-Gal4*, *SuH-Gal80*, *tub-Gal80*^{ts} at the Gal80^{ts} restrictive temperature (29°C), showing expression of the H3-mCherry transgene (gray) and the UAS-eYFP reporter (green) from the driver as well as expression of the Su(H)-LacZ reporter line (magenta). eYFP and Su(H)-LacZ signals are organized and separable.

B A view of the midgut of an *UAS-H3T3A-mCherry* transgene driven by *esg-Gal4*, *SuH-Gal80*, *tub-Gal80*^{ts} at the Gal80^{ts} restrictive temperature (29°C), showing expression of the H3T3A-mCherry transgene (gray) and the UAS-eYFP reporter (green) from the driver as well as expression of the Su(H)-LacZ reporter line (magenta). eYFP and Su(H)-LacZ signals are disorganized and overlapping.

C Quantification of colocalization between eYFP and Su(H)-LacZ using Pearson's correlation coefficients. Consistent square regions of 150 μm by 150 μm were analyzed in H3- and H3T3A-expressing intestines. H3T3A-expressing intestine regions showed a higher degree of colocalization between eYFP and Su(H)-LacZ signals when compared to H3-expressing intestines (Pearson's correlation coefficient for H3T3A = 0.24 ± 0.10, n = 42 regions, Pearson's correlation coefficient for H3 = 0.16 ± 0.07, n = 42 regions). Individual data points and mean values are shown. Error bars represent SEM. ****P < 0.0001; unpaired t-test to compare two individual datasets to each other. Individual data values are shown in Table EV6.

D Quantification of the number of two differentiated cell types, ee cells and ECs in a region of 150 μm by 150 μm. There is no significant different in number of ee cells and ECs between H3- and H3T3A-expressing intestines (for ee cells: H3 = 20.57 ± 2.02, n = 42 regions; H3T3A = 19.31 ± 1.81, n = 42 regions; For ECs: H3 = 78.11 ± 3.92, n = 28 regions; H3T3A = 84.14 ± 4.27, n = 28 regions). Individual data points and mean values are shown. Error bars represent SEM. NS, not significant; unpaired t-test to compare two individual datasets to each other. Individual data values are shown in Table EV7.

E Clusters, in a *UAS-H3-mCherry* transgene sample, may be labeled as one of several midgut cell types on the basis of several marker genes. Marker gene levels are presented in Fig EV5.

F Separate analysis of the *UAS-H3T3A-mCherry* transgene sample identified cell types. These labeled populations were similar to the H3 cell populations. Given the difference in barcode metrics, these plots are not amenable to a visual comparison of differences in population dynamics.

G Detail of esg^{+} stem-like cells in *UAS-H3-mCherry*, quantifying stem cell marker Delta (Dl), and progenitor marker Klumpfuss (klu). We demonstrate the near-absence of Dl^{+}klu^{+} cells, and the lack of separability of populations using this PCA-UMAP method.

H Detail of esg^{+} stem-like cells in *UAS-H3T3A-mCherry*, quantifying Delta and Klumpfuss. This sample is visually similar under UMAP to the *UAS-H3-mCherry* sample.

I The pseudotime model Slingshot (placing cells on the x-axis) separates Dl^{+} and Dl^{-} cells in the esg^{+} cluster (levels indicated on the log y-axis). The regression model glmGamPoi demonstrates differential expression of Dl in pseudotime, with a slightly enhanced fit (nonzero slope) in the wild-type condition (n.s. P = 0.08).

J In pseudotime, klu is differentially expressed in the H3 wild-type sample, with a slope opposite to that of Dl (demonstrating inverse correlation). The Slingshot pseudotime model is deficient in separating klu^{+} and klu^{-} cells in the H3T3A mutant sample, producing a regression fit without the expected positive slope. Our regression model produces different slopes for the H3 line and the H3T3A line, with a strongly nonzero coefficient for the difference in slope (****P < 0.0001). This supports a hypothesis that subpopulations of esg^{+} cells (klu^{+}, klu^{-}) may be less separable by their transcriptome profiles in *UAS-H3T3A-mCherry*, as compared to *UAS-H3-mCherry*.

Data information: Scale bars in (A) and (B): 50 μm.

© 2023 The Authors

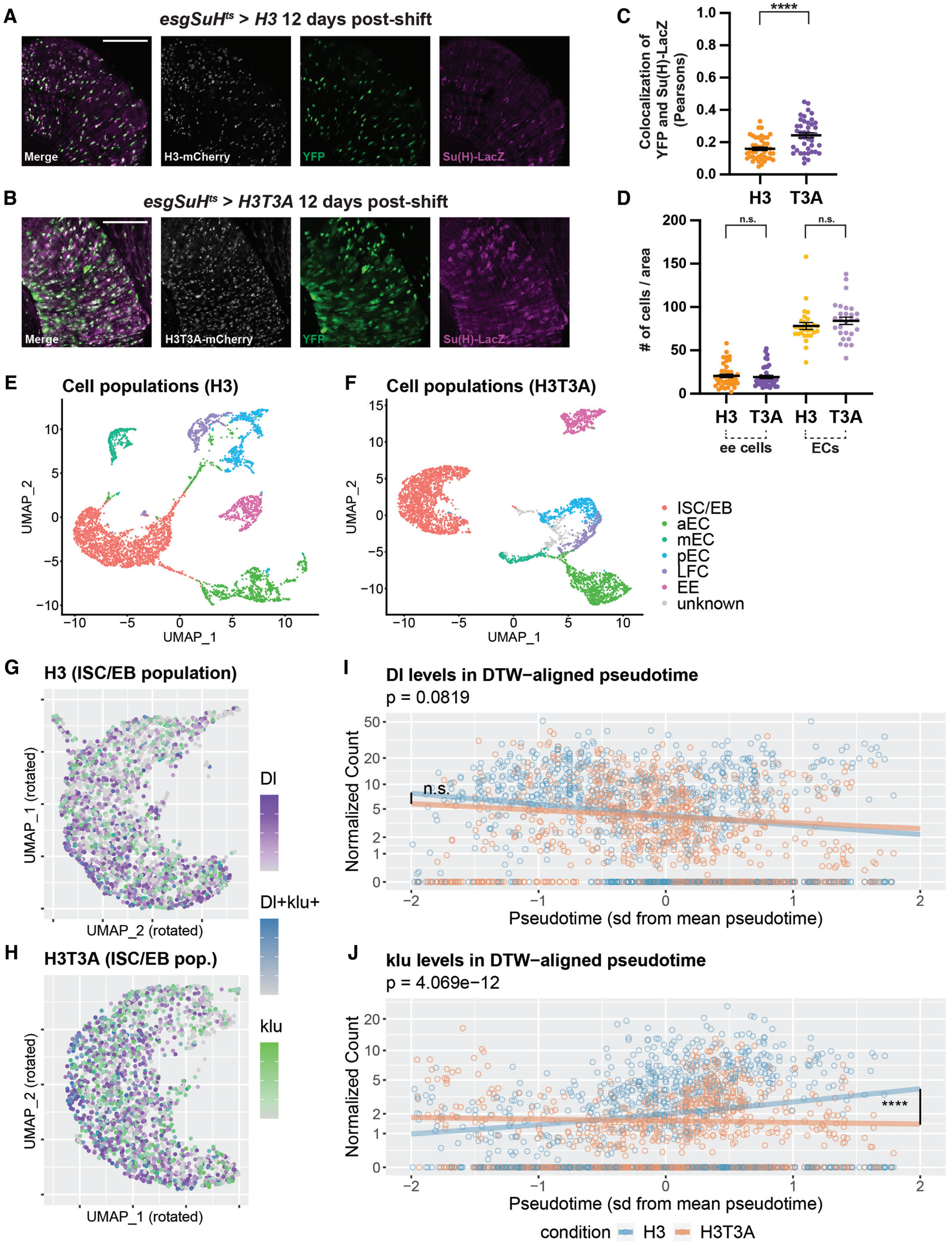

Figure 6.

© 2023 The Authors

expressing compared with the H3-expressing intestines (Fig 6D). Overall, these imaging-based data indicate that there is no detected difference of the ee and EC cells in the H3- versus H3T3A-expressing intestines.

To confirm these potential effects in the ISC lineage, we performed single-cell RNA-seq (sc RNA-seq, see Materials and Methods) of the H3- and H3T3A-expressing midgut samples. We identified cluster identities closely matching previously identified midgut cell clusters (Hung *et al*, 2020), with the following prevalence: EC cells (aEC + mEC + pEC) have a prevalence of 40% in H3- and 38% in H3T3A-expressing samples, while ee cells are 9% in H3- and 12% in H3T3A-expressing samples (Fig 6E and F). These prevalence values are consistent with the above staining results. However, Seurat's preprocessing, UMAP, and clustering (Satija *et al*, 2015) did not separate ISC and EB cells into two populations (Fig 6G and H), so we report on a combined (ISC + EB) population. ISC + EB prevalence is 43% in H3- and 45% in H3T3A-expressing samples (Fig 6E and F). Next, using temporally differential gene expression analyses (Figs EV4A–H and EV5), *klumpfuss* (*klu*) was identified as a differentiation gene that is absent in *delta*-expressed cells. This finding is consistent with the previous report of the function of klu as a transcription factor (Korzelius *et al*, 2019), and with the prior hypothesis of *klu* as an EB marker in scRNA-seq (Hung *et al*, 2020), but we produced enhanced localization in pseudotime after the application of SCTransform normalization with Slingshot analysis (Hafemeister & Satija, 2019). Linear trends in pseudotime are characterized using Gamma-Poisson regression (Ahlmann-Eltze & Huber, 2021). For the two scRNA-seq samples here, our regression model produced the coefficient named *pseudotime:conditionH3T3A*, representing difference in slope of the two pseudotime trend lines. Delta levels in pseudotime did not differ significantly between the two samples ($P = 0.08$, Fig 6I), while *klu* was differentially expressed in H3 pseudotime, with a slope significantly different from that of the H3T3A pseudotime ($P < 10^{-4}$, Fig 6J). Together, here the scRNA-seq data confirm that spatiotemporally controlled expression of the mutant histone H3T3A does not affect major cell types in the ISC lineage, but compromises differentiation of ISCs into a recognizable, klu^{+}, EB cell profile.

Discussion

A long-standing biological question is how distinct cell fates are established, maintained, and changed by epigenetic mechanisms at the single-cell level in multicellular organisms, where cells have identical genomes. Here, using the *Drosophila* ISC lineage as a model system, we studied the inheritance of different canonical histones H3, H4, and H2A during the ISC divisions. In postmitotic pairs of cells with an asymmetric Delta expression pattern, asymmetric histone inheritance patterns are detected, where the Delta-high cell inherits more old histones and the Delta-low cell inherits more new histones. In contrast, histones are distributed more symmetrically between the postmitotic pair of cells with similar Delta expression. We hypothesize that the observed differences in Delta expression patterns are indicative of distinct cell identities resulting from different cell division modes. Here, an asymmetric Delta expression pattern, with one Delta-high cell and one Delta-low cell, may indicate that these two cells result from an asymmetric ISC division, where the Delta-high cell will maintain an ISC identity while the Delta-low cell will take on differentiation. In contrast, a symmetric Delta expression pattern could result from a symmetric ISC division, where both Delta-expressing cells will maintain the ISC identity. Interestingly, the percentages of asymmetric versus symmetric Delta expression patterns in the postmitotic pairs (79.6% versus 20.4% in Fig 4G) are largely consistent with the ratios of asymmetric versus symmetric ISC divisions as reported in other studies (O'Brien *et al*, 2011; de Navascues *et al*, 2012; Goulas *et al*, 2012; Tian & Jiang, 2014; Jin *et al*, 2017), as well as the H3 inheritance modes in the mitotic ISCs observed in our study (75.0% versus 25.0% in Fig 2H). These findings link the asymmetric histone inheritance mode with the establishment of distinct cell identities after one cell division. Furthermore, we found that this asymmetric inheritance mode is specific to H3 and to the less extent to H4 (Fig 7A), as old and new H2A histones are almost always inherited symmetrically (Fig 7B). This molecular specificity could be explained by the incorporation of old H3 and H4 into chromatin as a $(H3\text{-}H4)_2$ tetramer, while old H2A and H2B are incorporated as (H2A-H2B) dimers (Jackson & Chalkley, 1981; Russev & Hancock, 1981; Jackson, 1988; Xu *et al*, 2010; Katan-Khaykovich & Struhl, 2011). Since H3 and H4 carry most of the post-translational modifications that influence gene expression (Kouzarides, 2007; Young *et al*, 2010; Huang *et al*, 2014; Allis & Jenuwein, 2016), the asymmetric inheritance of old versus new $(H3\text{-}H4)_2$ serves as an elegant mechanism for establishing distinct epigenomes in the daughter cells that arise from stem cell ACD, possibly leading to differential gene expression programs and potentially other distinct cellular features.

The biological significance of the asymmetric histone inheritance is further exemplified when this pattern is disrupted by the H3T3A histone mutant, where we detected an increase in Delta-symmetric postmitotic pairs of cells as well as the overpopulated ISC-like cells (Fig 7C). Interestingly, based on intestinal morphology, we observed hyperplasia in the H3T3A mutant more often in the posterior midgut. Previous work in the field has identified the posterior midgut as

Figure 7. Model for old (green) and new (red) histone inheritance patterns and their potential roles in the ISC system.

A Old versus new H3 and H4 histones are likely incorporated differentially during S phase and form separable domains visualized in the mitotic ISCs at prophase and prometaphase. During ACD, old versus new H3- and H4-enriched sister chromatids are segregated asymmetrically, giving rise to a self-renewed ISC that inherits more old H3 and H4, and a non-ISC that inherits more new H3 and H4. During SCD, old versus new H3- and H4-enriched sister chromatids are segregated symmetrically, giving rise to two ISCs that inherit a comparable amount of old versus new H3 and H4.

B Old versus new H2A histones are likely incorporated evenly during S phase and show an overlapping distribution visualized in the mitotic ISCs at prophase and prometaphase. In both ACD and SCD, old versus new H2A are segregated symmetrically to the resulting daughter cells.

C With the mutant histone H3T3A, there is a significant increase in Delta-symmetric postmitotic pairs when compared to wild-type H3 shown in (A), suggesting a potential increase in SCD and defective cellular differentiation in the ISC system.

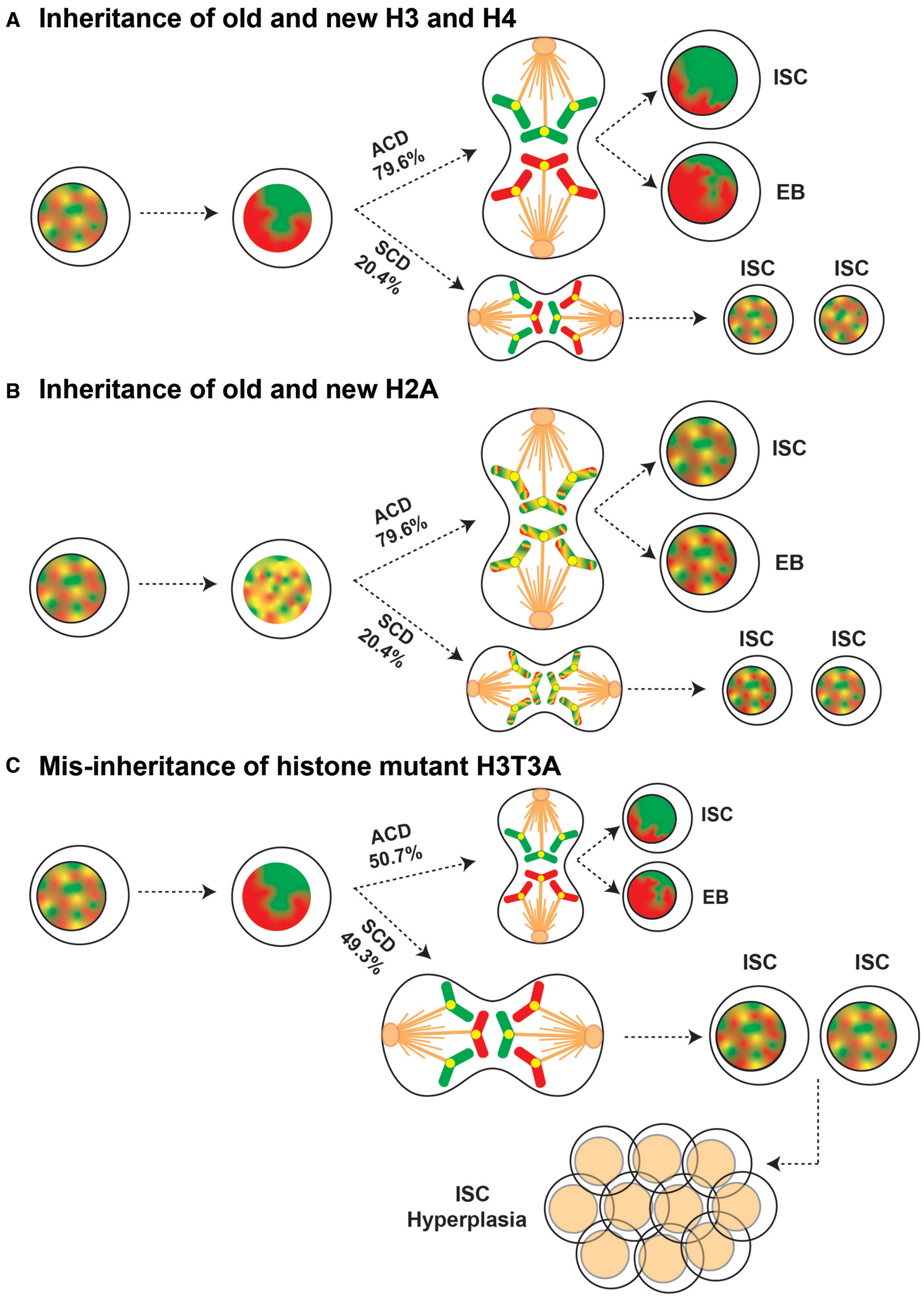

Figure 7.

© 2023 The Authors

a region that is more sensitive to tumor formation, acting as a "tumor hotspot" (Marianes & Spradling, 2013; Patel *et al*, 2015). In our experiments, we see that the posterior midgut appears to be more sensitized to the H3T3A mutant, consistent with these findings. These data connect misregulated histone inheritance with potential changes in cell fate determination. Recently, large-scale analysis identified histone mutations in 3.8% of human tumor samples, a ratio similar to the mutations of known cancer-associated genes such as *BRCA2* and *NOTCH1*. In particular, mutations at the Thr3 residue of H3 have been found in a variety of human tumor samples, including lung, breast, skin, bladder, and liver cancers (Nacev *et al*, 2019). However, the molecular mechanisms underlying these "oncohistones" are not fully understood. Here, our findings on the oncohistone H3T3A illuminate how this mutation could lead to the loss of proper epigenetic inheritance and the onset of tumor formation.

The asymmetric inheritance mode of histones was first reported during the ACD of male *Drosophila* GSCs (Tran *et al*, 2012), which opened a new avenue of research; however, many essential questions remained, such as whether this asymmetric histone inheritance mode is specific to germ cells, stem cells, and/or asymmetrically dividing cells. The results reported in this paper provide a solid basis to addressing these questions. Despite significant differences in the niche structure, signaling cascades for regulating stem cell activity, and cellular differentiation pathways between the ISC and male GSC lineages [(Kiger *et al*, 2001; Tulina & Matunis, 2001; Ohlstein & Spradling, 2007; Leatherman & Dinardo, 2008; Lin *et al*, 2008; Jiang *et al*, 2009, 2011; Amoyel *et al*, 2014; Li *et al*, 2014; Stine *et al*, 2014; Doupe *et al*, 2018) and reviewed in (Morrison & Spradling, 2008; de Cuevas & Matunis, 2011; Losick *et al*, 2011; Kahney *et al*, 2019)], several key features of asymmetric histone inheritance are common between these two stem cell systems. First, the cellular specificity in the GSC lineage has been demonstrated by asymmetric histone inheritance mode in asymmetrically dividing GSCs but not in symmetrically dividing spermatogonial cells. In the ISC lineage, this cellular specificity is manifested by ISCs displaying an asymmetric histone inheritance mode in Delta-asymmetric postmitotic pairs but not for Delta-symmetric postmitotic pairs. Second, this asymmetry has the molecular specificity for H3 and H4 histones in both systems, emphasizing the importance of these two canonical histones in carrying and passing on or resetting an "epigenetic memory." Finally, expression of the mutant histone H3T3A abolishes asymmetric histone inheritances in both systems, resulting in stem cell or progenitor cell hyperplasia. Therefore, these results demonstrate that the asymmetric histone inheritance mode is not specific to either germ cells or stem cells, but likely contingent on the asymmetric mode of mitosis with the mission to generate two distinct daughter cells. Additionally, asymmetric histone inheritance could occur at a local level, likely at critical genes that regulate stem cell fate or proper cellular differentiation (Kahney *et al*, 2021). Similar local asymmetry of histone inheritance was shown in induced asymmetrically dividing mouse embryonic stem cells (mESCs; *Ma et al*, 2020), with the H3K27me3 as a key histone modification displaying distinct distribution at the differentially expressed genes between the two daughter cells (Sun *et al*, 2021). It will be intriguing to explore in the future what histone modifications are associated with old versus new histones.

Furthermore, asymmetric sister centromere in recognizing sister chromatids during ACD has also been reported in *Drosophila* male GSCs (Ranjan *et al*, 2019; Kochendoerfer *et al*, 2021, 2023; Ranjan & Chen, 2022) and female GSCs (Carty & Dunleavy, 2020; Dattoli *et al*, 2020; Carty *et al*, 2021; Kochendoerfer *et al*, 2021). Asymmetric inheritance of old versus new centromere-specific histone variant CENP-A/CID has also been reported in the *Drosophila* ISCs (Garcia Del Arco *et al*, 2018). More studies in the future are needed to understand how global (i.e., in fly male GSCs and ISCs) and local (i.e., in fly female GSCs and mESCs) canonical histone asymmetries are established, which is likely by developmentally programmed DNA replication. Furthermore, how canonical histone asymmetries on sister chromatids are recognized and differentially inherited is likely due to the differential attachment of the mitotic spindle to asymmetric sister centromeres. Finally, the distinct distribution of epigenetic information between the two daughter cells derived from ACD probably prepares them for distinct cellular behaviors, which is crucial to development, tissue homeostasis, and regeneration of multicellular organisms.

It has been debated whether the two cells resulting from ISC division are intrinsically asymmetric, or only become asymmetric through the extrinsic signaling cues after ISC division. Previous studies demonstrate that intrinsic polarity mechanisms result in the asymmetric distribution and inheritance of Par proteins to the apical daughter cell during ACD of ISCs, in order to promote differentiation (Goulas *et al*, 2012). Furthermore, differential Notch activities due to the polarized Par complex induce distinct cellular differentiation pathways (Guo & Ohlstein, 2015). Recent work has also shown that the spindle orientation in ISCs is tightly linked with cell fate, where planar orientation gives rise to two ISCs and angular orientation generates the ISC/EB pair of daughter cells (Hu & Jasper, 2019). Here, through analyzing different mitotic stages of ISCs, separable old versus new H3 distribution is detectable in prophase and prometaphase ISCs regardless of the ISC division modes, indicating that this asymmetry is likely intrinsically established prior to mitosis (Fig 7A). Interestingly, old versus new H3T3A signals are still separable in prophase and prometaphase ISCs, similar to that of wild-type H3, suggesting that this mutant histone does not interfere with differential histone incorporation before mitosis (Fig 4A and B). However, increased symmetric segregation patterns in anaphase and telophase H3T3A-expressing ISCs indicate that sister chromatids differentially enriched with old versus new H3T3A signals cannot be properly recognized and segregated (Fig 4C and D). Because flies have two major autosomes (2nd and 3rd chromosomes) in addition to the sex chromosomes, even the randomized segregation of sister chromatids could lead to an asymmetric pattern at a low percentage, as shown previously (Xie *et al*, 2015). However, even though rare asymmetric histone inheritance occurs with randomized segregation pattern, the daughter cell enriched with old histone is still the cell expressing a higher level of Delta, indicative of ISC identity (Figs 4F and G, and 7C). Together, these findings indicate that ISC cell fate is likely specified by the intrinsic epigenetic information and the polarized extrinsic cues possibly act to ensure their differential segregation pattern. In summary, given the unique features of the ISC system, such as the ability to precisely label each derivative cell in the entire lineage, the large number of ISCs in their endogenous niche, and the sensitivity of ISC activity to environmental changes such as nutrition as well as

aging (Shim *et al*, 2013; Gervais & Bardin, 2017; Jasper, 2020; Rodriguez-Fernandez *et al*, 2020), it will become a new *in vivo* model system to study the fundamental principles of different histone inheritance modes and relevant biological consequences under physiological and pathological conditions.

Materials and Methods

Generation of switchable dual-color transgenes

Standard procedures were used for all molecular cloning experiments. Enzymes used for plasmid construction were obtained from New England Biolabs (Beverly, MA). The new histone sequences, such as *histone-mCherry*, were recovered as an XbaI-flanked fragment and were subsequently inserted into the XbaI site of the UASp plasmid. The old histone sequences, such as *histone-eGFP*, were inserted to *pBluescript-FRT-NheI-SV40 PolyA-FRT* plasmid at the unique NheI site. The entire *NotI-FRT-histone-eGFP-SV40 PolyA-FRT-EcoRI* sequences were then subcloned into the *UASp-histone-mCherry* plasmid digested by *NotI* and *EcoRI*. The final *UASp-FRT-histone-eGFP-PolyA-FRT-histone-mCherry-PolyA* plasmids were introduced to w^{1118} flies by P-element-mediated germline transformation (Bestgene, Inc.). The dual-color H3, H4, and H2A transgenic lines were used previously (Wooten *et al*, 2019; Kahney *et al*, 2021). Other new histone transgenic strains generated for this work are described as follows and are all on either the second or the third chromosome as a single-copy transgene.

Generation of co-expressing H3 transgene

The T2A sequences: gag ggc cgc ggc agc ctg ctg acc tgc ggc gat gtg gag gag aac ccc ggg ccc that encodes the following amino acid sequence: EGRGSLLTCGDVEENPGP was inserted in the *UASp-H3-eGFP* plasmid to generate the *UASp-H3-eGFP-T2A* plasmid, the *H3-mCherry* sequences were subsequently inserted as a XbaI fragment to generate the *UASp-H3-eGFP-T2A-H3-mCherry-PolyA* plasmid, which was introduced to w^{1118} flies by P-element-mediated germline transformation (Bestgene, Inc.).

Generation of H3-mCherry and H3T3A-mCherry transgenes

The histone sequences, such as *H3-mCherry* and *H3T3A-mCherry*, were recovered as an XbaI-flanked fragment and were subsequently inserted into the XbaI site of the UASp to generate: *UASp-H3-mCherry* and *UASp-H3T3A-mCherry* plasmids, which were introduced to w^{1118} flies by P-element-mediated germline transformation (Bestgene, Inc.).

Fly strains and husbandry

Fly stocks were raised using standard Bloomington medium at 25°C unless otherwise noted. Female flies were analyzed at 10-day posteclosion unless otherwise noted. The following fly stocks were used: *hs-flp* on the X chromosome (Bloomington Stock Center BL-26902), *esg-Gal4* on the 2nd chromosome (Dr. Allan Spradling, Carnegie Institute, Baltimore, MD), *esg-Gal4*, *UAS-eYFP* on the 2nd chromosome (Dr. Heinrich Jasper, Buck Institute, Novato, CA), *Su(H)-Gal80*, *tub-Gal80ts* on the 3rd chromosome (Dr. Heinrich Jasper, Buck Institute, Novato, CA), *Su(H)-Gal80* on the 3rd chromosome (separated from the recombined *Su(H)-Gal80*, *tub-Gal80^{ts}*), *Delta-nuclear lacZ* reporter on the 3rd chromosome (Dr. Allan Spradling, Carnegie Institute, Baltimore, MD), *Suppressor-of-Hairless lacZ* reporter on the X chromosome (Dr. Allan Spradling, Carnegie Institute, Baltimore, MD), *UASp-FRT-H3-eGFP-PolyA-FRT-H3-mCherry* on the 2nd chromosome (Wooten *et al*, 2019), *UASp-H3-eGFP-T2A-H3-mCherry* on the 2nd chromosome (this study), *UASp-FRT-H4-eGFP-PolyA-FRT-H4-mCherry* on the 2nd chromosome (this study), *UASp-FRT-H2A-eGFP-PolyA-FRT-H2A-mCherry* on the 3rd chromosome (Wooten *et al*, 2019; Kahney *et al*, 2021), *UASp-FRT-H3T3A-GFP-PolyA-FRT-H3T3A-mKO* on the 3rd chromosome (Xie *et al*, 2015), *UASp-H3-mCherry* on the 2nd chromosome (this study), and *UASp-H3T3A-mCherry* on the 2nd chromosome (this study).

Heat-shock scheme

Flies with *UASp* dual-color histone transgenes were paired with the *esg-Gal4* driver alone (Figs 1–4), or the ISC-specific combination of *esg-Gal4*, *Su(H)-Gal80* driver (Fig 3M and N, Fig EV2B). Flies were raised at 25°C throughout development until adulthood. At eclosion, flies were transferred to new vials and aged for 10 days. On the 10th day, flies were transferred to new vials. Vials were then submerged underneath the water up to the plug in a circulating 37°C water bath for 90 min and were then recovered at 29°C for 18 h before dissection for immunostaining experiments.

Spatiotemporally controlled experiments

Fly crosses that aimed to combine the histone transgenes of interest with the *esg-Gal4*, *Su(H)-Gal80*, *tub-Gal80^{ts}* driver were crossed at the permissive temperature of 18°C to prevent the expression of all transgenes (Fig 5). The F1 generation was kept at 18°C through development until 1–3-day posteclosion. At this point, the flies were shifted to the nonpermissive 29°C to inactivate Gal80^{ts}, allowing for the activation of the *esg-Gal4*, *Su(H)-Gal80* driving expression of the histone transgenes and the eYFP reporter. Flies were then dissected 12 days after the temperature shift and were analyzed via immunostaining.

Immunostaining experiments

Immunofluorescence staining was performed using standard procedures (Tran *et al*, 2012; Marianes & Spradling, 2013). For immunofluorescence staining, midguts from 10-day-old female flies were dissected in Schneider's insect media. For samples that required nucleoside analog incorporation, 10 μM EdU analog (Invitrogen Click-iT EdU Imaging Kit, catalog #C10340) was added and incubated for 30 min. All samples were then transferred to a 4% formaldehyde in phosphate-buffered saline (1 × PBS) for 1 h at room temperature for fixation. Samples were washed three times, 5 min each time in PBST (1 × PBS with 0.1% Triton X-100), and then placed in a solution containing primary antibodies at desired concentrations in 5% normal goat serum (NGS) in PBST. Samples were incubated for a minimum of 24 h at 4°C in primary antibodies. Antibodies used in these experiments are mouse anti-H3S10ph (1:1,000, Abcam AB-14995), mouse anti-prospero (1:100, DSHB MR1A) and

 © 2023 The Authors

chicken anti-ß-galactosidase (1:1,000, Abcam AB-9361). All GFP, mCherry, and mKO fluorescent signals were visualized without using any antibody. Samples were then washed three times, 5 min each time in PBST, and then incubated in a 1:1,000 dilution of Alexa-Flour-conjugated secondary antibody in 5% NGS in PBST for 2 h at room temperature. For EdU visualization, EdU analog was conjugated to Alexa-647 dye using CLICK chemistry [reviewed in (Kolb *et al*, 2001; Nwe & Brechbiel, 2009)]. Samples were washed three times, 5 min each time in PBST, and then mounted for microscopy in Vectashield antifade mounting medium (Vector Laboratories, Cat#H-1400) with or without DAPI. Samples were imaged using a Leica SP8 confocal microscope with a 63× oil immersion objective. Images were analyzed using ImageJ software.

Western blot procedure

Five intestines were dissected in cold PBS and transferred into RIPA buffer for tissue digestion (Boston BioProducts, Cat#BP-115). The sample was then sonicated for three cycles of 30 s on and 1 min off using the Diagenode Bioruptor. The sample was then centrifuged for 6 min at 16,632 *g* to clear debris. The supernatant was transferred to a new tube with 2× Novex Tricine SDS Sample Buffer (Invitrogen, Cat#LC1676) and boiled for 10 min at 95°C. The sample along with the protein standard (Invitrogen Novex Sharp Pre-stained Protein Standard, Cat#LC5800) was loaded into a 10–20% Tricine Gradient Gel (Invitrogen Novex, Cat#EC66252BOX) and run in 1× Novex Tricine SDS Running Buffer (Invitrogen, Cat#LC1675) for approximately 90 min at 120 V using the XCell SureLock Mini-Cell. The protein was then transferred onto a nitrocellulose membrane (Invitrogen, Cat #LC2000) using the XCell II Blot Module, transferring at 4°C for 90 min at 30 V in 1X Tris-Glycine Transfer Buffer with 20% methanol (Invitrogen, Cat#LC3675). The membrane was then placed in a blocking solution of 5% BSA (Cell Signaling, #9998S) in 1× TBST (10× Tris Buffered Saline with 1% Tween-20, Boston BioProducts, Inc., Cat#IBB-180). The membrane was then incubated with the primary antibody (rabbit anti-GFP, Abcam AB 290, 1:1,000) diluted in the blocking solution at 4°C overnight. The membrane was then washed three times for 10 min in 1× TBST and was then incubated in the secondary antibody (HRP-conjugated goat anti-rabbit, Cell Signaling #7074) diluted in blocking solution for 2 h at room temperature. After this incubation, the membrane was washed again three times for 10 min in 1× TBST and was then incubated with ECL solution (Abcam, AB 133406) for 2 min for visualization. The membrane was visualized using the GeneSys image acquisition software on a G:Box Chemi XX6 system.

Correlation analysis between old and new histones in prophase and prometaphase cells

Prophase and prometaphase cells were identified based on the mitotic marker H3S10ph and chromosomal morphology. The center of this cell was identified and outlined as a region of interest, and the image was split into two channels, old histone (eGFP at 488 nm) and new histone (mCherry at 568 nm), for image analysis. The Coloc2 analysis tool in ImageJ was used to collect the Pearson and Spearman correlation coefficients. The old and new histone channels were compared, and the Pearson and Spearman correlation coefficients were recorded and compared among different samples, similar to the analyses performed in Kahney *et al* (2021).

Correlation analysis between eYFP and Su(H)-LacZ in H3- and H3T3A-expressing intestines

Regions measuring 150 μm by 150 μm were used for the colocalization analysis between eYFP and Su(H)-LacZ signals. Each intestine was used to retrieve seven unique regions for analysis. The region was split into the two channels for image analysis using the Coloc2 analysis tool in ImageJ, which provided the Pearson's correlation coefficient, which were recorded and compared between H3- and H3T3A-expressing intestines.

Quantification of fluorescent signals

For all quantification, GFP, mCherry, and mKO fluorescent signals were not enhanced with antibody, only the endogenous fluorescence was used. Values of GFP, mCherry, mKO, and Dl-nLacZ were measured using ImageJ software. The cell or region of interest was outlined, and the raw integrated density was recorded for each channel of interest. A background measurement was then taken using the same outlined region that was measured in an area with no fluorescence signal. The background measurement was subtracted from the initial measurement. For every cell, each z slice containing that cell was included for quantification, and the values were added together to get the total value. These values were then used to compute the ratios of interest, similar to the analyses performed in Ranjan *et al* (2019, 2022).

Quantification of histone signals in anaphase and telophase ISCs

Anaphase and telophase cells were identified based on the mitotic marker H3S10ph and chromosomal morphology. The sister chromatids need to be segregated to show clear separation, distinguishing the two individual sets of sister chromatids. Each set of sister chromatids was outlined, and old histone was measured using the method described above for the total signals associated with each set. A ratio was then computed by dividing the sister chromatids with the higher value of old histone by sister chromatids with the lower value of old histone.

Quantification of histone signals in postmitotic pairs

To quantify the ratios of old and new histones in postmitotic pairs, the pairs were first selected using the following criteria. The two cell nuclei must be within 5 μm of each other, one cell must be no more than 1.5× larger than the other cell based on cell diameter to avoid using the polyploid enterocytes (EC), both cells must be EdU negative, and both cells must contain quantifiable levels of old and new histone. Quantification of old histone, new histone, and Dl-nLacZ was conducted using the method described above for each cell. The cell with a higher Dl-nLacZ value was denoted as cell 1, and the other as cell 2. A ratio was computed by dividing the values of old histone, new histone, and Dl-nLacZ for cell 1 by cell 2. Any pair with a Dl-nLacZ ratio greater than 2 was considered an asymmetric pair, while any pair with a ratio less than 2 was considered a

symmetric pair. The ratios for old and new histone were then recorded as a ratio of cell 1/cell 2 for Delta-asymmetric and Delta-symmetric pairs.

Quantification of UAS-eYFP signals along the profile of an intestine

Intestines were stitched together using the stitching tool in ImageJ to show entire intestine profile in the eYFP channel. The image was then made into a maximum projection, flattening all z slices into one image. The line tool was then used at a width of 500 pixels (approximately the width of the average intestine), and a line was drawn along the entire profile of the intestine. From there, the plot profile analysis was conducted in ImageJ, providing the average intensity value of eYFP signals across the 500 pixel width at each pixel along the intestinal profile (approximately 10,000–20,000 measurements per intestine). These measurements were then used to provide summary values for each intestine. Every 1,000-pixel measurements were averaged to provide one value, giving 10–20 values per intestine. These averaged values were then combined from at least 10 individual intestines into a single dataset.

Quantification of differentiated cell types in H3- and H3T3A-expressing intestines

Regions measuring 150 μm by 150 μm were used for this analysis. Each intestine was used to retrieve seven unique regions for analysis. The cell counter tool in ImageJ was used to count the number of ee cells, using Prospero staining, or ECs cells, using DAPI to identify the polyploid cells, per region. These quantifications were recorded and used for comparison of the number of differentiated cells per region between H3- and H3T3A-expressing intestines.

Single-cell RNA sequencing

H3- and H3T3A-expressing intestines, driven by the *esg-Gal4*, *Su(H)-Gal80*, *tub-Gal80^{ts}* driver, were grown at the nonpermissive 29°C for 12–14 days before sample preparation. Fifteen intestines of both H3 and H3T3A were dissected for the preparation of a single-cell suspension. Intestines were transferred into a digestion buffer of 2 mg/ml collagenase with TrypLE and the tissues were digested in a water bath at 37°C for 15 min, with short vortexes every 2 min. Cells were then filtered with a 40 μm filter and then pelleted using a centrifuge. They were then washed with HBSS and resuspended in 20 μl of HBSS to get the single-cell suspension. Cells were counted using a hemocytometer and diluted as needed to continue on with the single-cell RNA-sequencing protocol. From here, the 10× Genomics protocol was followed to conduct single-cell RNA sequencing.

Single-cell RNA-seq intake

CellRanger feature matrices were loaded into Seurat 4.3.0 (Satija *et al*, 2015), and preprocessing was done via Seurat's SCTransform with the current-generation option vst.flavor = "v2" (sctransform 0.3.5; Hafemeister & Satija, 2019). We used Seurat's elbow plot to determine the number of principal components (1:6) to use for H3 and H3T3A. This is used for Seurat's FindClusters (Leiden algorithm), where we set a coarse resolution parameter of 0.1 since we do not need to identify cell subtypes. The H3 sample was relatively overloaded with cells, relative to the H3T3A sample, leading to lower feature counts per cell in the H3 sample. However, there was one esg$^+$ stem cell cluster with good quantification levels in both samples. Our filter (independently over each sample) consists of choosing the cluster with highest esg AverageExpression, taking the median feature count of cells in cluster, then applying this minimum feature count to all cells and re-PCA and reclustering.

Next, one or more clusters were annotated with "ISC/EB," "EE," "aEC," "mEC," "LFC," "pEC," or "unknown" levels by testing marker gene levels in the SCTransform-corrected data. UMAP shows all cells, while only the "ISC/EB" level was retained for downstream analysis. In particular, expression of ISC/EB markers is shown in a zoomed-in UMAP plot, selecting the esg$^+$ cell population which was labeled as "ISC/EB."

Alignment and regression of single-cell RNA-seq pseudotime

The SCTransform-corrected, normalized gene expression data may be used, on the ISC/EB level, for Slingshot pseudotime (Street *et al*, 2018). The pseudotime variable is a one-dimensional dimensionality reduction using the principle of minimum spanning tree (MST) to localize cell subpopulations. We used the default Slingshot parameters, retaining Seurat's principal components (1:50).

The Slingshot models from H3 and H3T3A samples are aligned by sorting the cell embeddings matrix according to the Slingshot variable, applying a kernel smoother, and dynamic time warping (dtw of the smoothed PCA features; default parameters; Alpert *et al*, 2018). The output of dtw is a time series starting from (1, 1), where from the first and/or second array, the array index may either be incremented by one or not incremented, until we reach (m, n), the final value.

Alignment of trajectories allows H3 and H3T3A transcriptome features to be plotted side-by-side. We applied lighter smoothing for visualization and then tiled an *x*-axis of aligned cells (H3 and H3T3A) and *y*-axis of genes. Genes were selected over a pseudotime model using the tradeSeq library (Van den Berge *et al*, 2020), with a generalized additive model and association test for significance, and then set union of significant genes between H3 and H3T3A. Among these genes, we could search for early-stage markers performing similarly to Delta, as well as late-stage markers which are mutually exclusive with Delta.

We applied differential expression (regression) to pseudotime, to identify whether a marker gene's levels will increase or decrease log-linearly across pseudotime. We may apply the DE library glmGamPoi (Ahlmann-Eltze & Huber, 2021) to the two 10× samples, such that it fits a model for one gene with four coefficients (consisting of two lines with slope and intercept). As recommended by glmGamPoi, the size factors (library sizes) of cell barcodes come from the scran library applied to the samples (Lun *et al*, 2016). To account for pseudotime values being mis-scaled between different samples, we applied R's scale function and then took the mean pseudotime coordinate between samples at each step in dtw. This variable will place the two cells at the same point along the *x*-axis. glmGamPoi provides a coefficient *pseudotime:conditionH3T3A*, which stores the difference between the H3T3A pseudotime slope and the H3 pseudotime slope, and which we test against a null

© 2023 The Authors

hypothesis of zero coefficient. We chose this model due to its robust linear nature, although it has limited applicability, because other experiments will have several cell types in pseudotime, with marker genes rising and falling (nonlinear) and lacking in statistical power.

To improve the utility of this model, where the slope of the regression line for the H3 wild-type Delta levels was originally small, we considered means to remove outlier cells. We applied the scType cell type classification library (Ianevski *et al*, 2022) for outlier detection, using an "ISC" cell type and "EB" cell type, choosing one associated marker gene for each. These cell types appear closely overlapping in our plots, so we are unsure of the goodness of fit of the scType classifier to identify ISC or EB cells, but it does produce an "Unknown" class for subpopulations (using, e.g., hierarchical clustering), which may be negative for all provided marker genes. Therefore, cells with an scType label of "Unknown" were rejected as outliers before applying the regression model, but the "ISC" and "EB" predicted cell type labels were discarded. This is validated by most cells having a clear Dl^{+} klu^{-} or Dl^{-} klu^{+} transcriptome profile (Fig 6G and H).

Statistics and reproducibility

Data were subjected to the Shapiro–Wilk test to determine whether the data were normally distributed or skewed. For normally distributed data, a single-sample *t*-test was used for comparing one dataset to a hypothesized mean of 0 or 1. For skewed data, the Wilcoxon signed-rank test was used to compare one dataset to a hypothesized median of $\log_2 = 0$ or 1:1 ratio. An unpaired two-sample *t*-test was used to compare two individual datasets to each other. A chi-square test was used to compare distributions of datasets containing data from different categories. Data are presented with error bars representing the mean ± SE (standard error). Significant differences based on these statistical analyses were noted by asterisks (*$P < 0.05$, **$P < 0.01$, ***$P < 0.001$. ****$P < 0.0001$).

Single-cell RNA-seq data are analyzed using Gamma-Poisson regression. We selected an initial list of two potential marker genes (*Delta*, *Klumpfuss*) before running differential expression or regression, so multiple hypothesis correction was not necessary. Our null hypothesis is that the interaction term (coefficient) *pseudotime:conditionH3T3A*, the difference in slope of two lines, is zero. For each gene, a *P*-value is presented from the likelihood ratio test, where the regression model is evaluated against a reduced model with this term removed.

Data availability

GEO accession number for the single-cell RNA-seq (sc RNA-seq) data is GSE228292 (URL: https://www.ncbi.nlm.nih.gov/geo/query/acc.cgi?acc=GSE228292). Our analysis of the sc RNA-seq data is available (URL: https://github.com/ringw/Drosophila-Intestinal-Stem-Cell-Divisions-2023).

Expanded View for this article is available online.

Acknowledgements

We thank Drs. A. Spradling, W. Ludington, and the Chen laboratory members for their critical comments and suggestions on this work. This work has been supported by NIH T32GM007231 and NIH F31DK122702 (EHZ), F31GM122339 (EWK), NIGMS/NIH R35GM127075, and the HHMI Faculty Scholarship and Investigator program from Howard Hughes Medical Institute (XC).

Author contributions

Emily H Zion: Conceptualization; formal analysis; funding acquisition; validation; investigation; visualization; methodology; writing – original draft; writing – review and editing. **Daniel Ringwalt:** Data curation; software; formal analysis; methodology; writing – review and editing. **Kristina Rinaldi:** Investigation; visualization; methodology. **Elizabeth W Kahney:** Visualization; methodology. **Yingying Li:** Validation; methodology. **Xin Chen:** Conceptualization; supervision; funding acquisition; writing – original draft; project administration; writing – review and editing.

Disclosure and competing interests statement

The authors declare that they have no conflict of interest.

References

Ahlmann-Eltze C, Huber W (2021) glmGamPoi: fitting gamma-Poisson generalized linear models on single cell count data. *Bioinformatics* 36: 5701–5702

Ahmad K, Henikoff S (2002a) Histone H3 variants specify modes of chromatin assembly. *Proc Natl Acad Sci USA* 99: 16477–16484

Ahmad K, Henikoff S (2002b) The histone variant H3.3 marks active chromatin by replication-independent nucleosome assembly. *Mol Cell* 9: 1191–1200

Ahmad K, Henikoff S (2018) No strand left behind. *Science* 361: 1311–1312

Ahmed SMH, Maldera JA, Krunic D, Paiva-Silva GO, Penalva C, Teleman AA, Edgar BA (2020) Fitness trade-offs incurred by ovary-to-gut steroid signalling in *Drosophila*. *Nature* 584: 415–419

Alabert C, Barth TK, Reveron-Gomez N, Sidoli S, Schmidt A, Jensen ON, Imhof A, Groth A (2015) Two distinct modes for propagation of histone PTMs across the cell cycle. *Genes Dev* 29: 585–590

Allis CD, Jenuwein T (2016) The molecular hallmarks of epigenetic control. *Nat Rev Genet* 17: 487–500

Alpert A, Moore LS, Dubovik T, Shen-Orr SS (2018) Alignment of single-cell trajectories to compare cellular expression dynamics. *Nat Methods* 15: 267–270

Amoyel M, Simons BD, Bach EA (2014) Neutral competition of stem cells is skewed by proliferative changes downstream of Hh and Hpo. *EMBO J* 33: 2295–2313

Antel M, Raj R, Masoud MYG, Pan Z, Li S, Mellone BG, Inaba M (2022) Interchromosomal interaction of homologous Stat92E alleles regulates transcriptional switch during stem-cell differentiation. *Nat Commun* 13: 3981

Badeaux AI, Shi Y (2013) Emerging roles for chromatin as a signal integration and storage platform. *Nat Rev Mol Cell Biol* 14: 211–224

Bai L, Morozov AV (2010) Gene regulation by nucleosome positioning. *Trends Genet* 26: 476–483

Bannister AJ, Kouzarides T (2011) Regulation of chromatin by histone modifications. *Cell Res* 21: 381–395

Bardin AJ, Perdigoto CN, Southall TD, Brand AH, Schweisguth F (2010) Transcriptional control of stem cell maintenance in the *Drosophila* intestine. *Development* 137: 705–714

Beebe K, Lee WC, Micchelli CA (2010) JAK/STAT signaling coordinates stem cell proliferation and multilineage differentiation in the *Drosophila* intestinal stem cell lineage. *Dev Biol* 338: 28–37

© 2023 The Authors

Carty BL, Dunleavy EM (2020) Centromere assembly and non-random sister chromatid segregation in stem cells. *Essays Biochem* 64: 223–232

Carty BL, Dattoli AA, Dunleavy EM (2021) CENP-C functions in centromere assembly, the maintenance of CENP-A asymmetry and epigenetic age in *Drosophila* germline stem cells. *PLoS Genet* 17: e1009247

Chen J, Xu N, Wang C, Huang P, Huang H, Jin Z, Yu Z, Cai T, Jiao R, Xi R (2018) Transient Scute activation via a self-stimulatory loop directs enteroendocrine cell pair specification from self-renewing intestinal stem cells. *Nat Cell Biol* 20: 152–161

Chereji RV, Ramachandran S, Bryson TD, Henikoff S (2018) Precise genome-wide mapping of single nucleosomes and linkers in vivo. *Genome Biol* 19: 19

Clevers H (2005) Stem cells, asymmetric division and cancer. *Nat Genet* 37: 1027–1028

Dattoli AA, Carty BL, Kochendoerfer AM, Morgan C, Walshe AE, Dunleavy EM (2020) Asymmetric assembly of centromeres epigenetically regulates stem cell fate. *J Cell Biol* 219: e201910084

de Cuevas M, Matunis EL (2011) The stem cell niche: lessons from the *Drosophila* testis. *Development* 138: 2861–2869

de Navascues J, Perdigoto CN, Bian Y, Schneider MH, Bardin AJ, Martinez-Arias A, Simons BD (2012) *Drosophila* midgut homeostasis involves neutral competition between symmetrically dividing intestinal stem cells. *EMBO J* 31: 2473–2485

Doupe DP, Marshall OJ, Dayton H, Brand AH, Perrimon N (2018) *Drosophila* intestinal stem and progenitor cells are major sources and regulators of homeostatic niche signals. *Proc Natl Acad Sci USA* 115: 12218–12223

Dunleavy EM, Beier NL, Gorgescu W, Tang J, Costes SV, Karpen GH (2012) The cell cycle timing of centromeric chromatin assembly in *Drosophila* meiosis is distinct from mitosis yet requires CAL1 and CENP-C. *PLoS Biol* 10: e1001460

Escobar TM, Loyola A, Reinberg D (2021) Parental nucleosome segregation and the inheritance of cellular identity. *Nat Rev Genet* 22: 379–392

Garcia Del Arco A, Edgar BA, Erhardt S (2018) In vivo analysis of Centromeric proteins reveals a stem cell-specific asymmetry and an essential role in differentiated, non-proliferating cells. *Cell Rep* 22: 1982–1993

Gervais L, Bardin AJ (2017) Tissue homeostasis and aging: new insight from the fly intestine. *Curr Opin Cell Biol* 48: 97–105

Goldberg AD, Banaszynski LA, Noh KM, Lewis PW, Elsaesser SJ, Stadler S, Dewell S, Law M, Guo X, Li X *et al* (2010) Distinct factors control histone variant H3.3 localization at specific genomic regions. *Cell* 140: 678–691

Goulas S, Conder R, Knoblich JA (2012) The par complex and integrins direct asymmetric cell division in adult intestinal stem cells. *Cell Stem Cell* 11: 529–540

Guo Z, Ohlstein B (2015) Stem cell regulation. Bidirectional notch signaling regulates *Drosophila* intestinal stem cell multipotency. *Science* 350: aab0988

Hafemeister C, Satija R (2019) Normalization and variance stabilization of single-cell RNA-seq data using regularized negative binomial regression. *Genome Biol* 20: 296

Hendzel MJ, Wei Y, Mancini MA, Van Hooser A, Ranalli T, Brinkley BR, Bazett-Jones DP, Allis CD (1997) Mitosis-specific phosphorylation of histone H3 initiates primarily within pericentromeric heterochromatin during G2 and spreads in an ordered fashion coincident with mitotic chromosome condensation. *Chromosoma* 106: 348–360

Hu DJ, Jasper H (2019) Control of intestinal cell fate by dynamic mitotic spindle repositioning influences epithelial homeostasis and longevity. *Cell Rep* 28: e2805

Huang H, Sabari BR, Garcia BA, Allis CD, Zhao Y (2014) SnapShot: histone modifications. *Cell* 159: 458

Hung RJ, Hu Y, Kirchner R, Liu Y, Xu C, Comjean A, Tattikota SG, Li F, Song W, Ho Sui S *et al* (2020) A cell atlas of the adult drosophila midgut. *Proc Natl Acad Sci USA* 117: 1514–1523

Ianevski A, Giri AK, Aittokallio T (2022) Fully-automated and ultra-fast cell-type identification using specific marker combinations from single-cell transcriptomic data. *Nat Commun* 13: 1246

Jackson V (1988) Deposition of newly synthesized histones: hybrid nucleosomes are not tandemly arranged on daughter DNA strands. *Biochemistry* 27: 2109–2120

Jackson V, Chalkley R (1981) A new method for the isolation of replicative chromatin: selective deposition of histone on both new and old DNA. *Cell* 23: 121–134

Jasper H (2020) Intestinal stem cell aging: origins and interventions. *Annu Rev Physiol* 82: 203–226

Jiang C, Pugh BF (2009) Nucleosome positioning and gene regulation: advances through genomics. *Nat Rev Genet* 10: 161–172

Jiang H, Patel PH, Kohlmaier A, Grenley MO, McEwen DG, Edgar BA (2009) Cytokine/Jak/Stat signaling mediates regeneration and homeostasis in the drosophila midgut. *Cell* 137: 1343–1355

Jiang H, Grenley MO, Bravo MJ, Blumhagen RZ, Edgar BA (2011) EGFR/Ras/MAPK signaling mediates adult midgut epithelial homeostasis and regeneration in *Drosophila*. *Cell Stem Cell* 8: 84–95

Jin Y, Patel PH, Kohlmaier A, Pavlovic B, Zhang C, Edgar BA (2017) Intestinal stem cell Pool regulation in *Drosophila*. *Stem Cell Reports* 8: 1479–1487

Kahney EW, Ranjan R, Gleason RJ, Chen X (2017) Symmetry from asymmetry or asymmetry from symmetry? *Cold Spring Harb Symp Quant Biol* 82: 305–318

Kahney EW, Snedeker JC, Chen X (2019) Regulation of *Drosophila* germline stem cells. *Curr Opin Cell Biol* 60: 27–35

Kahney EW, Zion EH, Sohn L, Viets-Layng K, Johnston R, Chen X (2021) Characterization of histone inheritance patterns in the drosophila female germline. *EMBO Rep* 22: e51530

Katan-Khaykovich Y, Struhl K (2011) Splitting of H3-H4 tetramers at transcriptionally active genes undergoing dynamic histone exchange. *Proc Natl Acad Sci USA* 108: 1296–1301

Kiger AA, Jones DL, Schulz C, Rogers MB, Fuller MT (2001) Stem cell self-renewal specified by JAK-STAT activation in response to a support cell cue. *Science* 294: 2542–2545

Knoblich JA (2008) Mechanisms of asymmetric stem cell division. *Cell* 132: 583–597

Knoblich JA (2010) Asymmetric cell division: recent developments and their implications for tumour biology. *Nat Rev Mol Cell Biol* 11: 849–860

Kochendoerfer AM, Modafferi F, Dunleavy EM (2021) Centromere function in asymmetric cell division in *Drosophila* female and male germline stem cells. *Open Biol* 11: 210107

Kochendoerfer AM, Keegan RS, Dunleavy EM (2023) Centromere proteins are asymmetrically distributed between newly divided germline stem and daughter cells and maintain a balanced niche in *Drosophila* males. *Mol Biol Cell* 34: ar42

Kolb HC, Finn MG, Sharpless KB (2001) Click chemistry: diverse chemical function from a few good reactions. *Angew Chem Int Ed Engl* 40: 2004–2021

Kornberg RD (1974) Chromatin structure: a repeating unit of histones and DNA. *Science* 184: 868–871

Kornberg RD, Lorch Y (2020) Primary role of the nucleosome. *Mol Cell* 79: 371–375

 © 2023 The Authors

Korzelius J, Azami S, Ronnen-Oron T, Koch P, Baldauf M, Meier E, Rodriguez-Fernandez IA, Groth M, Sousa-Victor P, Jasper H (2019) The WT1-like transcription factor Klumpfuss maintains lineage commitment of enterocyte progenitors in the *Drosophila* intestine. *Nat Commun* 10: 4123

Kouzarides T (2007) Chromatin modifications and their function. *Cell* 128: 693 – 705

Lai B, Gao W, Cui K, Xie W, Tang Q, Jin W, Hu G, Ni B, Zhao K (2018) Principles of nucleosome organization revealed by single-cell micrococcal nuclease sequencing. *Nature* 562: 281 – 285

Leatherman JL, Dinardo S (2008) Zfh-1 controls somatic stem cell self-renewal in the *Drosophila* testis and nonautonomously influences germline stem cell self-renewal. *Cell Stem Cell* 3: 44 – 54

Lewis PW, Elsaesser SJ, Noh KM, Stadler SC, Allis CD (2010) Daxx is an H3.3-specific histone chaperone and cooperates with ATRX in replication-independent chromatin assembly at telomeres. *Proc Natl Acad Sci USA* 107: 14075 – 14080

Li Y, Ma Q, Cherry CM, Matunis EL (2014) Steroid signaling promotes stem cell maintenance in the *Drosophila* testis. *Dev Biol* 394: 129 – 141

Lin G, Xu N, Xi R (2008) Paracrine wingless signalling controls self-renewal of *Drosophila* intestinal stem cells. *Nature* 455: 1119 – 1123

Lin S, Yuan ZF, Han Y, Marchione DM, Garcia BA (2016) Preferential phosphorylation on old histones during early mitosis in human cells. *J Biol Chem* 291: 15342 – 15357

Losick VP, Morris LX, Fox DT, Spradling A (2011) *Drosophila* stem cell niches: a decade of discovery suggests a unified view of stem cell regulation. *Dev Cell* 21: 159 – 171

Loza-Coll MA, Southall TD, Sandall SL, Brand AH, Jones DL (2014) Regulation of *Drosophila* intestinal stem cell maintenance and differentiation by the transcription factor escargot. *EMBO J* 33: 2983 – 2996

Luger K, Mader AW, Richmond RK, Sargent DF, Richmond TJ (1997) Crystal structure of the nucleosome core particle at 2.8 a resolution. *Nature* 389: 251 – 260

Lun AT, McCarthy DJ, Marioni JC (2016) A step-by-step workflow for low-level analysis of single-cell RNA-seq data with Bioconductor. *F1000Res* 5: 2122

Ma B, Trieu TJ, Cheng J, Zhou S, Tang Q, Xie J, Liu JL, Zhao K, Habib SJ, Chen X (2020) Differential histone distribution patterns in induced asymmetrically dividing mouse embryonic stem cells. *Cell Rep* 32: 108003

Marianes A, Spradling AC (2013) Physiological and stem cell compartmentalization within the *Drosophila* midgut. *Elife* 2: e00886

Martin JL, Sanders EN, Moreno-Roman P, Jaramillo Koyama LA, Balachandra S, Du X, O'Brien LE (2018) Long-term live imaging of the *Drosophila* adult midgut reveals real-time dynamics of division, differentiation and loss. *Elife* 7: e36248

Mellone BG, Grive KJ, Shteyn V, Bowers SR, Oderberg I, Karpen GH (2011) Assembly of *Drosophila* centromeric chromatin proteins during mitosis. *PLoS Genet* 7: e1002068

Micchelli CA, Perrimon N (2006) Evidence that stem cells reside in the adult *Drosophila* midgut epithelium. *Nature* 439: 475 – 479

Morrison SJ, Kimble J (2006) Asymmetric and symmetric stem-cell divisions in development and cancer. *Nature* 441: 1068 – 1074

Morrison SJ, Spradling AC (2008) Stem cells and niches: mechanisms that promote stem cell maintenance throughout life. *Cell* 132: 598 – 611

Nacev BA, Feng L, Bagert JD, Lemiesz AE, Gao J, Soshnev AA, Kundra R, Schultz N, Muir TW, Allis CD (2019) The expanding landscape of 'oncohistone' mutations in human cancers. *Nature* 567: 473 – 478

Nwe K, Brechbiel MW (2009) Growing applications of "click chemistry" for bioconjugation in contemporary biomedical research. *Cancer Biother Radiopharm* 24: 289 – 302

Obniski R, Sieber M, Spradling AC (2018) Dietary lipids modulate notch signaling and influence adult intestinal development and metabolism in *Drosophila*. *Dev Cell* 47: 98 – 111

O'Brien LE, Soliman SS, Li X, Bilder D (2011) Altered modes of stem cell division drive adaptive intestinal growth. *Cell* 147: 603 – 614

Ohlstein B, Spradling A (2006) The adult *Drosophila* posterior midgut is maintained by pluripotent stem cells. *Nature* 439: 470 – 474

Ohlstein B, Spradling A (2007) Multipotent *Drosophila* intestinal stem cells specify daughter cell fates by differential notch signaling. *Science* 315: 988 – 992

Patel PH, Dutta D, Edgar BA (2015) Niche appropriation by *Drosophila* intestinal stem cell tumours. *Nat Cell Biol* 17: 1182 – 1192

Ranjan R, Chen X (2022) Mitotic drive in asymmetric epigenetic inheritance. *Biochem Soc Trans* 50: 675 – 688

Ranjan R, Snedeker J, Chen X (2019) Asymmetric centromeres differentially coordinate with mitotic machinery to ensure biased sister chromatid segregation in germline stem cells. *Cell Stem Cell* 25: 666 – 681

Ranjan R, Snedeker J, Wooten M, Chu C, Bracero S, Mouton T, Chen X (2022) Differential condensation of sister chromatids acts with Cdc6 to ensure asynchronous S-phase entry in *Drosophila* male germline stem cell lineage. *Dev Cell* 57: 1102 – 1118

Richmond TJ, Davey CA (2003) The structure of DNA in the nucleosome core. *Nature* 423: 145 – 150

Rodriguez-Fernandez IA, Tauc HM, Jasper H (2020) Hallmarks of aging *Drosophila* intestinal stem cells. *Mech Ageing Dev* 190: 111285

Russev G, Hancock R (1981) Formation of hybrid nucleosomes cantaining new and old histones. *Nucleic Acids Res* 9: 4129 – 4137

Satija R, Farrell JA, Gennert D, Schier AF, Regev A (2015) Spatial reconstruction of single-cell gene expression data. *Nat Biotechnol* 33: 495 – 502

Schuh M, Lehner CF, Heidmann S (2007) Incorporation of *Drosophila* CID/CENP-A and CENP-C into centromeres during early embryonic anaphase. *Curr Biol* 17: 237 – 243

Serra-Cardona A, Zhang Z (2018) Replication-coupled nucleosome assembly in the passage of epigenetic information and cell identity. *Trends Biochem Sci* 43: 136 – 148

Shim J, Gururaja-Rao S, Banerjee U (2013) Nutritional regulation of stem and progenitor cells in *Drosophila*. *Development* 140: 4647 – 4656

Small EC, Xi L, Wang JP, Widom J, Licht JD (2014) Single-cell nucleosome mapping reveals the molecular basis of gene expression heterogeneity. *Proc Natl Acad Sci USA* 111: E2462 – E2471

Snedeker J, Wooten M, Chen X (2017) The inherent asymmetry of DNA replication. *Annu Rev Cell Dev Biol* 33: 291 – 318

Stewart-Morgan KR, Petryk N, Groth A (2020) Chromatin replication and epigenetic cell memory. *Nat Cell Biol* 22: 361 – 371

Stillman B (2018) Histone modifications: insights into their influence on gene expression. *Cell* 175: 6 – 9

Stine RR, Greenspan LJ, Ramachandran KV, Matunis EL (2014) Coordinate regulation of stem cell competition by slit-Robo and JAK-STAT signaling in the *Drosophila* testis. *PLoS Genet* 10: e1004713

Street K, Risso D, Fletcher RB, Das D, Ngai J, Yosef N, Purdom E, Dudoit S (2018) Slingshot: cell lineage and pseudotime inference for single-cell transcriptomics. *BMC Genomics* 19: 477

Sun Z, Tang Y, Zhang Y, Fang Y, Jia J, Zeng W, Fang D (2021) Joint single-cell multiomic analysis in Wnt3a induced asymmetric stem cell division. *Nat Commun* 12: 5941

© 2023 The Authors

Szenker E, Ray-Gallet D, Almouzni G (2011) The double face of the histone variant H3.3. *Cell Res* 21: 421 – 434

Tagami H, Ray-Gallet D, Almouzni G, Nakatani Y (2004) Histone H3.1 and H3.3 complexes mediate nucleosome assembly pathways dependent or independent of DNA synthesis. *Cell* 116: 51 – 61

Tian A, Jiang J (2014) Intestinal epithelium-derived BMP controls stem cell self-renewal in *Drosophila* adult midgut. *Elife* 3: e01857

Tran V, Lim C, Xie J, Chen X (2012) Asymmetric division of *Drosophila* male germline stem cell shows asymmetric histone distribution. *Science* 338: 679 – 682

Tulina N, Matunis E (2001) Control of stem cell self-renewal in *Drosophila* spermatogenesis by JAK-STAT signaling. *Science* 294: 2546 – 2549

Van den Berge K, Roux de Bezieux H, Street K, Saelens W, Cannoodt R, Saeys Y, Dudoit S, Clement L (2020) Trajectory-based differential expression analysis for single-cell sequencing data. *Nat Commun* 11: 1201

Venkei ZG, Yamashita YM (2018) Emerging mechanisms of asymmetric stem cell division. *J Cell Biol* 217: 3785 – 3795

Wang L, Ryoo HD, Qi Y, Jasper H (2015) PERK limits *Drosophila* lifespan by promoting intestinal stem cell proliferation in response to ER stress. *PLoS Genet* 11: e1005220

Wooten M, Snedeker J, Nizami ZF, Yang X, Ranjan R, Urban E, Kim JM, Gall J, Xiao J, Chen X (2019) Asymmetric histone inheritance via strand-specific incorporation and biased replication fork movement. *Nat Struct Mol Biol* 26: 732 – 743

Xie J, Wooten M, Tran V, Chen BC, Pozmanter C, Simbolon C, Betzig E, Chen X (2015) Histone H3 threonine phosphorylation regulates asymmetric histone inheritance in the *Drosophila* male germline. *Cell* 163: 920 – 933

Xie J, Wooten M, Tran V, Chen X (2017) Breaking symmetry – asymmetric histone inheritance in stem cells. *Trends Cell Biol* 27: 527 – 540

Xu M, Zhu B (2010) Nucleosome assembly and epigenetic inheritance. *Protein Cell* 1: 820 – 829

Xu M, Long C, Chen X, Huang C, Chen S, Zhu B (2010) Partitioning of histone H3-H4 tetramers during DNA replication-dependent chromatin assembly. *Science* 328: 94 – 98

Yadav T, Quivy JP, Almouzni G (2018) Chromatin plasticity: a versatile landscape that underlies cell fate and identity. *Science* 361: 1332 – 1336

Young NL, DiMaggio PA, Garcia BA (2010) The significance, development and progress of high-throughput combinatorial histone code analysis. *Cell Mol Life Sci* 67: 3983 – 4000

Zaret KS, Carroll JS (2011) Pioneer transcription factors: establishing competence for gene expression. *Genes Dev* 25: 2227 – 2241

Zeng X, Hou SX (2015) Enteroendocrine cells are generated from stem cells through a distinct progenitor in the adult *Drosophila* posterior midgut. *Development* 142: 644 – 653

Zeng X, Chauhan C, Hou SX (2010) Characterization of midgut stem cell- and enteroblast-specific Gal4 lines in *Drosophila*. *Genesis* 48: 607 – 611

Zhang Y, Sun Z, Jia J, Du T, Zhang N, Tang Y, Fang Y, Fang D (2021) Overview of histone modification. *Adv Exp Med Biol* 1283: 1 – 16

Zion EH, Chandrasekhara C, Chen X (2020) Asymmetric inheritance of epigenetic states in asymmetrically dividing stem cells. *Curr Opin Cell Biol* 67: 27 – 36

License: This is an open access article under the terms of the Creative Commons Attribution License, which permits use, distribution and reproduction in any medium, provided the original work is properly cited.

 © 2023 The Authors

Expanded View Figures

Figure EV1. Related to Figs 1 and 2: Histone transgene expression with the heat-shock-induced genetic switch over time with different histones and the development of a new histone co-expression construct, as well as the comparison of Pearson versus Spearman correlation coefficients in determining the colocalization of GFP-tagged and mCherry- or mKO-tagged histones in prophase and prometaphase ISCs.

A Time course of expression of H3-eGFP and H3-mCherry related to the heat shock. With no heat shock, only H3-eGFP is expressed. 12 h after the heat shock, some H3-mCherry is being expressed. By 24 h, robust expression of both H3-eGFP and H3-mCherry can be observed. After 36 h, H3-mCherry is predominantly observed, with minimal H3-eGFP remaining.

B Expression of H3-eGFP and H3-mCherry compared with H3.3-eGFP and H3.3-mCherry 12 h after heat shock. After 12 h, minimal H3-mCherry has been synthesized and incorporated into chromatin; however, H3.3 shows robust synthesis and incorporation of H3.3-mCherry. This is likely due to mode of incorporation, as H3.3 is incorporated into chromatin in a replication-independent manner, while H3 requires DNA replication for incorporation.

C Quantification of the number of ISCs expressing new H3-mCherry with no heat shock and 18 h after heat shock. Consistent square regions of 150 μm by 150 μm were analyzed in midguts with no heat shock and 18-h post-heat shock. With no heat shock, minimal ISCs expressing H3-mCherry were observed, and at 18 h after heat shock, most ISCs were expressing H3-mCherry (no heat shock, % of ISCs = 2.42 ± 1.39%, n = 25 regions, N = 5 midguts; 18-h post-heat shock, % of ISCs = 95.06 ± 1.52%, n = 25 regions, N = 5 midguts).

D The T2A histone transgene design: *esg-Gal4 > UASp-H3-eGFP-T2A-H3-mCherry-PolyA*. This design allows for both H3-eGFP and H3-mCherry to be expressed at the same time and in equal molar ratios, utilizing the self-cleaving T2A peptide in between *H3-eGFP* and *H3-mCherry* sequences (see Materials and Methods).

E A western blot targeting eGFP in protein extracted from intestines expressing the *UASp-H3-eGFP-T2A-H3-mCherry-PolyA* transgene. One band can be observed at ~40 kDa, corresponding to H3-eGFP. The uncleaved protein of H3-eGFP-H3-mCherry would be observed at ~80 kDa, indicating the self-cleaving T2A peptide is efficiently cleaving.

F Comparison of Pearson and Spearman correlation coefficients to determine the colocalization of eGFP- and mCherry- or mKO-tagged histones in prophase and prometaphase ISCs in different transgene designs. Spearman correlation coefficients are slightly different when compared to Pearson correlation coefficients. There are no significant differences between Pearson and Spearman correlation coefficients for H4 [avg. Pearson correlation coefficient for H4 = 0.51 ± 0.02 (Fig 2C), avg. Spearman correlation coefficient for H4 = 0.55 ± 0.02, n = 31 ISCs] H2A [avg. Pearson correlation coefficient for H2A = 0.71 ± 0.02 (Fig 2C), avg. Spearman correlation coefficient for H2A = 0.74 ± 0.01, n = 50 ISCs] and H3T3A [avg. Pearson correlation coefficient for H3T3A = 0.49 ± 0.02 (Fig 4B), avg. Spearman correlation coefficient for H3T3A = 0.53 ± 0.02, n = 55 ISCs] analyses. There is a significant difference between the Pearson and Spearman correlation coefficients for H3 [avg. Pearson correlation coefficient for H3 = 0.44 ± 0.02 (Fig 1F), avg. Spearman correlation coefficient for H3 = 0.51 ± 0.02, n = 50 ISCs, **P < 0.01] and T2A-H3 co-expression [avg. Pearson correlation coefficient for T2A-H3 = 0.86 ± 0.01 (Fig 1F), avg. Spearman correlation coefficient for T2A-H3 = 0.79 ± 0.02, n = 19 ISCs, **P < 0.01]; however, the trend of correlation coefficients among different histones and labeling methods is the same when comparing results using these two analytic methods. When comparing the Spearman correlation coefficients of the H3 dataset to T2A-H3, H4, H2A, and H3T3A, the results remain consistent with that of the Pearson's analysis (shown in Figs 1F, 2C, and 4B), where H3 is significantly different from T2A-H3 and H2A (****P < 0.0001), but not significantly different from H4 or H3T3A (n.s.). Additionally, H2A is significantly different from H3T3A (****P < 0.0001), consistent with the Pearson's data shown in Fig 4B. Individual data points and mean values are shown. Error bars represent SEM. ****P < 0.0001, **P < 0.01, NS, not significant; unpaired t-test to compare two individual datasets to each other. Individual data values are shown in Dataset EV1.

Data information: n for individual ISCs. Scale bar in (A) and (B): 50 μm.

© 2023 The Authors

© 2023 The Authors

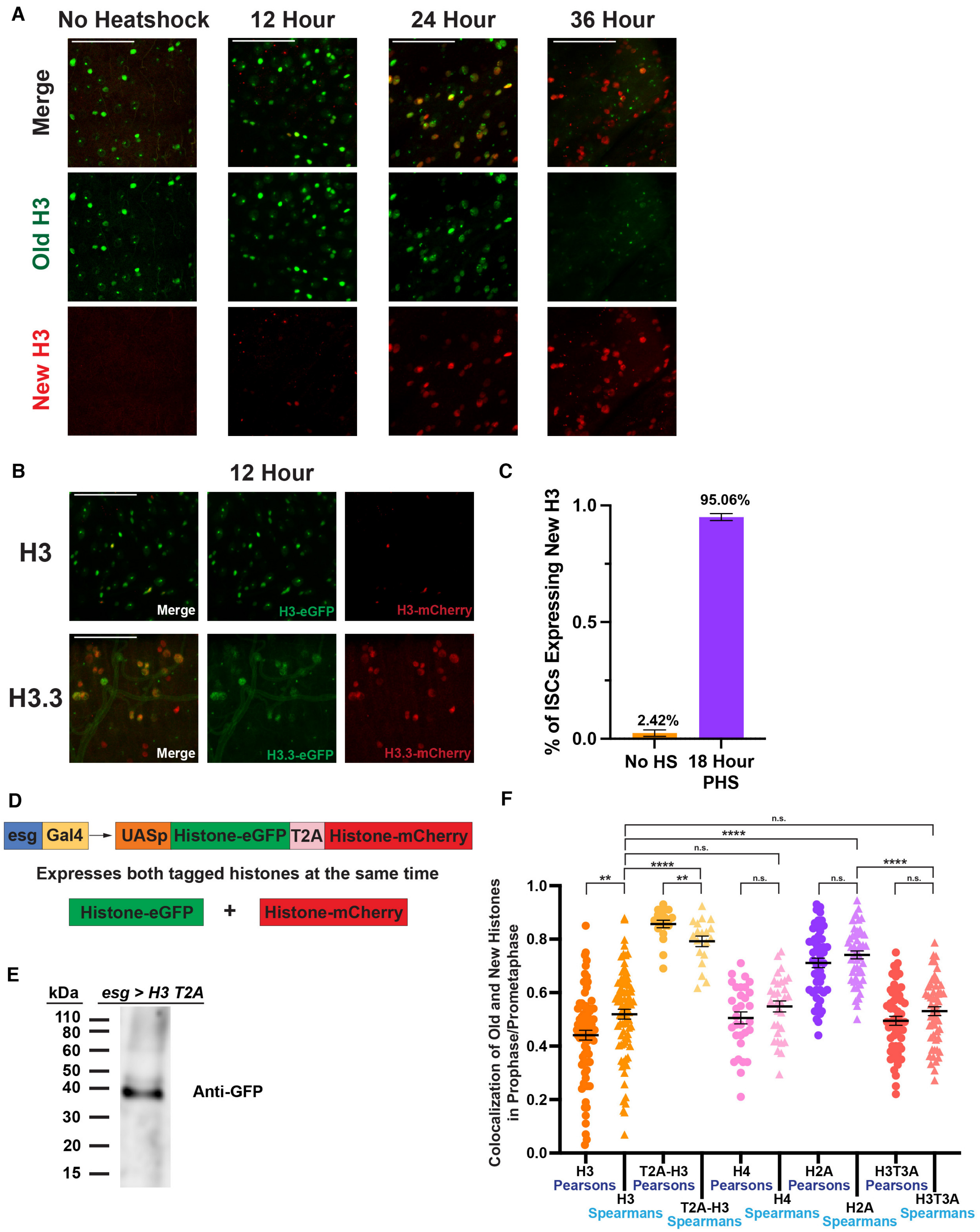

Figure EV1.

© 2023 The Authors

Figure EV2. Related to Fig 3: Old and new H3 expressed using the ISC-specific combination of drivers esg-Gal4, Su(H)-Gal80 shows similar expression patterns when compared to using the esg-Gal4 driver. Co-expressed eGFP- and mCherry-tagged H3 show symmetric inheritance in both Delta-asymmetric and Delta-symmetric pairs.

A A representative image showing expression of the dual-color histone H3 transgene using the *esg-Gal4* driver with heat-shock treatment and recovery, using *esg-Gal4 > UASp-FRT-H3-eGFP-FRT-H3-mCherry*. Both eGFP (Green)- and mCherry (Red)-tagged histones can be visualized. *Dl-nLacZ* expression (Gray) is used for ISC marker. Cells with positive EdU signal introduced by a pulse labeling (Magenta) can be detected and are specified by the magenta circle.

B A representative image showing expression of the dual-color histone H3 transgene using the combination of *esg-Gal4*, *Su(H)-Gal80 > UASp-FRT-H3-eGFP-FRT-H3-mCherry* with heat-shock treatment and recovery, which shows ISC-specific pattern. Both eGFP (Green)- and mCherry (Red)-tagged histones can be visualized. *Dl-nLacZ* expression (Gray) is used for ISC marker. Cells with positive EdU signal (Magenta) introduced by a pulse labeling can be detected and are specified by the magenta circle.

C Co-expression of eGFP- and mCherry-tagged H3 using the transgene shown in Fig EV1 (*esg-Gal4 > UASp-H3-eGFP-T2A-H3-mCherry-PolyA*) in a Delta-asymmetric pair of cells: eGFP- and mCherry-tagged H3 distribution in a postmitotic pair of cells with asymmetric Dl-nLacZ labeling, showing that H3-eGFP and H3-mCherry are equally inherited in the Delta-high cell and the Delta-low cell.

D Co-expression of eGFP- and mCherry-tagged H3 in a Delta-symmetric pair of cells: eGFP- and mCherry-tagged H3 distribution in a postmitotic pair of cells with symmetric Dl-nLacZ labeling, showing that H3-eGFP and H3-mCherry are equally inherited in the two cells.

E Quantification of H3-eGFP and H3-mCherry distribution in Delta-asymmetric pair of cells in the co-expressed H3 line (avg. $\log_2$ ratio for eGFP-H3 = 0.03 ± 0.08, avg. $\log_2$ ratio for mCherry-H3 = 0.01 ± 0.06, n = 15 pairs, N = 2 intestines), compared with old H3-eGFP and new H3-mCherry quantification from the *esg-Gal4* driven experiment in Fig 3C. Individual data points and mean values are shown. Error bars represent the standard error of the mean (SEM). ****$P < 0.0001$. An unpaired two-sample t-test to compare two individual datasets to each other. Individual data values are shown in Dataset EV2 and Table EV5.

F Quantification of H3-eGFP and H3-mCherry distribution in Delta-symmetric pair of cells in the co-expressed H3 line (avg. $\log_2$ ratio for GFP-H3 = 0.07 ± 0.08, avg. $\log_2$ ratio for mCherry-H3 = 0.00 ± 0.07, n = 11 pairs, N = 2 intestines), compared with old H3-eGFP and new H3-mCherry quantification using the *esg-Gal4* driver shown in Fig 3C. Individual data points and mean values are shown. Error bars represent the standard error of the mean (SEM). N.S.: not significant. Unpaired t-test to compare two individual datasets to each other. Individual data values are shown in Dataset EV2 and Table EV8. Between the two cells, cell 1 has the higher Dl-nLacZ level compared with cell 2 (Materials and Methods).

Data information: Scale bar in (A) and (B): 50 µm. Scale bar in (C) and (D): 5 µm; asterisk, ISC side.

© 2023 The Authors

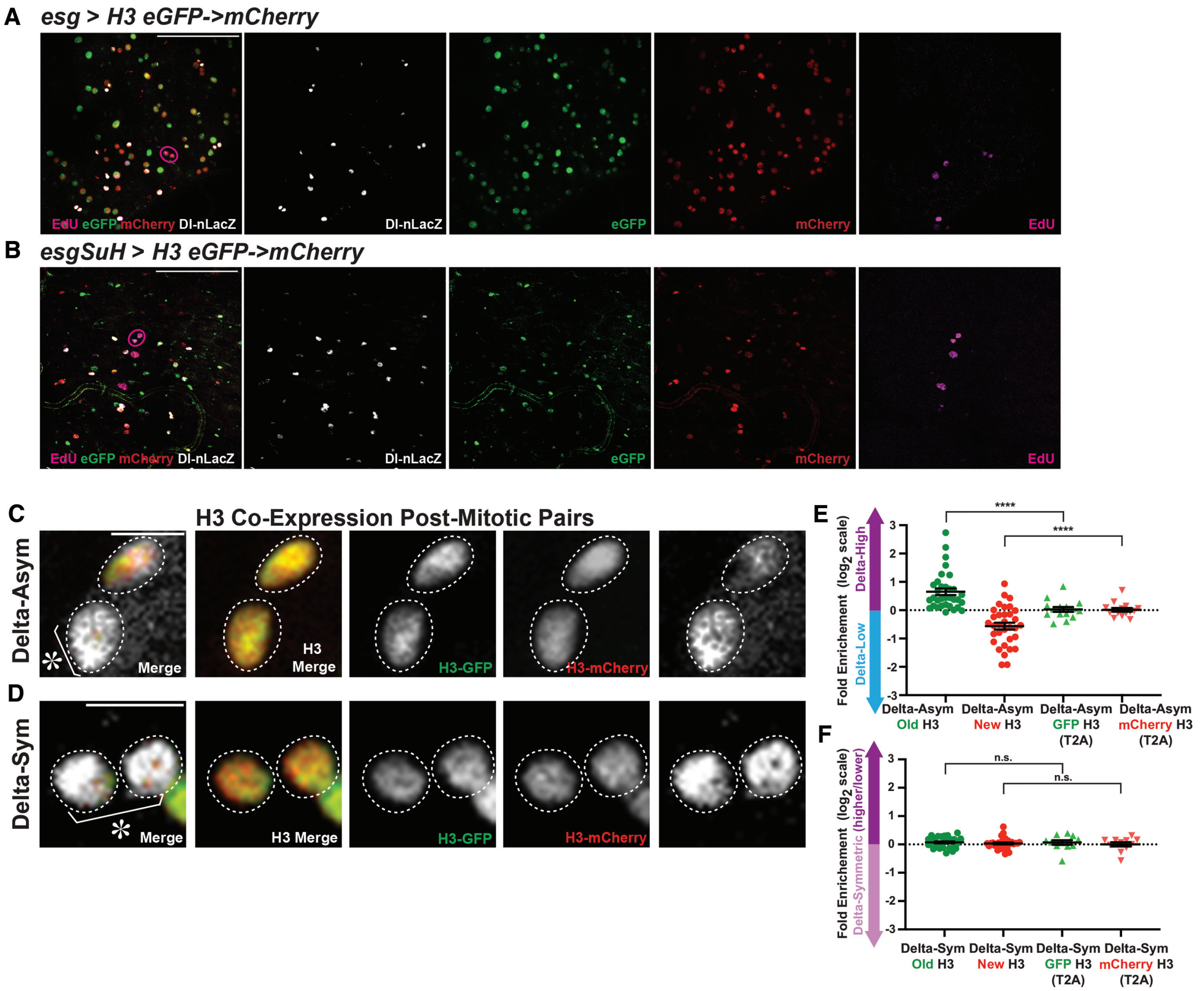

Figure EV2.

 © 2023 The Authors

Figure EV3. Related to Figs 3, 5, and 6: Old and new histone H3 displays asymmetric inheritance pattern in pairs of cells when asymmetric versus symmetric Delta expression is identified using immunostaining with Delta antibody. Additionally, the internal driver marker for ISCs, YFP, shows distinct overlap with Dl-nLacZ, marking ISCs, and separable signals with Su(H)-LacZ, marking EBs.

A Old and new H3 distribution in a postmitotic pair of cells with punctate Delta antibody staining, showing that H3-eGFP (old) is asymmetrically inherited by the Delta-high cell, while H3-mCherry (new) is enriched in the Delta-low cell, using *esg-Gal4* > *UASp-FRT-H3-eGFP-FRT-H3-mCherry*.

B Old and new H3 distribution in a postmitotic pair of cells with punctate Delta antibody staining in both cells, showing that both H3-eGFP (old) and H3-mCherry (new) are symmetrically distributed between the two cells with symmetric Delta expression pattern.

C Comparisons of the quantification of old H3 in postmitotic pairs of cells using the Delta-nLacZ reporter line (data from Fig 3C), and the Delta antibody to identify pairs of cells with Delta-asymmetric or Delta-symmetric expression patterns. Quantification of H3-eGFP (old) in Delta-asymmetric pairs identified by the Delta antibody (avg. $\log_2$ ratio for old H3 = 0.71 ± 0.10, n = 41 pairs) is similar to that of Delta-asymmetric pairs identified by the Delta-nLacZ reporter (avg. $\log_2$ ratio for old H3 = 0.65 ± 0.12, n = 33 pairs, Fig 3C). Similarly, quantification of H3-eGFP (old) in Delta-symmetric pairs was similar between pairs identified by the Delta antibody (avg. $\log_2$ ratio for old H3 = −0.003 ± 0.09, n = 10 pairs) and pairs identified by the Delta-nLacZ reporter (avg. $\log_2$ ratio for old H3 = 0.07 ± 0.03, n = 30 pairs, Fig 3C).

D Old and new H2A distribution in a postmitotic pair of cells with asymmetric punctate Delta antibody staining, showing that H2A-eGFP (old) and H2A-mCherry (new) are symmetrically inherited by the two cells, using *esg-Gal4* > *UASp-FRT-H2A-eGFP-FRT-H2A-mCherry*.

E Old and new H2A distribution in a postmitotic pair of cells with symmetric punctate Delta antibody staining, showing that both H2A-eGFP (old) and H2A-mCherry (new) are symmetrically distributed between the two cells.

F Comparisons of the quantification of old H2A in postmitotic pairs of cells using the Delta-nLacZ reporter line (data from Fig 3K), or the Delta antibody. Quantification of H2A-eGFP (old) in Delta-asymmetric pairs identified by the Delta antibody (avg. $\log_2$ ratio for old H2A = 0.002 ± 0.08, n = 32 pairs) is similar to that of Delta-asymmetric pairs identified by the Delta-nLacZ reporter (avg. $\log_2$ ratio for old H2A = −0.03 ± 0.07, n = 30 pairs, Fig 3K). Similarly, quantification of H2A-eGFP (old) in Delta-symmetric pairs was similar between pairs identified by the Delta antibody (avg. $\log_2$ ratio for old H2A = 0.03 ± 0.06, n = 6 pairs) and pairs identified by the Delta-nLacZ reporter (avg. $\log_2$ ratio for old H2A = 0.08 ± 0.03, n = 30 pairs, Fig 3K).

G Comparison of YFP and Dl-nLacZ expression. YFP was driven by the ISC-specific driver, *esg-Gal4*, *Su(H)-Gal80*, *tub-Gal80^{ts}* at the Gal80^{ts} restrictive temperature (29°C). Significant overlap of YFP and Dl-nLacZ is observed, with both signals marking ISCs.

H Comparison of YFP and Su(H)-LacZ expression. YFP was driven by the ISC-specific driver, *esg-Gal4*, *Su(H)-Gal80*, *tub-Gal80^{ts}* at the Gal80^{ts} restrictive temperature (29°C). Separable signals of YFP, marking ISCs, and Su(H)-LacZ, marking EBs, are observed.

Data information: n for the number of postmitotic pairs. For (C) and (F), individual data points and mean values are shown. Error bars represent SEM. ****P < 0.0001, *P < 0.05; single-sample t-test (for normally distributed data) for comparing one dataset to a hypothesized mean of 0 ($\log_2$ value = 0 representing a 1:1 ratio), or Wilcoxon signed-rank test (for skewed data) for comparing one dataset to a hypothesized median of 0. Unpaired t-test to compare two individual datasets to each other. NS, not significant. Individual data values are shown in Dataset EV4. Scale bar in (A), (B), (D) and (E): 5 μm; Scale bar in (G) and (H): 50 μm; asterisk, ISC side.

© 2023 The Authors

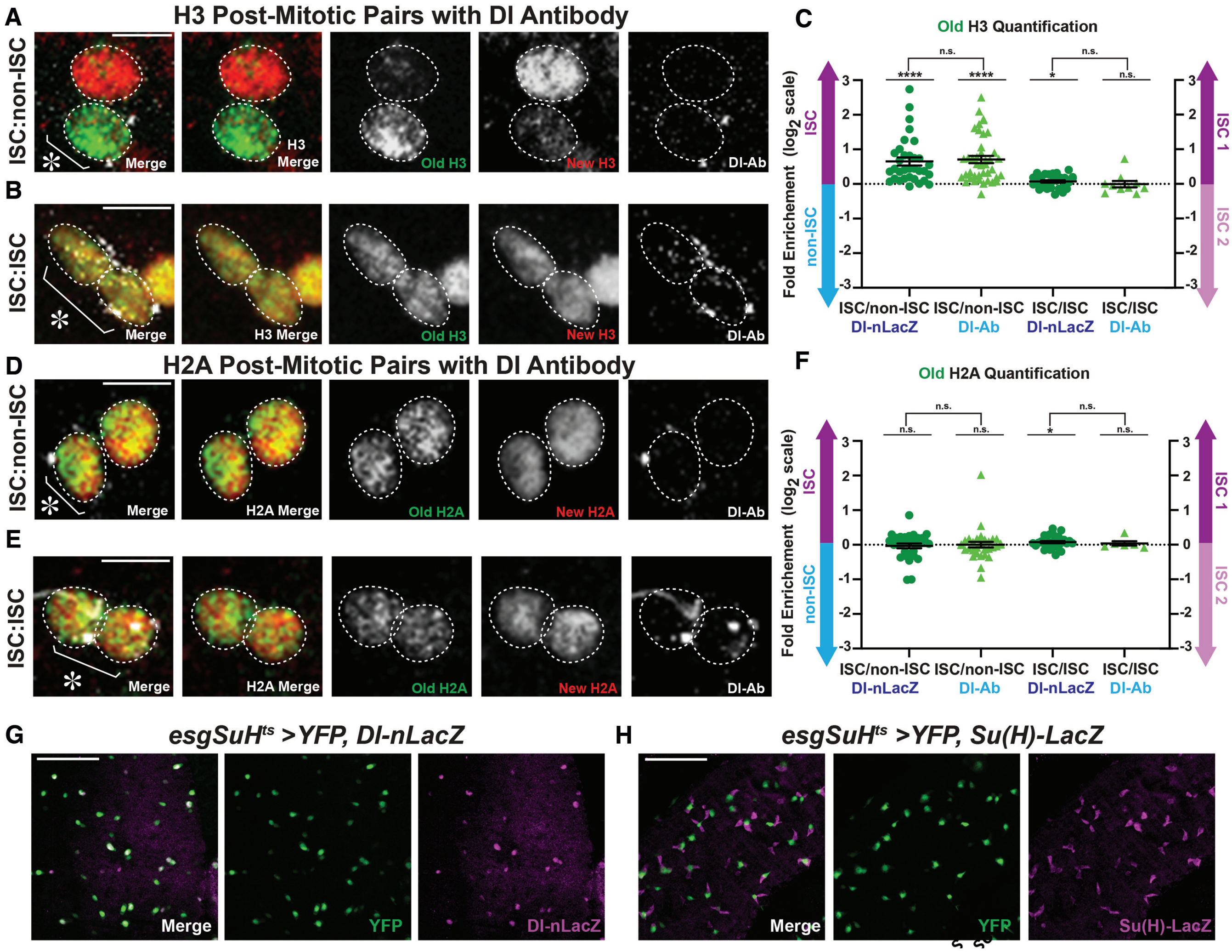

Figure EV3.

Figure EV4. Related to Fig 6. Single-cell RNA-seq using H3-expressing and H3T3A-expressing intestinal samples show compromised ISC differentiation in the H3T3A-expressing samples.

A, B Clusters were identified from a list of marker genes (Hung *et al*, 2020). The H3 and H3T3A samples are very similar in the average expression levels of these different clusters. Only H3T3A (after quality control) contained a cluster with no expression of any marker which uniquely identified the other clusters (unknown).

C We fitted a regression model to predict Delta levels in pseudotime (*x*-axis). Our outlier removal (outliers removed in Fig 6I) did not affect the significance level, but did increase the slope of the wild-type regression line by roughly twofold, which is a desirable quality.

D The regression model for klu levels is also similar to its counterpart after outlier removal (Fig 6J). Outliers did not change the direction of the slope or the significance level, but had a negative effect on the wild-type line, which has a small slope here.

E–H The exclusion of outliers [the Unknown class of the scType classifier (Ianevski *et al*, 2022)] is detailed. scType may be affected by differences in separability between Dl^+ and klu^+ populations in H3T3A. The outliers do not follow the same pattern in the H3 and H3T3A samples, but we chose an off-the-shelf solution for removal of outliers based on marker genes, to avoid biasing the result. We removed outliers shown in (E and F); compare (C) with Fig 6I. We removed outliers shown in (G and H); compare (D) with Fig 6J.

 © 2023 The Authors

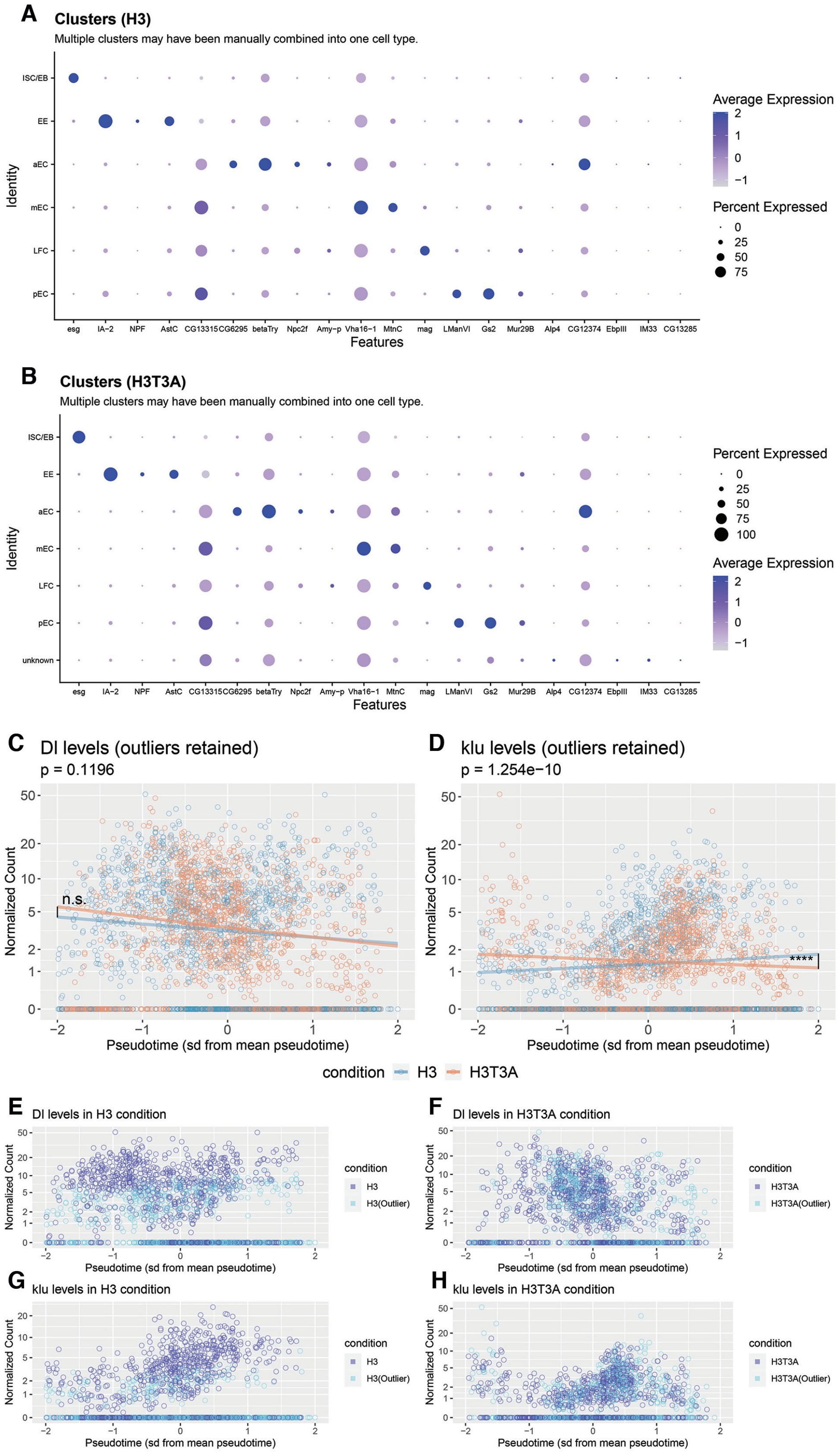
A
Clusters (H3)
Multiple clusters may have been manually combined into one cell type.
Identity
ISC/EB
EE
aEC
mEC
LFC
pEC
esg IA−2 NPF AstC CG13315 CG6295 betaTry Npc2f Amy−p Vha16−1 MtnC mag LManVI Gs2 Mur29B Alp4 CG12374 EbpIII IM33 CG13285
Features
Average Expression
Percent Expressed
B
Clusters (H3T3A)
Multiple clusters may have been manually combined into one cell type.
unknown
C
Dl levels (outliers retained)
p = 0.1196
n.s.
Normalized Count
Pseudotime (sd from mean pseudotime)
D
klu levels (outliers retained)
p = 1.254e−10
****
condition
H3
H3T3A
E
Dl levels in H3 condition
H3(Outlier)
F
Dl levels in H3T3A condition
H3T3A(Outlier)
G
klu levels in H3 condition
H
klu levels in H3T3A condition

Figure EV4.

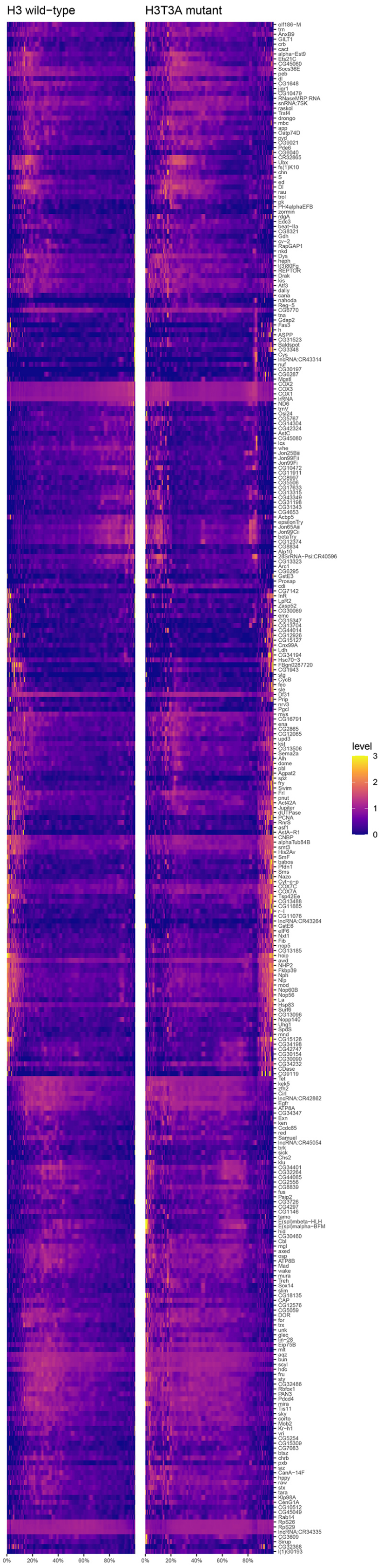

Figure EV5. Related to Fig 6. Single-cell RNA-seq data showing representative individual gene expression profiles in H3-expressing and H3T3A-expressing intestinal samples.

H3 and H3T3A pseudotime is shown as gene levels varying from left to right. Genes are selected for significance according to the tradeSeq nonlinear differential expression library (Van den Berge *et al*, 2020). Note that klu levels are low where Dl levels are high, and vice versa.

© 2023 The Authors
