# Supplementary material for: Old and newly synthesized histones are asymmetrically distributed in Drosophila intestinal stem cell divisions
Source: EMBO Rep. 2023 May 31;24(7):e56404. doi: 10.15252/embr.202256404 (PMC10328082; doi:10.15252/embr.202256404)
Supplement: Supplementary file 15 [file EMBR-24-e56404-s015.pdf]

EMBO reports

Old and newly synthesized histones are asymmetrically distributed in Drosophila intestinal stem cell divisions

Emily Zion, Daniel Ringwalt, Kristina Rinaldi, Elizabeth Kahney, Yingying Li, and Xin Chen
DOI: 10.15252/embr.202256404

Corresponding author(s): Xin Chen (xchen32@jhu.edu)

Review Timeline:

Submission Date:	31st Oct 22
Editorial Decision:	8th Dec 22
Revision Received:	30th Mar 23
Editorial Decision:	26th Apr 23
Revision Received:	30th Apr 23
Accepted:	16th May 23

Editor: Esther Schnapp

Transaction Report:

(Note: With the exception of the correction of typographical or spelling errors that could be a source of ambiguity, letters and reports are not edited. Depending on transfer agreements, referee reports obtained elsewhere may or may not be included in this compilation. Referee reports are anonymous unless the Referee chooses to sign their reports.)

Dear Dr. Chen,

Thank you for your patience while your manuscript was peer-reviewed at EMBO reports. We have now received the full set of referee reports that is pasted below.

As you will see, the referees acknowledge that the findings are potentially interesting. However, they also have several suggestions for how the study should be improved and strengthened. I am sending you all comments now so that you can have a look and let me know whether you can address all and/or which ones you prefer not to or cannot address experimentally. Please let me know what you think.

Given the overall supportive reports, I would like to invite you to revise your manuscript with the understanding that the referee concerns must be fully addressed and their suggestions taken on board. Please address all referee concerns in a complete point-by-point response. Acceptance of the manuscript will depend on a positive outcome of a second round of review. It is EMBO reports policy to allow a single round of major revision only and acceptance or rejection of the manuscript will therefore depend on the completeness of your responses included in the next, final version of the manuscript.

We realize that it is difficult to revise to a specific deadline. In the interest of protecting the conceptual advance provided by the work, we recommend a revision within 3 months (10th Mar 2023). Please discuss the revision progress ahead of this time with the editor if you require more time to complete the revisions.

IMPORTANT NOTE: we perform an initial quality control of all revised manuscripts before re-review. Your manuscript will FAIL this control and the handling will be DELAYED if the following APPLIES:
1) A data availability section providing access to data deposited in public databases is missing. If you have not deposited any data, please add a sentence to the data availability section that explains that.
2) Your manuscript contains statistics and error bars based on n=2. Please use scatter blots in these cases. No statistics should be calculated if n=2.

When submitting your revised manuscript, please carefully review the instructions that follow below. Failure to include requested items will delay the evaluation of your revision.

1) a .docx formatted version of the manuscript text (including legends for main figures, EV figures and tables). Please make sure that the changes are highlighted to be clearly visible.

2) individual production quality figure files as .eps, .tif, .jpg (one file per figure). See https://wol-prod-cdn.literatumonline.com/pb-assets/embo-site/EMBOPress_Figure_Guidelines_061115-1561436025777.pdf for more info on how to prepare your figures.

3) We replaced Supplementary Information with Expanded View (EV) Figures and Tables that are collapsible/expandable online. A maximum of 5 EV Figures can be typeset. EV Figures should be cited as 'Figure EV1, Figure EV2" etc... in the text and their respective legends should be included in the main text after the legends of regular figures.

- For the figures that you do NOT wish to display as Expanded View figures, they should be bundled together with their legends in a single PDF file called *Appendix*, which should start with a short Table of Content. Appendix figures should be referred to in the main text as: "Appendix Figure S1, Appendix Figure S2" etc. See detailed instructions regarding expanded view here:
<https://www.embopress.org/page/journal/14693178/authorguide#expandedview>

- Additional Tables/Datasets should be labeled and referred to as Table EV1, Dataset EV1, etc. Legends have to be provided in a separate tab in case of .xls files. Alternatively, the legend can be supplied as a separate text file (README) and zipped together with the Table/Dataset file.

4) a .docx formatted letter INCLUDING the reviewers' reports and your detailed point-by-point responses to their comments. As part of the EMBO Press transparent editorial process, the point-by-point response is part of the Review Process File (RPF), which will be published alongside your paper.

5) a complete author checklist, which you can download from our author guidelines
<https://www.embopress.org/page/journal/14693178/authorguide>. Please insert information in the checklist that is also reflected in the manuscript. The completed author checklist will also be part of the RPF.

6) Please note that all corresponding authors are required to supply an ORCID ID for their name upon submission of a revised manuscript (<https://orcid.org/>). Please find instructions on how to link your ORCID ID to your account in our manuscript tracking system in our Author guidelines
<https://www.embopress.org/page/journal/14693178/authorguide#authorshipguidelines>

7) Before submitting your revision, primary datasets produced in this study need to be deposited in an appropriate public database (see https://www.embopress.org/page/journal/14693178/authorguide#datadeposition). Please remember to provide a reviewer password if the datasets are not yet public. The accession numbers and database should be listed in a formal "Data Availability" section placed after Materials & Method (see also https://www.embopress.org/page/journal/14693178/authorguide#datadeposition). Please note that the Data Availability Section is restricted to new primary data that are part of this study. * Note - All links should resolve to a page where the data can be accessed. *
If your study has not produced novel datasets, please mention this fact in the Data Availability Section.

8) At EMBO Press we ask authors to provide source data for the main manuscript figures. Our source data coordinator will contact you to discuss which figure panels we would need source data for and will also provide you with helpful tips on how to upload and organize the files.

9) Our journal also encourages inclusion of *data citations in the reference list* to directly cite datasets that were re-used and obtained from public databases. Data citations in the article text are distinct from normal bibliographical citations and should directly link to the database records from which the data can be accessed. In the main text, data citations are formatted as follows: "Data ref: Smith et al, 2001" or "Data ref: NCBI Sequence Read Archive PRJNA342805, 2017". In the Reference list, data citations must be labeled with "[DATASET]". A data reference must provide the database name, accession number/identifiers and a resolvable link to the landing page from which the data can be accessed at the end of the reference. Further instructions are available at https://www.embopress.org/page/journal/14693178/authorguide#referencesformat

10) Regarding data quantification (see Figure Legends: https://www.embopress.org/page/journal/14693178/authorguide#figureformat)

The following points must be specified in each figure legend:

- the name of the statistical test used to generate error bars and P values,

- the number (n) of independent experiments (please specify technical or biological replicates) underlying each data point,

- the nature of the bars and error bars (s.d., s.e.m.),

- If the data are obtained from n Program fragment delivered error ``Can't locate object method "less" via package "than" (perhaps you forgot to load "than"?) at //ejpvfs23/sites23b/embor_www/letters/embor_decision_revise_and_review.txt line 56.' 2, use scatter blots showing the individual data points.

Discussion of statistical methodology can be reported in the materials and methods section, but figure legends should contain a basic description of n, P and the test applied.

- Please also include scale bars in all microscopy images.

11) The journal requires a statement specifying whether or not authors have competing interests (defined as all potential or actual interests that could be perceived to influence the presentation or interpretation of an article). In case of competing interests, this must be specified in your disclosure statement. Further information: https://www.embopress.org/competing-interests

We would also welcome the submission of cover suggestions, or motifs to be used by our Graphics Illustrator in designing a cover.

As part of the EMBO publication's Transparent Editorial Process, EMBO reports publishes online a Review Process File (RPF) to accompany accepted manuscripts. This File will be published in conjunction with your paper and will include the referee reports, your point-by-point response and all pertinent correspondence relating to the manuscript.

You are able to opt out of this by letting the editorial office know (emboreports@embo.org). If you do opt out, the Review Process File link will point to the following statement: "No Review Process File is available with this article, as the authors have chosen not to make the review process public in this case."

I look forward to seeing a revised form of your manuscript when it is ready. Please use this link to submit your revision: https://embor.msubmit.net/cgi-bin/main.plex

Best regards,
Esther

Esther Schnapp, PhD

Senior Editor
EMBO reports

Referee #1:

In this manuscript the authors investigate how histones H3, H4 and H2A are allocated throughout intestinal stem cell (ISC) division in Drosophila melanogaster. They use an experimental setup in which already existing (old) histones are labeled by eGFP while the new are labeled with mCherry. Upon S phase, old and new histone are reincorporated into DNA and the distribution followed upon segregation in mitosis and in the resulting daughter cells. They observe that histones H3 and H4 exhibit a distinguishable, separate distribution of old vs new H3 and H4 visible already in prometaphase and clearly distinguishable in telophase. On the contrary, histone H2A shows no overlap of the old and new histones. Next, they suggest histones H3 and H4 exhibit an asymmetric segregation and distribution of the old vs new histones in the post-mitotic daughter cells, with differences noted between pairs of cells that may have resulted from asymmetric cell fate acquisition, in which H3 and H4 show asymmetry, versus symmetric cell fate acquisition, in which H3 and H4 are more symmetric. The cause/consequence relationship between the observed histone asymmetry and fate acquisition is not clear. To shed light into the biological significance of their findings, they use a mutated form of histone H3 (H3T3A), previously shown to cause symmetric segregation in germline stem cells. They find that overexpression of this H3T3A is less asymmetrically distributed to the 2 daughter cells when compared to H3, though not as symmetric as previously seen in the germline. They observe a mild increase in stem cell proliferation, which correlates with additional cells expressing stem cell-driven GFP and bGal. Their findings would imply that asymmetric cell fate decision may be made in the previous cell cycle, which is something that I think is not clear in the field and of interest.

Overall, the experiments are well done and properly quantified. Clearly this is a very large amount of work. Nevertheless I have several questions and important concerns about the data interpretation.

1. While the data obtained in prometaphase, anaphase and telophase cells are clear (figs 1, 2), experiments done on "pairs of post-mitotic cells" are potentially problematic to me. In a majority of their experiments (figs 3, S3, 4E-F), they are using a Gal4 driver that is expressed in both ISCs and EBs. ISCs are actually quite quiescent in the tissue, which only a very small frequency undergoing division at any time (see Zielke, 2014). Less than 5% are undergoing S phase at any given time and only about 10 mitotic cells are present per gut. Therefore, during their experiment when they induce the expression of the new histone constructs for 18h, only very few pairs of cells would be expected to have undergone S phase. It is not clear to me how many pairs of cells they are quantifying per gut? Can they give an approximate number? I do not understand how they can be sure that the pairs of cells that they assess in these figures underwent S phase, as opposed to just being neighboring cells that both flip their Histone constructs?
One way to test this directly would be to feed EdU during the 18 hours after heatshock or start slightly before the heat shock. Pairs of cells that underwent S phase will have EdU and this should correlate to the percent of pairs per gut that they analysed. They could use BrDU post-dissection if they need to exclude those currently undergoing S phase as it seems they do in the methods.
A related point: How do they know that they new Histones are incorporated into chromatin as opposed to just being nuclear?

I fully understand that they used the esgGal4 Su(H)-GAL80 construct in some experiments, supporting the idea that these pairs originate from a dividing ISC, but we are not given the numbers of cells that they quantified in both cases (esgGal4 vs esgGal4 Su(H)Gal80) per gut. These should be similar if the in the esgGal4 experiment they are indeed analysing cells that recently underwent S phase and cell division.

2. An important control that I do not see, but which is critical for all of their interpretation, is to assess the level of expression of GFP and RFP in the same flies they are assaying (hs-FLP esgGal4 UAS-Histone construct) but without a heat shock. The hs-FLP as well as the UAS- cassette might have leaky expression, which would impact their ability to analyze pairs of cells as recently resulting from cell division.

3. I question the interpretation of the pairs of Dl-Bgal expressing cells as arising from a "symmetric division": as Bgal is very stable in this tissue, I would imagine that ALL cells that recently divided should give rise to a pair of Bgal+ cells. They should test this by doing an Edu feeding during 18h and then assessing Dl-LacZ in the cells. If their interpretation is correct that Bgal can turn over and distinguish symmetric versus asymmetric pairs of cells during this 18h period, then they should see about 80% of EdU pairs with 1 Dl stong and 1 weak.

4. The authors mention that ISCs are the only dividing cell type in the gut citing older papers. More recent studies suggest that there is an EE precursor cell, which divides once to produce 2 EE cells (see Chen...Xi, NCB, 2018). The authors need to consider this and mention it in their discussion as it could impact their conclusions.

5. In Figure 2D- why is new histone asymmetry not plotted? Shouldn't this mimic what is see in "post-mitotic pairs" in Figure 3?

They mention in the figure legend that they do not do this for a reason mentioned in their 2015 paper, but it should really be restated here. I looked and could not find it easily the information. If there is some caveat in scoring new histone, wouldn't this also apply to their analysis of pairs of cells?

6. In Figure 4I- The proliferation status is very much linked to physiological status of the fly, is often variable and therefore, N=4 midguts is not enough. They should quantify PH3 in a larger number of guts and plot the individual data points with the mean or a box plot. This should be examined in separate experiments with a higher number of guts (N>15).

7. I feel that the model figure 6C is an over-interpretation. While there appears to be a small increase in the number of dividing cells, subsequently resulting in more esgGFP+ cells, I am not convinced that this is a "tumor" of any kind. They do not present any evidence of a differentiation defect or large accumulation of stem cells. I would tone down this model.

Referee #2:

This work provides evidence that biased incorporation of new vs old histone H3/H4 occurs along the entire length of each sister chromatid in fly intestinal stem cells. The resultant two sister chromatids, one incorporated with more old H3/H4 and the other incorporated with more newer H3/H4, are segregated during subsequent cell division. When ISCs divide asymmetrically, the sister chromatid with old H3/H4 stays in a future ISC and the other sister chromatid with new H3/H4 is inherited by a differentiating daughter cell. When ISCs divide symmetrically, these two sisters still show biased incorporation of new vs old H3/H4 but they segregate randomly. Moreover, when this segregation pattern was compromised by expressing non-phosphorylatable H3 (H3T3A), ISC-like cells overproliferate.

Along with a series of works published from the same group, this work provides important information regarding the intrinsic regulation of cell-fate determination during asymmetric stem cell division, such that two sister chromatids made in stem cells are already different right after they are synthesized, even before the cell division. The authors used fly intestinal stem cells which can divide either symmetrically or asymmetrically and demonstrated that the H3/H4 inheritance pattern is correlated to ISC division modes (asymmetric or symmetric). This work, together with their older works, suggests that chromosomes (or sister chromatids) distinctly marked by new vs old histones may contain distinct epigenetic information, and is conserved and linked to cell fate determination in different stem cell systems and as such will be of broad interest to cell and developmental biologists.

Major points;
What is the mechanism of asymmetric incorporation of new/old H3? Authors previously suggested that replication forks may be unidirectional in the male germline. Do the authors consider that a similar mechanism is used in ISCs?

Histone H3 expression is normally limited in S-phase. I would imagine esg-Gal4 driven expression is quite different from endogenous H3 dynamics. I suggest considering using endogenous locus.

The method of labeling system is unclear. How did authors choose the 18-hour time point? Authors should provide the data to show 18-hour point is in next round of S-phase after the flipping event occurred. In addition, authors should provide the percentage of cells that are "flipped-out" Does this proportion change if they use a timepoint other than "18 hours" and if so, how do they interpret those results?

Referee #3:

Zion, Emily H et al present meaningful and original work, indicating that histone inheritance patterns regulate cell fates in adult stem lineages in vivo. Since proper differentiation of intestinal stem cells is essential for intestinal homeostasis, the findings are significant and have the potential to impact several fields. In addition, the authors clarified that compromising asymmetric histone inheritance led to an increase in Delta-symmetric cell pairs and tumors. Generally, the experiments are well designed and the data are powerful. I have some comments and concerns about the experiments and interpretation, which the authors may want to consider for improving the manuscript.

1. In Figure 1, the authors utilized "FRT/FLP" system to study histone distribution and inheritance patterns during ISC divisions. Is there a leakage in the system even without heat shock? If there is a leak, it may affect the results. I would hope that the authors check the leakage of "FRT/FLP" system.

2. In all Figures, old histone and new histone signals were shown with white color, which is not good for reading. I would suggest that the old histone signal is showed a green color and the new histone signal is showed a red color.

3. In Figure 1C, Figure S1B, D, E, and Figure SG-H, genotypes are not written in italics. Hopefully, the author will go into more careful about this.

4. In Figure 4 H, I and Figure 5 A-D, the authors used white color to present DAPI signal, which is not good for reading. Whether can replace the white color with blue color to show the DAPI?

5. In Figures 4 and 5, the authors found that disruption of asymmetric histone inheritance patterns resulted in increased Delta-positive ISC-like cells. Is it the disruption of asymmetrical division that leads to the mis-differentiation of ISCs, resulting in ISC accumulation? Compare the number of differentiated cells (ECs or EEs) in wild-type and histone mutants.

Referee #4:

The manuscript 'Histone inheritance patterns in Drosophila intestinal stem cells' by Zion et al. studies the distribution of H3 in intestinal stems cells (ISC) of the Drosophila midgut. In this manuscript the used a ISC-specific Gal4 driver to express their previously used dual labelling system specifically in ISCs. The authors find that there is an unequal distribution of new H3 and old H3 that was already present at the previous cell cycle. They have previously used this system to show an asymmetric distribution in germ line stem cells. The find in both systems that newly made proteins tend to get preferentially inherited in the differentiating cells whereas the previously present protein remains in the stem cell. This is a very neat system that the authors have developed a few years ago and shown that is works well to distinguish histone inheritance. The results in this manuscript are clear and the experiments well executed and in a technically sound manner. The quality of the presented results is very good. However, the manuscript lacks any major new advances in our understanding of stem cell chromatin inheritance as most of their results have been described in similar (but not identical) ways before and as detailed below.

The authors claim that they see the unequal distribution of old and new H3 histones already in prometaphase as this is the time when chromosomes start to align. What wasn't clear from the manuscript is whether the transgenes are incorporated into the ISC during replication like the endogenous proteins or whether they either get incorporated outside of replication or are actually not incorporated at all but only associated with chromatin. The authors must make it clear from which results they can conclude that their tagged versions are also replication dependent deposited onto chromatin and how this then translate into an asymmetric distribution in prometaphase.

The authors mention in their manuscript that the same inheritance pattern has been shown before in a different stem cell linage in the same organisms but they fail to clearly mention that it is a similar inheritance pattern that has been described in the same intestinal stem cells with the histone H3 variant CID using the same dual labelling technique. This is important since they authors claim in their introduction that it isn't known whether asymmetric histone inheritance is a germ line-specific event or also occurs in other stem cells like the ISC ("The question remains, however, of whether this phenomenon is germ cell-specific or if it serves as a more general mechanism."). They continue to ignore that asymmetric inheritance has already been established in vivo in ISC ('We propose that the ISC lineage is a great system to study histone inheritance due to its well-characterized lineage, clearly distinguishable ISC-specific mitosis, and abundant ISCs in vivo.').

What is somewhat difficult to understand is what the differences are in Figure 1, 2 and 3 in terms of their results. All histones and also the Delta staining could easily have been shown in one figures especially because this is mainly recaps from previous papers from the lab in a different stem cell line.

Also in Figure 4, most of their results were in clear accordance with what they have seen in male GSC. What they could have done here to potentially obtain new results that wouldn't be possible in GSC is the infection of Drosophila with gut pathogens that change SCD and ACD. It would be interesting to know if the H3T3 mutant would lead to a more severe phenotype.

I am not sure about their terminology of their results of the histone H3T3A mutant. The inheritance seems fine but there are more symmetric SCD. This is also observed when the Drosophila gut is injured or infected but I would not call it 'mis-inheritance', the inheritance is fine but the gut seems to produce more stem cells just like after infection. I also think that the 'ISC tumor formation' is not necessarily backed up by experiments in this paper. There is a clear overproliferation but overproliferation alone doesn't make a tumor.

Minor Points:

The first paragraph of the introduction is too broad. For instance, why do the authors mention DNA methylation when the model organism of this paper doesn't have DNA methylation? A more to the point introduction would benefit the paper.

Point-to-point responses to reviewers' questions and comments:

Referee #1:

In this manuscript the authors investigate how histones H3, H4 and H2A are allocated throughout intestinal stem cell (ISC) division in Drosophila melanogaster. They use an experimental setup in which already existing (old) histones are labeled by eGFP while the new are labeled with mCherry. Upon S phase, old and new histone are reincorporated into DNA and the distribution followed upon segregation in mitosis and in the resulting daughter cells. They observe that histones H3 and H4 exhibit a distinguishable, separate distribution of old vs new H3 and H4 visible already in prometaphase and clearly distinguishable in telophase. On the contrary, histone H2A shows no overlap of the old and new histones. Next, they suggest histones H3 and H4 exhibit an asymmetric segregation and distribution of the old vs new histones in the post-mitotic daughter cells, with differences noted between pairs of cells that may have resulted from asymmetric cell fate acquisition, in which H3 and H4 show asymmetry, versus symmetric cell fate acquisition, in which H3 and H4 are more symmetric. The cause/consequence relationship between the observed histone asymmetry and fate acquisition is not clear. To shed light into the biological significance of their findings, they use a mutated form of histone H3 (H3T3A), previously shown to cause symmetric segregation in germline stem cells. They find that overexpression of this H3T3A is less asymmetrically distributed to the 2 daughter cells when compared to H3, though not as symmetric as previously seen in the germline. They observe a mild increase in stem cell proliferation, which correlates with additional cells expressing stem cell-driven GFP and bGal. Their findings would imply that asymmetric cell fate decision may be made in the previous cell cycle, which is something that I think is not clear in the field and of interest.

We thank this reviewer for the overall positive comments. Indeed, as this reviewer pointed out: The point that the differential incorporation of old vs. new H3 could be made in the previous cell cycle as long as the cells have the ISC identity, but the segregation pattern may rely on the asymmetric vs. symmetric ISC division modes, could be interesting to the field.

Overall, the experiments are well done and properly quantified. Clearly this is a very large amount of work. Nevertheless I have several questions and important concerns about the data interpretation.

1. While the data obtained in prometaphase, anaphase and telophase cells are clear (figs 1, 2), experiments done on "pairs of post-mitotic cells" are potentially problematic to me. In a majority of their experiments (figs 3, S3, 4E-F), they are using a Gal4 driver that is expressed in both ISCs and EBs. ISCs are actually quite quiescent in the tissue, which only a very small frequency undergoing division at any time (see Zielke, 2014). Less than 5% are undergoing S phase at any given time and only about 10 mitotic cells are present per gut. Therefore, during their experiment when they induce the expression of the new histone constructs for 18h, only very few pairs of cells would be expected to have undergone S phase. It is not clear to me how many pairs of cells they are quantifying per gut? Can they give an approximate number? I do not understand how they can be sure that the pairs of cells that they assess in these figures underwent S phase, as opposed to just being neighboring cells that both flip their Histone constructs?
One way to test this directly would be to feed EdU during the 18 hours after heatshock or start

slightly before the heat shock. Pairs of cells that underwent S phase will have EdU and this should correlate to the percent of pairs per gut that they analysed. They could use BrDU post-dissection if they need to exclude those currently undergoing S phase as it seems they do in the methods.
A related point: How do they know that they new Histones are incorporated into chromatin as opposed to just being nuclear?

I fully understand that they used the esgGal4 Su(H)-GAL80 construct in some experiments, supporting the idea that these pairs originate from a dividing ISC, but we are not given the numbers of cells that they quantified in both cases (esgGal4 vs esgGal4 Su(H)Gal80) per gut. These should be similar if the in the esgGal4 experiment they are indeed analysing cells that recently underwent S phase and cell division.

We provided detailed number of individual pairs in these experiments using the *esg-Gal4* driver vs. *esg-Gal4 Su(H)Gal80* combination.

Indeed, as this reviewer pointed out, using the *esg-Gal4 Su(H)Gal80* eliminates the concern that *esg-Gal4* by itself may not be ISC-specific enough. When using the *esg-Gal4 Su(H)Gal80* combination, the two neighboring ISC-EB displaying transgenic histone expression should be derived from a recent ISC division. This point can be further demonstrated by the image with *esg-Gal4 Su(H)Gal80* to drive YFP expression to label ISCs and the Su(H)lacZ reporter to label EBs, where the YFP- and LacZ-positive cells are always identified as pairs juxtaposed to each other (see revised Figure EV3H). In contrast, *esg-Gal4 Su(H)Gal80*-driving YFP and Delta-LacZ always show overlapping YFP- and LacZ-positive cells (see revised Figure EV3G). In summary, these results demonstrate these reporters can reliably label each of the cell types in this stem cell lineage.

In addition, we included images showing that new H3 incorporation depends on S phase. Here, a short-term recovery post heat shock does not lead to robust new H3 incorporation, in contrast to the robust new H3.3 incorporation, which is replication-independent (see revised Figure EV1B). Therefore, with this experimental design new H3 incorporation serves with the similar purpose just like the EdU feeding assay as this reviewer suggested.

Moreover, in these sample after fixation and multiple washing steps, the free new histones are mainly washed off. Even if there are some residual free new histones, it would affect both cells. However, in our images, new histones co-localize with other markers such as DNA dye (DAPI) and mitotic chromatin marker (H3S10ph), indicating it is not just in the nucleoplasm.
With all these criteria discussed above, the only caveat that may still exist is the two ISCs next to each other that could flip out the old histone-coding sequence simultaneously and have a comparable old vs. new histone distribution pattern. However, if this is the case, we should also encounter a situation the two ISCs either do not flip out the transgene at the same time or do not undergo cell cycle progression synchronously, for which one of these two ISCs should contain significantly more old or more new histones. However, we have not detected such patterns, suggesting that the two adjacent ISCs carrying both old and new histones are derived from one symmetric ISC division.

2. An important control that I do not see, but which is critical for all of their interpretation, is to assess the level of expression of GFP and RFP in the same flies they are assaying (hs-FLP esgGal4 UAS-Histone construct) but without a heat shock. The hs-FLP as well as the UAS-cassette might have leaky expression, which would impact their ability to analyze pairs of cells as recently resulting from cell division.

We have done this experiment and provided the no heat shock control, which show minimal pre-flipped cells (see revised Figure EV1A).

3. I question the interpretation of the pairs of Dl-Bgal expressing cells as arising from a "symmetric division": as Bgal is very stable in this tissue, I would imagine that ALL cells that recently divided should give rise to a pair of Bgal+ cells. They should test this by doing an Edu feeding during 18h and then assessing Dl-LacZ in the cells. If their interpretation is correct that Bgal can turn over and distinguish symmetric versus asymmetric pairs of cells during this 18h period, then they should see about 80% of EdU pairs with 1 Dl stong and 1 weak.

First, in Figure EV3 we have provided results using antibodies against Delta protein instead of using the Dl-LacZ reporter, which showed similar results. Second, in Figure 4G we did perform the Dl-LacZ-asymmetric and Dl-LacZ-symmetric analysis, which showed almost exact 80/20 ratio being mentioned here (79.6% asymmetric vs. 20.4% symmetric pairs). Third, in the revised Figure EV3G we showed that *esg-Gal4 Su(H)Gal80*-driving YFP and Delta-LacZ almost always display overlapping YFP and LacZ signals. Finally, in the revised Figure EV3H we showed that YFP expression driven by the *esg-Gal4 Su(H)Gal80* labels ISCs and the LacZ expression using the Su(H)lacZ labels EBs. Here, the YFP- and LacZ-positive cells are almost always identified as pairs juxtaposed to each other with one YFP-positive cell and one LacZ-positive cell. Taken these results together, both YFP and LacZ reporters indeed show cell type specificity reliably, without too much confusion caused by protein perdurance, a concern that this reviewer raised.

4. The authors mention that ISCs are the only dividing cell type in the gut citing older papers. More recent studies suggest that there is an EE precursor cell, which divides once to produce 2 EE cells (see Chen...Xi, NCB, 2018). The authors need to consider this and mention it in their discussion as it could impact their conclusions.

We revised this statement and cited the more recent work. Although with the current design using the *esg-Gal4* driver or the *esg-Gal4 Su(H)Gal80* combination with the mitotic H3S10ph marker, we should not confuse the ISC cell division with the EE precursor cell division.

5. In Figure 2D- why is new histone asymmetry not plotted? Shouldn't this mimic what is see in "post-mitotic pairs" in Figure 3? They mention in the figure legend that they do not do this for a reason mentioned in their 2015 paper, but it should really be restated here. I looked and could not find it easily the information. If there is some caveat in scoring new histone, wouldn't this also apply to their analysis of pairs of cells?

We apologize that the rationale of this quantification was not clear in the initial submission. Here is what we stated in the previous 2015 paper where GFP labels old and mKO labels new histone

in the *Drosophila* male germline stem cells (GSCs, here GB refers to the differentiating daughter cell derived from asymmetric germline stem cell division): “We reasoned that GFP ratio reflects the establishment of asymmetric histone distribution on sister chromatids more reliably than mKO ratio for two reasons. First, when we measured mKO fluorescence intensity in post-mitotic GSC-GB pairs, both cells are actively undergoing S phase for the next mitosis and exhibit robust incorporation of mKO-labeled newly synthesized histones (Figure 4C). Second, any histone turn-over that incorporates newly synthesized mKO-labeled histones (Deal et al., 2010; Dion et al., 2007) during processes such as transcription may not be sister chromatid-specific.” In the quantification shown in Figure 2D, the first reason does not apply since here the quantification is done using mitotic ISCs at anaphase and telophase. However, the second reason still applies. In addition, in case ISCs undergo more than one cell cycle, the GFP-tagged histone is still reliably old but some of the mCherry-tagged histone could be old, too.

On the other hand, in post-mitotic ISC-EB pair, since ISC can undergo subsequent S phase and EB cells could undergo endocycling to become ECs. However, these cases could be captured by EdU pulse; additionally, the endocycling ECs would be polyploidy detectable with the DAPI staining. Both situations would be excluded from data analyses (see Methods). Therefore, because of these cell cycle progression differences between the two stem cell systems, we can study new histone patterns in the post-mitotic cells in the ISC system with higher confidence than in the male germline system. Therefore, we added new H3 quantification in post-mitotic ISC-EB pairs in this work.

6. In Figure 4I- The proliferation status is very much linked to physiological status of the fly, is often variable and therefore, N=4 midguts is not enough. They should quantify PH3 in a larger number of guts and plot the individual data points with the mean or a box plot. This should be examined in separate experiments with a higher number of guts (N>15).

We actually did compute the mitotic index using 11 midguts, which could be missed by this reviewer. We clarified it in the revision (see legend for revised Figure 5I).

7. I feel that the model figure 6C is an over-interpretation. While there appears to be a small increase in the number of dividing cells, subsequently resulting in more esgGFP+ cells, I am not convinced that this is a "tumor" of any kind. They do not present any evidence of a differentiation defect or large accumulation of stem cells. I would tone down this model.

We toned down this model and called this phenotype stem cell hyperplasia instead of tumor (see revised Figure 7).

Referee #2:

This work provides evidence that biased incorporation of new vs old histone H3/H4 occurs along the entire length of each sister chromatid in fly intestinal stem cells. The resultant two sister chromatids, one incorporated with more old H3/H4 and the other incorporated with more newer H3/H4, are segregated during subsequent cell division. When ISCs divide asymmetrically, the sister chromatid with old H3/H4 stays in a future ISC and the other sister chromatid with new H3/H4 is inherited by a differentiating daughter cell. When ISCs divide symmetrically, these two sisters still show biased incorporation of new vs old H3/H4 but they segregate randomly. Moreover, when this segregation pattern was compromised by expressing non-phosphorylatable H3 (H3T3A), ISC-like cells overproliferate.

Along with a series of works published from the same group, this work provides important information regarding the intrinsic regulation of cell-fate determination during asymmetric stem cell division, such that two sister chromatids made in stem cells are already different right after they are synthesized, even before the cell division. The authors used fly intestinal stem cells which can divide either symmetrically or asymmetrically and demonstrated that the H3/H4 inheritance pattern is correlated to ISC division modes (asymmetric or symmetric). This work, together with their older works, suggests that chromosomes (or sister chromatids) distinctly marked by new vs old histones may contain distinct epigenetic information, and is conserved and linked to cell fate determination in different stem cell systems and as such will be of broad interest to cell and developmental biologists.

We appreciate this reviewer's comprehensive summary and the overall positive comments. Indeed, as this reviewer insightfully pointed out: "The intrinsic regulation of cell-fate determination during asymmetric stem cell division, such that two sister chromatids made in stem cells are already different right after they are synthesized, even before the cell division." And the potential conservation identified between *Drosophila* male germline stem cells and intestinal stem cells (in female midguts) will advance our understanding of the intrinsic mechanisms that regulate adult stem cell identity and activity.

Major points;
What is the mechanism of asymmetric incorporation of new/old H3? Authors previously suggested that replication forks may be unidirectional in the male germline. Do the authors consider that a similar mechanism is used in ISCs?

We really appreciate this reviewer's very insightful comments. Indeed, we speculate that DNA replication contributes to asymmetric incorporation of new *versus* old H3. However, studying the detailed mechanisms, in particular the directionality of replication fork movement, would be beyond the scope of this paper.

Histone H3 expression is normally limited in S-phase. I would imagine esg-Gal4 driven expression is quite different from endogenous H3 dynamics. I suggest considering using endogenous locus.

This reviewer is absolutely right that the expression of the transgenic H3 could be different from endogenous H3 expression. However, we showed in previous publications that the incorporation of new transgenic H3 is S-phase-dependent in both male and female germline stem cells in *Drosophila* (Kahney *et al*, 2021; Tran *et al*, 2012), as well as in mouse embryonic stem cells (Ma *et al*, 2020). In contrast, transgenic histone variants, such as H3.3 and CENP-A, show S-phase-independent incorporation, as demonstrated in the above publications as well as in (Ranjan *et al*, 2019). Therefore, even though there is a difference in transgenic and endogenous canonical histone gene expression, there is no detectable difference in their protein incorporation mode. In the revision, we included images showing that new H3 incorporation depends on S phase. Here, a short-term recovery post heat shock does not lead to robust new H3 incorporation, in contrast to the robust new H3.3 incorporation, which is replication-independent (see revised Figure EV1B).

The method of labeling system is unclear. How did authors choose the 18-hour time point? Authors should provide the data to show 18-hour point is in next round of S-phase after the flipping event occurred. In addition, authors should provide the percentage of cells that are "flipped-out" Does this proportion change if they use a timepoint other than "18 hours" and if so, how do they interpret those results?

We added more explanation to the revision, which indicate that 18 hours post heat shock is a “sweet” time point: In a time-course experiment, at 12 hours post heat shock we detected little to no new histone incorporation in most ISCs; and at 36 hours post heat shock most ISCs show all new histone with little old histone signals (see revised Figure EV1A). In contrast, at 18-24 hours post heat shock we could detect robust old and new H3 in the majority of ISCs. However, not all ISCs undergo cell cycle in synchrony, at 18-24 hours post heat shock, there were still some ISCs with little to no mCherry-labeled new H3, which should be cells that have not undergone S phase after heat shock or have not flipped. There were also few ISCs with little to no GFP-labeled old H3, these cells might have gone through more than one cell cycle after heat shock. However, these cases were not included in the data quantification (see Methods).

Referee #3:

Zion, Emily H et al present meaningful and original work, indicating that histone inheritance patterns regulate cell fates in adult stem lineages in vivo. Since proper differentiation of intestinal stem cells is essential for intestinal homeostasis, the findings are significant and have the potential to impact several fields. In addition, the authors clarified that compromising asymmetric histone inheritance led to an increase in Delta-symmetric cell pairs and tumors. Generally, the experiments are well designed and the data are powerful. I have some comments and concerns about the experiments and interpretation, which the authors may want to consider for improving the manuscript.

We appreciate this reviewer's very positive comments. We have seriously considered this reviewer's suggestions and here are our answers.

1. In Figure 1, the authors utilized "FRT/FLP" system to study histone distribution and inheritance patterns during ISC divisions. Is there a leakage in the system even without heat shock? If there is a leak, it may affect the results. I would hope that the authors check the leakage of "FRT/FLP" system.

We thank this reviewer's suggestions. We have done this experiment and provided the no heat shock control (see revised Figure EV1A), which show minimal pre-flipped cells. Additionally, even if an ISC has undergone pre-flip prior to the heat shock treatment, it will lead to cells with almost exclusively new histones, which would be excluded from our data analysis (see Methods).

2. In all Figures, old histone and new histone signals were shown with white color, which is not good for reading. I would suggest that the old histone signal is showed a green color and the new histone signal is showed a red color.

In all Figures, we showed old histone and new histone signals separately with white color to clearly demonstrate their signal strength. But in the merged images, old histone signals are in green and new histone signals are in red. We think this additional monochrome display is more clear for readers, but we could change this in accordance to the Journal's figure display policy.

3. In Figure 1C, Figure S1B, D, E, and Figure SG-H, genotypes are not written in italics. Hopefully, the author will go into more careful about this.

We thank this reviewer's comments and changed all genotypes to be in italics throughout the figure panels.

4. In Figure 4 H, I and Figure 5 A-D, the authors used white color to present DAPI signal, which is not good for reading. Whether can replace the white color with blue color to show the DAPI?

We could change this color scheme but when we did, the blue color for DAPI signals is hard to see, especially on prints. The white color is easier to visualize. But again, we can change this in accordance to the Journal's figure display policy.

5. In Figures 4 and 5, the authors found that disruption of asymmetric histone inheritance patterns resulted in increased Delta-positive ISC-like cells. Is it the disruption of asymmetrical division that leads to the mis-differentiation of ISCs, resulting in ISC accumulation? Compare the number of differentiated cells (ECs or EEs) in wild-type and histone mutants.

We thank this reviewer's suggestion. We have done these experiments to quantify EEs and ECs. We provided these results in the revised Figure 6D. We have also performed scRNA-seq using H3- and H3T3A-expressing midguts, through which we confirm that spatiotemporally controlled expression of the mutant histone H3T3A does not affect major cell types in the ISC lineage, such as ECs or ees, but compromises differentiation of ISCs (see revised Figure 6E-J, Figure EV4).

Referee #4:

The manuscript 'Histone inheritance patterns in Drosophila intestinal stem cells' by Zion et al. studies the distribution of H3 in intestinal stems cells (ISC) of the Drosophila midgut. In this manuscript the used a ISC-specific Gal4 driver to express their previously used dual labelling system specifically in ISCs. The authors find that there is an unequal distribution of new H3 and old H3 that was already present at the previous cell cycle. They have previously used this system to show an asymmetric distribution in germ line stem cells. The find in both systems that newly made proteins tend to get preferentially inherited in the differentiating cells whereas the previously present protein remains in the stem cell. This is a very neat system that the authors have developed a few years ago and shown that is works well to distinguish histone inheritance. The results in this manuscript are clear and the experiments well executed and in a technically sound manner. The quality of the presented results is very good. However, the manuscript lacks any major new advances in our understanding of stem cell chromatin inheritance as most of their results have been described in similar (but not identical) ways before and as detailed below.

We thank this reviewer's overall positive comments. But we would disagree with the comments that this work is similar to previous published studies. First, even though the asymmetric inheritance of H3 and H4 are similar to the previous published results in *Drosophila* germline stem cells, the current results demonstrate that this phenomenon is conserved in *Drosophila* intestine stem cells, a somatic adult stem cell lineage. Given the differences between these two systems, this new finding has advanced our current understanding of chromatin regulation in adult stem cell systems, which has been commented by other reviewers. Second, the previous publication on asymmetric old versus new CENP-A (CID in *Drosophila*) is on a histone H3 variant specifically localized at the centromere region. CENP-A has very distinct biological functions and replication-independent incorporation mode compared to the canonical histones, as reported in this work. We now discussed more about the relationships of these findings in the context of the results reported in this work. However, we strongly think all these findings are in a complementary but not redundant manner, which contribute to a better understanding of chromatin regulation in adult stem cells.

The authors claim that they see the unequal distribution of old and new H3 histones already in prometaphase as this is the time when chromosomes start to align. What wasn't clear from the manuscript is whether the transgenes are incorporated into the ISC during replication like the endogenous proteins or whether they either get incorporated outside of replication or are actually not incorporated at all but only associated with chromatin. The authors must make it clear from which results they can conclude that their tagged versions are also replication dependent deposited onto chromatin and how this then translate into an asymmetric distribution in prometaphase.

This reviewer makes a great point that the expression of the transgenic H3 could be different from endogenous H3 expression. However, we showed in previous publications that the incorporation of new transgenic H3 is S-phase-dependent in both male and female germline stem cells in *Drosophila* (Kahney *et al.*, 2021; Tran *et al.*, 2012), as well as in mouse embryonic stem cells (Ma *et al.*, 2020). In contrast, transgenic histone variants, such as H3.3 and CENP-A, show S-phase-independent incorporation, as demonstrated in the above publications as well as in

(Ranjan *et al.*, 2019). During mitosis, sister chromatids enriched with old vs. new H3 are differentially condensed, leading to their separable domains in prophase and prometaphase (Ranjan *et al*, 2022). Therefore, even though there is a difference in transgenic and endogenous canonical histone gene expression, there is no detectable difference in their protein incorporation mode.

In this revision, we included images showing that new H3 incorporation depends on S phase in the intestine stem cells as well. Here, a short-term recovery post heat shock does not lead to robust new H3 incorporation, in contrast to the robust new H3.3 incorporation, which is replication-independent (see revised Figure EV1B). Due to this different incorporation mode between replication-dependent canonical histone and replication-independent histone variant, sister chromatids carrying asymmetric old vs. new H3 are differentially condensed, leading to detectable separable domains enriched with old vs. new H3. However, studying the detailed mechanism underlying replication-dependent differential histone incorporation in the intestinal stem cell system and comparing with the mechanisms identified in the germline stem cell system would be beyond the scope of this paper. Finally, in all our images, fluorescence tagged histones co-localize with other markers such as DNA dye (DAPI) and mitotic chromatin marker (H3S10ph). Moreover, they display different age-dependent behaviors, even with the same fluorescence tag, as shown in the revised Figure 1E-F, using the co-expression control. All these data demonstrate that their distinct behavior is due to their differential incorporation based on age difference (i.e., old vs. new). Recently, we had a preprint that showed that fluorescence tagged histones are indeed incorporated into chromatin using Chromatin ImmunoCleavage (ChIC)-seq/CUT&RUN assay (https://biorxiv.org/cgi/content/short/2022.09.03.506490v1).

The authors mention in their manuscript that the same inheritance pattern has been shown before in a different stem cell linage in the same organisms but they fail to clearly mention that it is a similar inheritance pattern that has been described in the same intestinal stem cells with the histone H3 variant CID using the same dual labelling technique. This is important since they authors claim in their introduction that it isn't known whether asymmetric histone inheritance is a germ line-specific event or also occurs in other stem cells like the ISC ("The question remains, however, of whether this phenomenon is germ cell-specific or if it serves as a more general mechanism."). They continue to ignore that asymmetric inheritance has already been established in vivo in ISC ('We propose that the ISC lineage is a great system to study histone inheritance due to its well-characterized lineage, clearly distinguishable ISC-specific mitosis, and abundant ISCs in vivo.').

We further discussed the previous publication on asymmetric old versus new CENP-A (CID in *Drosophila*) in the same intestinal stem cell system. As commented above, CID is a histone H3 variant specifically localized at the centromere region. CID has very distinct biological functions compared to the canonical histones and its incorporation is replication-independent, which is different from the replication-dependent canonical histones, as reported in this work. We want to clarify that these statements are made specifically for canonical histones but not for histone variant. We revised accordingly to avoid any potential confusion or misunderstanding.

What is somewhat difficult to understand is what the differences are in Figure 1, 2 and 3 in terms of their results. All histones and also the Delta staining could easily have been shown in one

figures especially because this is mainly recaps from previous papers from the lab in a different stem cell line.

This is concerning the presentation style of the results, for which we demonstrated them in a step-wise manner: Figure 1 introduces the experimental design, as well as the separable old and new histone using the tag-switch system vs. using a self-cleaving tag control in prophase and prometaphase. Figure 2 shows other canonical histones in prophase and prometaphase, as well as all key canonical histones in anaphase and telophase. Figure 3 studies old vs. new histone patterns in post-mitotic pairs. All these imaging results are accompanied with quantifications. If we combine all of them, including the relevant Figure S2, it would be too big to be one figure. Moreover, since this is a central finding of this work, it would be reader friendly to introduce the experimental design and demonstrate the results one-by-one, since we cannot assume that general readers are as familiar with the previous publications as this reviewer.

Also in Figure 4, most of their results were in clear accordance with what they have seen in male GSC. What they could have done here to potentially obtain new results that wouldn't be possible in GSC is the infection of Drosophila with gut pathogens that change SCD and ACD. It would be interesting to know if the H3T3 mutant would lead to a more severe phenotype.

This is a very interesting suggestion, which we will explore in our future studies. But it is beyond the scope of this paper.

I am not sure about their terminology of their results of the histone H3T3A mutant. The inheritance seems fine but there are more symmetric SCD. This is also observed when the Drosophila gut is injured or infected but I would not call it 'mis-inheritance', the inheritance is fine but the gut seems to produce more stem cells just like after infection. I also think that the 'ISC tumor formation' is not necessarily backed up by experiments in this paper. There is a clear overproliferation but overproliferation alone doesn't make a tumor.

We have revised accordingly and called this phenotype stem cell hyperplasia instead of tumor.

Minor Points:

The first paragraph of the introduction is too broad. For instance, why do the authors mention DNA methylation when the model organism of this paper doesn't have DNA methylation? A more to the point introduction would benefit the paper.

We revised this part to make it more relevant with the data presented in this work.

References:

1. Kahney EW, Zion EH, Sohn L, Viets-Layng K, Johnston R, Chen X (2021) Characterization of histone inheritance patterns in the Drosophila female germline. *EMBO Rep*: e51530
2. Ma B, Trieu TJ, Cheng J, Zhou S, Tang Q, Xie J, Liu JL, Zhao K, Habib SJ, Chen X (2020) Differential Histone Distribution Patterns in Induced Asymmetrically Dividing Mouse Embryonic Stem Cells. *Cell Rep* 32: 108003
3. Ranjan R, Snedeker J, Chen X (2019) Asymmetric Centromeres Differentially Coordinate with Mitotic Machinery to Ensure Biased Sister Chromatid Segregation in Germline Stem Cells. *Cell Stem Cell* 25: 666-681 e665
4. Ranjan R, Snedeker J, Wooten M, Chu C, Bracero S, Mouton T, Chen X (2022) Differential condensation of sister chromatids acts with Cdc6 to ensure asynchronous S-phase entry in Drosophila male germline stem cell lineage. *Dev Cell* 57: 1102-1118 e1107
5. Tran V, Lim C, Xie J, Chen X (2012) Asymmetric division of Drosophila male germline stem cell shows asymmetric histone distribution. *Science* 338: 679-682

Dear Xin,

Thank you for the submission of your revised manuscript. We have now received the enclosed reports from referees 1-3. Both referees 1 and 2 still have remaining concerns that will need to be addressed in a satisfactory manner before we can proceed with the official acceptance of your manuscript.

A few editorial requests will also need to be addressed:

- Please reduce the number of keywords to 5.

- Please correct the conflict of interest subheading to "Disclosure and Competing Interest Statement"

- Please remove the author credits from the ms file. We now use CRediT to specify the contributions of each author in the journal submission system. CRediT replaces the author contribution section. Please use the free text box to provide more detailed descriptions, if you wish. See also guide to authors https://www.embopress.org/page/journal/14693178/authorguide#authorshipguidelines.

- Fig 3M-P are called out before 3D. Fig EV4A-H callouts are missing. Please correct.

- The ms sections are in the wrong order, please correct.

- Please remove EV tables and their legends from the ms file. These should be uploaded individually as EV Table or Dataset (multiple sheets/more than 5 columns/20 rows) files. Colour may only be used in EV Tables.

- The file of supplemental Materials & Methods and References needs to be uploaded as an Appendix file. Otherwise, this information could be included in the main ms file.

- I attach to this email a related ms file with comments by our data editors. Please address all comments in the final ms file.

I would like to suggest some minor changes to the title and abstract. Please let me know whether you agree with the following:

Old and newly synthesized histones are asymmetrically distributed in Drosophila intestinal stem cell divisions

We report that preexisting (old) and newly synthesized (new) histones H3 and H4 are asymmetrically partitioned during the division of Drosophila intestinal stem cells (ISCs). Furthermore, the inheritance patterns of old and new H3 and H4 in post-mitotic cell pairs correlate with distinct expression patterns of Delta, an important cell fate gene. To understand the biological significance of this phenomenon, we expressed a mutant H3T3A to compromise asymmetric histone inheritance. Under this condition, we observe an increase in Delta-symmetric cell pairs and overpopulated ISC-like, Delta positive cells. Single cell RNAs-seq assays further indicate that H3T3A expression compromises ISC differentiation. Together, our results indicate that asymmetric histone inheritance potentially contributes to establishing distinct cell identities in a somatic stem cell lineage, consistent with previous findings in Drosophila male germline stem cells.

EMBO press papers are accompanied online by A) a short (1-2 sentences) summary of the findings and their significance, B) 2-3 bullet points highlighting key results and C) a synopsis image that is exactly 550 pixels wide and 200-600 pixels high (the height is variable). You can either show a model or key data in the synopsis image. Please note that text needs to be readable at the final size. Please send us this information along with the final manuscript.

I look forward to seeing a final version of your manuscript as soon as possible. Please use this link to submit your revision: https://embor.msubmit.net/cgi-bin/main.plex

Best wishes,
Esther

Esther Schnapp, PhD
Senior Editor
EMBO reports

Referee #1:

I have made pdf that will send you as it is easier since I color-coded old and new comments.

Referee #2:

In my previous review, I requested as follows:
"The method of labeling system is unclear. How did authors choose the 18-hour time point? Authors should provide the data to show 18-hour point is in next round of S-phase after the flipping event occurred. In addition, authors should provide the percentage of cells that are "flipped-out" Does this proportion change if they use a timepoint other than "18 hours" and if so, how do they interpret those results?"

The response sounds reasonable but I wanted to see labeling results more quantitatively including the percentages of cases of labeling they observed in the tissue including which region of intestine was scored and how many cells are labeled to be selected for each category in their table.

After authors address this point, I fully support this manuscript to be published in embo report.

Referee #3:

The authors have made a significant effort and answer of my concerns in this revised version of the manuscript. I have no questions.

Final and clarifying response to both referees 1 and 2:

Major points:

1. How many pairs of cells were quantified per gut?

Referee 1: I am not sure what wanted to see is provided, though it is possible that I missed these data somewhere. What I asked for was "the numbers of cells that they quantified in both cases (esgGal4 vs esgGal4 Su(H)Gal80) per gut. I would like to evaluate how many pairs are quantified per gut. If I missed it, please state exactly where these data are. If they are not included, please indicate approximate numbers of pairs that are typically quantified PER GUT.

These data are now all presented in Figure 3. In the Figure 3 legend, we provided detailed information how many pairs were quantified from how many intestines, which are highlighted in Figure 3 legend.

2. Data supporting that H3 incorporation depends on S-phase.

Referee 1: Please state the figure that includes the images showing that H3 incorporation depends on S phase. I disagree that the H3 incorporation serves the same purpose as EdU feeding: EdU incorporation has been extensively characterized in the literature and is associated with S phase. The point I was raising was to have an independent line of evidence suggesting that H3 incorporation depends on DNA synthesis. If I missed it, please clearly state where these data are shown.

Referee 2: The method of labeling system is unclear. How did authors choose the 18-hour time point? Authors should provide the data to show 18-hour point is in next round of S-phase after the flipping event occurred. In addition, authors should provide the percentage of cells that are "flipped-out". Does this proportion change if they use a timepoint other than "18 hours" and if so, how do they interpret those results? The response sounds reasonable but I wanted to see labeling results more quantitatively including the percentages of cases of labeling they observed in the tissue including which region of intestine was scored and how many cells are labeled to be selected for each category in their table.

These data are now presented in Figure EV1: First of all, we thank Referee 1 for suggesting the EdU feeding assay, which would work if the fed EdU gets incorporated in ISC during S phase and could be chased through the subsequent M phase, when EdU could be detected in the post-mitotic daughter pair. However, the caveat is that the EdU added to the food cannot be completely washed off. In another word, the time frame of this pulse could easily go beyond the previous S phase before ISC cell division. And after cell division, ISC could enter the next S phase and incorporates EdU again. Therefore, even though we performed EdU pulse experiments (see “Quantification of histone signals in post-mitotic pairs” in Materials and Methods from Supplemental Information), we used it to eliminate ISCs that enter the next S phase since this could lead to new histone incorporation irrelevant to the old vs. new histone inheritance from the previous cell cycle. In summary, we completely agree that EdU incorporation is a reliable assay for active DNA synthesis. However, adding it to the food has the caveat for this pulse label to be chased. On the other hand, it has been well established the bulk of newly synthesized canonical histones are incorporated in a replication-dependent manner. For example, in this earlier work in *Drosophila* (Ahmad & Henikoff, 2002), the researchers used GFP-tagged H3 *versus* GFP-tagged

H3.3 and showed that H3 is exclusively incorporated in a replication-dependent manner whereas H3.3 is incorporated in a replication-independent manner. Therefore, genome-wide incorporation of the newly synthesized canonical histones can be used to show that cells have gone through S phase. Consistent with the different incorporation modes between H3 and H3.3, we did find that new H3.3 incorporation is continuous whereas new H3 incorporation only occurs after an extended period of time post heat-shock treatment (Figure EV1B).

Second, regarding Referee 2's comments, these points have been addressed in Figure EV1A, where we showed that at different time points post heat-chock (PHS) treatment: At 12 hours PHS, there were very few cells with robust new H3 incorporation; at 36 hours PHS, there were very few cells with robust old H3 signals. Therefore, we chose a time point in between at 18 hours PHS. In reality, as explained above, as ISCs undergo cell cycle progression asynchronously, robust new H3 incorporation with significant old H3 signals is the "sweet spot" for quantifying both signals with confidence. Additionally, since the bulk of newly synthesized canonical histones are incorporated in a replication-dependent manner, even if the cells are "flipped-out" genetically, without going through a complete round of S phase, new H3 protein is undetectable. Because of this, we cannot "provide the percentage of cells that are 'flipped-out'", as this referee suggested at the earlier time points PHS.

In the revision, we provide the percentages of ISCs that are "flipped-out" based on new H3 signals at 0 hour and 18 hours PHS, respectively. These data are now presented in Figure EV1C, showing 2.42% 'flipped-out' at 0 hour and 95.06% 'flipped-out' at 18 hours PHS. These results confirm minimal flipped-out without heat shock but efficient flipped-out at 18 hours PHS, the time point we performed most imaging-based analysis.

Regarding the "region of intestine that was scored": We used the entire midgut region and excluded the foregut and hindgut regions of the intestine in all our assays.

Regarding "how many cells are labeled to be selected for each category in their table", these are detailed in the corresponding figure legends.

Reference:

Ahmad K, Henikoff S (2002) The histone variant H3.3 marks active chromatin by replication-independent nucleosome assembly. *Mol Cell* 9: 1191-1200

Dr. Xin Chen
HHMI, Johns Hopkins University
Biology Department
Levi Hall 137
3400 North Charles Street
Baltimore, MD 21218-2685
United States

Dear Xin,

I am very pleased to accept your manuscript for publication in the next available issue of EMBO reports. Thank you for your contribution to our journal.

At the end of this email I include important information about how to proceed. Please ensure that you take the time to read the information and complete and return the necessary forms to allow us to publish your manuscript as quickly as possible.

As part of the EMBO publication's Transparent Editorial Process, EMBO reports publishes online a Review Process File to accompany accepted manuscripts. As you are aware, this File will be published in conjunction with your paper and will include the referee reports, your point-by-point response and all pertinent correspondence relating to the manuscript.

If you do NOT want this File to be published, please inform the editorial office within 2 days, if you have not done so already, otherwise the File will be published by default [contact: emboreports@embo.org]. If you do opt out, the Review Process File link will point to the following statement: "No Review Process File is available with this article, as the authors have chosen not to make the review process public in this case."

Should you be planning a Press Release on your article, please get in contact with emboreports@wiley.com as early as possible, in order to coordinate publication and release dates.

Thank you again for your contribution to EMBO reports and congratulations on a successful publication. Please consider us again in the future for your most exciting work.

Best regards,
Esther

Esther Schnapp, PhD
Senior Editor
EMBO reports

****

THINGS TO DO NOW:

Please note that you will be contacted by Wiley Author Services to complete licensing and payment information. The required 'Page Charges Authorization Form' is available here: https://www.embopress.org/pb-assets/embo-site/er_apc.pdf - please download and complete the form and return to embopressproduction@wiley.com

EMBO Press participates in many Publish and Read agreements that allow authors to publish Open Access with reduced/no publication charges. Check your eligibility: https://authorservices.wiley.com/author-resources/Journal-Authors/open-access/affiliation-policies-payments/index.html

You will receive proofs by e-mail approximately 2-3 weeks after all relevant files have been sent to our Production Office; you should return your corrections within 2 days of receiving the proofs.

Please inform us if there is likely to be any difficulty in reaching you at the above address at that time. Failure to meet our deadlines may result in a delay of publication, or publication without your corrections.

All further communications concerning your paper should quote reference number EMBOR-2022-56404V3 and be addressed to emboreports@wiley.com.

Should you be planning a Press Release on your article, please get in contact with emboreports@wiley.com as early as possible, in order to coordinate publication and release dates.

EMBO Press Author Checklist

Corresponding Author Name: Xin Chen	
Journal Submitted to: EMBO Reports	
Manuscript Number: EMBOR-2022-56404V1	

USEFUL LINKS FOR COMPLETING THIS FORM
The EMBO Journal - Author Guidelines
EMBO Reports - Author Guidelines
Molecular Systems Biology - Author Guidelines
EMBO Molecular Medicine - Author Guidelines

Reporting Checklist for Life Science Articles (updated January
This checklist is adapted from Materials Design Analysis Reporting (MDAR) Checklist for Authors. MDAR establishes a minimum set of requirements in transparent reporting in the life sciences (see Statement of Task: 10.31222/osf.io/9sm4x). Please follow the journal's guidelines in preparing your manuscript.
Please note that a copy of this checklist will be published alongside your article.

Abridged guidelines for figures
1. Data
The data shown in figures should satisfy the following conditions:
- the data were obtained and processed according to the field's best practice and are presented to reflect the results of the experiments in an accurate and unbiased manner.
- ideally, figure panels should include only measurements that are directly comparable to each other and obtained with the same assay.
- plots include clearly labeled error bars for independent experiments and sample sizes. Unless justified, error bars should not be shown for technical
- if n<5, the individual data points from each experiment should be plotted. Any statistical test employed should be justified.
- Source Data should be included to report the data underlying figures according to the guidelines set out in the authorship guidelines on Data

2. Captions
Each figure caption should contain the following information, for each panel where they are relevant:
- a specification of the experimental system investigated (eg cell line, species name).
- the assay(s) and method(s) used to carry out the reported observations and measurements.
- an explicit mention of the biological and chemical entity(ies) that are being measured.
- an explicit mention of the biological and chemical entity(ies) that are altered/varied/perturbed in a controlled manner.
- the exact sample size (n) for each experimental group/condition, given as a number, not a range;
- a description of the sample collection allowing the reader to understand whether the samples represent technical or biological replicates (including how many animals, litters, cultures, etc.).
- a statement of how many times the experiment shown was independently replicated in the laboratory.
- definitions of statistical methods and measures:
  - common tests, such as t-test (please specify whether paired vs. unpaired), simple χ2 tests, Wilcoxon and Mann-Whitney tests, can be unambiguously identified by name only, but more complex techniques should be described in the methods section;
  - are tests one-sided or two-sided?
  - are there adjustments for multiple comparisons?
  - exact statistical test results, e.g., P values = x but not P values < x;
  - definition of 'center values' as median or average;
  - definition of error bars as s.d. or s.e.m.

Please complete ALL of the questions below.
Select "Not Applicable" only when the requested information is not relevant for your study.

Materials

Newly Created Materials	Information included in the manuscript?	In which section is the information available? (Reagents and Tools Table, Materials and Methods, Figures, Data Availability Section)
New materials and reagents need to be available; do any restrictions apply?	Yes	Materials and Methods

Antibodies	Information included in the manuscript?	In which section is the information available? (Reagents and Tools Table, Materials and Methods, Figures, Data Availability Section)
For **antibodies** provide the following information:<br>- Commercial antibodies: RRID (if possible) or supplier name, catalogue number and or/clone number<br>- Non-commercial: RRID or citation	Yes	Materials and Methods

DNA and RNA sequences	Information included in the manuscript?	In which section is the information available? (Reagents and Tools Table, Materials and Methods, Figures, Data Availability Section)
**Short novel DNA or RNA including primers, probes**: provide the sequences.	Not Applicable	

Cell materials	Information included in the manuscript?	In which section is the information available? (Reagents and Tools Table, Materials and Methods, Figures, Data Availability Section)
**Cell lines:** Provide species information, strain. Provide accession number in repository **OR** supplier name, catalog number, clone number, and/**OR** RRID.	Not Applicable	
**Primary cultures:** Provide species, strain, sex of origin, genetic modification status.	Not Applicable	
Report if the cell lines were recently **authenticated** (e.g., by STR profiling) and tested for mycoplasma contamination.	Not Applicable	

Experimental animals	Information included in the manuscript?	In which section is the information available? (Reagents and Tools Table, Materials and Methods, Figures, Data Availability Section)
**Laboratory animals or Model organisms:** Provide species, strain, sex, age, genetic modification status. Provide accession number in repository **OR** supplier name, catalog number, clone number, **OR** RRID.	Yes	Materials and Methods
**Animal observed in or captured from the field:** Provide species, sex, and age where possible.	Not Applicable	
Please detail **housing and husbandry conditions.**	Yes	Materials and Methods

Plants and microbes	Information included in the manuscript?	In which section is the information available? (Reagents and Tools Table, Materials and Methods, Figures, Data Availability Section)
**Plants:** provide species and strain, ecotype and cultivar where relevant, unique accession number if available, and source (including location for collected wild specimens).	Not Applicable	
**Microbes:** provide species and strain, unique accession number if available, and source.	Not Applicable	

Human research participants	Information included in the manuscript?	In which section is the information available? (Reagents and Tools Table, Materials and Methods, Figures, Data Availability Section)
If collected and within the bounds of privacy constraints report on age, sex and gender or ethnicity for all study participants.	Not Applicable	

Core facilities	Information included in the manuscript?	In which section is the information available? (Reagents and Tools Table, Materials and Methods, Figures, Data Availability Section)
If your work benefited from core facilities, was their service mentioned in the acknowledgments section?	Not Applicable	

Design

Study protocol	Information included in the manuscript?	In which section is the information available? (Reagents and Tools Table, Materials and Methods, Figures, Data Availability Section)
If study protocol has been **pre-registered, provide DOI in the manuscript**. For clinical trials, provide the trial registration number **OR** cite DOI.	Yes	doi: https://doi.org/10.1101/2020.08.15.252403
Report the **clinical trial registration number** (at ClinicalTrials.gov or equivalent), where applicable.	Not Applicable	

Laboratory protocol	Information included in the manuscript?	In which section is the information available? (Reagents and Tools Table, Materials and Methods, Figures, Data Availability Section)
Provide DOI OR other citation details if **external detailed step-by-step protocols** are available.	Yes	10X Genomics Single Cell RNA Seq Protocol : https://cdn.10xgenomics.com/image/upload/v1660261285/support-documents/CG000204_ChromiumNextGEMSingleCell3_v3.1_Rev_D.pdf

Experimental study design and statistics	Information included in the manuscript?	In which section is the information available? (Reagents and Tools Table, Materials and Methods, Figures, Data Availability Section)
Include a statement about **sample size** estimate even if no statistical methods were used.	Not Applicable	
Were any steps taken to minimize the effects of subjective bias when allocating animals/samples to treatment (e.g. **randomization procedure**)? If yes, have they been described?	Not Applicable	
Include a statement about **blinding** even if no blinding was done.	Not Applicable	
Describe **inclusion/exclusion criteria** if samples or animals were excluded from the analysis. Were the criteria pre-established?<br><br>If sample or data points were omitted from analysis, report if this was due to attrition or intentional exclusion and provide justification.	Yes	Materials and Methods
For every figure, are **statistical tests** justified as appropriate? Do the data meet the assumptions of the tests (e.g., normal distribution)? Describe any methods used to assess it. Is there an estimate of variation within each group of data? Is the variance similar between the groups that are being statistically compared?	Yes	Materials and Methods

Sample definition and in-laboratory replication	Information included in the manuscript?	In which section is the information available? (Reagents and Tools Table, Materials and Methods, Figures, Data Availability Section)
In the figure legends: state number of times the experiment was **replicated** in laboratory.	Not Applicable	
In the figure legends: define whether data describe **technical or biological replicates**.	Not Applicable	

Ethics

Ethics	Information included in the manuscript?	In which section is the information available? (Reagents and Tools Table, Materials and Methods, Figures, Data Availability Section)
Studies involving **human participants**: State details of **authority granting ethics approval** (IRB or equivalent committee(s), provide reference number for approval.	Not Applicable	
Studies involving **human participants**: Include a statement confirming that **informed consent** was obtained from all subjects and that the experiments conformed to the principles set out in the WMA Declaration of Helsinki and the Department of Health and Human Services Belmont Report.	Not Applicable	
Studies involving **human participants:** For publication of **patient photos**, include a statement confirming that consent to publish was obtained.	Not Applicable	
Studies involving experimental **animals**: State details of **authority granting ethics approva**l (IRB or equivalent committee(s), provide reference number for approval. Include a statement of compliance with ethical regulations.	Not Applicable	
Studies involving **specimen and field samples:** State if relevant **permits** obtained, provide details of authority approving study; if none were required, explain why.	Not Applicable	

Dual Use Research of Concern (DURC)	Information included in the manuscript?	In which section is the information available? (Reagents and Tools Table, Materials and Methods, Figures, Data Availability Section)
Could your study fall under dual use research restrictions? Please check biosecurity documents and list of **select agents and toxins** (CDC): https://www.selectagents.gov/sat/list.htm	Not Applicable	
If you used a select agent, is the security level of the lab appropriate and reported in the manuscript?	Not Applicable	
If a study is subject to dual use research of concern regulations, is the name of the **authority granting approval and reference number** for the regulatory approval provided in the manuscript?	Not Applicable	

Reporting

The MDAR framework recommends adoption of discipline-specific guidelines, established and endorsed through community initiatives. Journals have their own policy about requiring specific guidelines and recommendations to complement MDAR.

Adherence to community standards	Information included in the manuscript?	In which section is the information available? (Reagents and Tools Table, Materials and Methods, Figures, Data Availability Section)
State if relevant guidelines or checklists (e.g., **ICMJE, MIBBI, ARRIVE, PRISMA**) have been followed or provided.	Not Applicable	
For **tumor marker prognostic studies,** we recommend that you follow the **REMARK** reporting guidelines (see link list at top right). See author guidelines, under 'Reporting Guidelines'. Please confirm you have followed these guidelines.	Not Applicable	
For **phase II and III randomized controlled trials**, please refer to the **CONSORT** flow diagram (see link list at top right) and submit the CONSORT checklist (see link list at top right) with your submission. See author guidelines, under 'Reporting Guidelines'. Please confirm you have submitted this list.	Not Applicable	

Data Availability

Data availability	Information included in the manuscript?	In which section is the information available? (Reagents and Tools Table, Materials and Methods, Figures, Data Availability Section)
Have **primary datasets** been deposited according to the journal's guidelines (see 'Data Deposition' section) and the respective accession numbers provided in the Data Availability Section?	Yes	Data Availability
Were **human clinical and genomic datasets** deposited in a public access-controlled repository in accordance to ethical obligations to the patients and to the applicable consent agreement?	Not Applicable	
Are **computational models** that are central and integral to a study available without restrictions in a machine-readable form? Were the relevant accession numbers or links provided?	Not Applicable	
If publicly available data were reused, provide the respective **data citations in the reference list**.	Not Applicable	
